# Identification of Cell Death Genes in Sea Urchin *Paracentrotus lividus* and Their Expression Patterns during Embryonic Development

**DOI:** 10.1093/gbe/evz020

**Published:** 2019-01-29

**Authors:** Christian Galasso, Salvatore D’Aniello, Clementina Sansone, Adrianna Ianora, Giovanna Romano

**Affiliations:** 1 Department of Marine Biotechnology, Stazione Zoologica Anton Dohrn Napoli, Naples, Italy; 2 Department of Biology and Evolution of Marine Organisms, Stazione Zoologica Anton Dohrn Napoli, Naples, Italy

**Keywords:** Paracentrotus lividus, echinoderms, cell death genes, apoptosis, autophagy, development

## Abstract

Apoptosis and autophagy are fundamental mechanisms of programed cell death activated during protostome and deuterostome embryonic development, contributing to the creation and remodeling of different anatomical structures. Programed cell death has been investigated at morphological and biochemical levels, but there is a lack of information concerning gene expression of death factors during deuterostome embryonic development. In this study, we analyze the expression patterns of 13 genes involved in autophagy, extrinsic and intrinsic apoptosis during blastula, gastrula, and pluteus stages of the sea urchin *Paracentrotus lividus* embryonic development. Results suggested the occurrence of all death mechanisms investigated, highlighting the simultaneous involvement of apoptosis and autophagy during embryonic development. In particular, gastrula was the developmental stage where the majority of death genes were highly expressed. During gastrulation apoptotic processes are fundamental for tissue remodeling, such as cavity formation and removal of inner ectodermal cells. This is the first report that identifies a panel of cell death genes in the *P. lividus* genome and analyzes their expression variations during ontogenesis.

## Introduction

Apoptosis is a widespread mechanism of programed cell death (PCD), which is considered a fundamental prerequisite for several processes including normal cell turnover, functioning of the immune system, and elimination of cells carrying DNA aberrations. PCD is also essential for embryonic development, contributing to appropriate formation of body structures (Fuchs and Steller 2011). In particular, organisms activate PCD to eliminate damaged cells and to remodel tissues during morphogenesis and organogenesis (Lockshin and Zakeri 2002). Most of what is known on apoptosis derives from the study of PCD occurring at specific stages of *Caenorhabditis elegans* development (Horvitz 1999). This organism generates 1,090 somatic cells during the formation of the adult worm, but 131 of these cells undergo apoptosis. These 131 cells die through activation of apoptosis in a specific stage, which is common in worms, demonstrating the high accuracy and conservation of this mechanism (Elmore 2007). This controlled cell death mechanism is nowadays well described during development of vertebrate embryos, where it does not occur before gastrulation due to the early expression of antiapoptotic factors, such as Bcl2, defence proteins, such as heat shock proteins, and cytoprotective enzymes, such as glutathione S-transferases (Bloom et al. 1998; Muscarella et al. 1998; Exley et al. 1999). On the other hand, apoptosis in echinoderms is activated during early stages of embryonic development (e.g., blastula) (Vega Thurber and Epel 2007). In general, apoptosis is a highly sophisticated mechanism, which induces an energy-dependent cascade of molecular events. Two main apoptotic processes have been characterized: the extrinsic or death receptor pathway and the intrinsic or mitochondrial pathway. Death-ligand/death-receptor binding activates extrinsic apoptosis, which leads to activation of Caspase 8 (Casp8) and in turn of the executioner Caspase 3 (Casp3). Different extracellular stimuli induce mitochondrial changes, with activation of intrinsic apoptosis, which is characterized by the formation of an apoptosome and subsequently the activation of the Casp3 (Elmore 2007).

Comparable to apoptosis, autophagy is a fundamental process in living organisms for correct growth and maintenance of cellular homeostasis; it is the main process for elimination and recycling of damaged intracellular components. Emerging studies have demonstrated its fundamental role during embryonic development in both protostomes and deuterostomes (Di Bartolomeo et al. 2010; Aburto et al. 2012). This process has been associated with large-scale cell death and removal of whole tissues during development (Agnello et al. 2015). In particular, autophagy is a highly conserved mechanism characterized by the formation, through dynamic membrane rearrangement of vesicles called autophagosomes, able to sequester cytoplasm and organelles. Autophagosomes induce degradation of cellular organelles, and the activation of this mechanism depends on several stimuli, such as nutrients (amino acids), energy (ATP), and growth factor (insulin/IGF) (Yang and Klionsky 2009).

Apoptosis and autophagy are genetically regulated processes, required for the control of cell fate. Despite the fact that the two mechanisms involve molecular factors and signaling cascades markedly different, their regulatory machinery is intimately connected. Recent studies have demonstrated that p53, a well-known proapoptotic factor, can also induce autophagy (Crighton et al. 2006). On the contrary, activation of the PI3 kinase/Akt pathway, which is an inhibitor of apoptosis, also inhibits autophagy. In addition, other key factors of the apoptosis or autophagy machinery, including Bcl family members, FADD, and some Atg proteins can directly regulate both processes (Thorburn 2008).

Sea urchins have been widely used as an experimental model to investigate evolutionary conserved molecular pathways and gene regulatory networks during embryogenesis, because it does not undergo a whole-genome duplication. This characteristic, therefore, renders sea urchins an ideal model for functional analyses (Sea Urchin Genome Sequencing Consortium 2006).

The main death factors have been previously identified and annotated in the sea urchin *Strongylocentrotus purpuratus* genome (Sea Urchin Genome Sequencing Consortium 2006). In particular, *S. purpuratus* possesses 31 Caspases, 10 Bcl2 genes, and 7 tumor necrosis factor receptors (TNFRs), showing a greater diversification of apoptotic genes with respect to nematodes and arthropods (Robertson et al. 2006). However, studies aimed to examine variations of PCD genes expression in different phases of embryonic development in sea urchins are lacking. Therefore, here we investigate gene expression profiles related to death mechanisms (autophagy, extrinsic and intrinsic apoptosis) during development of the sea urchin *Paracentrotus lividus* for which there is an ongoing genome assembly project.

The molecular interactions between the three death pathways remain partially obscure and thus of great interest. In this perspective, the sea urchin genomes could give a strong impulse to better clarify the evolution and increasing complexity of PCD. The aim of this study is the identification, through phylogenetic analyses, of orthologous death genes in *P. lividus* and the description of their temporal expression pattern during ontogenesis. To the best of our knowledge, this work is the first study of death signaling pathways in *P. lividus*, which includes the identification and temporal expression analyses of 13 genes related to extrinsic and intrinsic apoptosis, as well as autophagy, during three key stages of embryonic development (i.e., blastula, gastrula, and pluteus).

## Materials and Methods

### Identification of Apoptotic Genes in the Sea Urchin *Paracentrotus lividus* Genome

Amino acid sequences of death factors of interest from *Homo sapiens*, *S. purpuratus* (phylogenetically closer to *P. lividus*), and other deuterostomes were used as queries to interrogate the *P. lividus* genome database (TBlastN, (v3.0, Genoscope, Sept 2014). Once identified, the genomic contigs or scaffolds containing the putative orthologous genes were processed with GenScan (Burge and Karlin 1997), which produced a gene and the relative protein sequence prediction with a good approximation (see supplementary information I, Supplementary Material online). Further validation of the correct orthology was obtained by reciprocal BlastP in NCBI database and by phylogenetic analysis. The combination of these steps including reciprocal BlastP, multiple alignment, and phylogenetic analysis was essential to confirm that our in silico predicted sequences were reliable.

### Phylogenetic Analysis

Phylogenetic analyses were necessary not only to identify death factors belonging to superfamilies with multiple members, such as Tnf receptors, Rip kinases, and Ulks, but also to confirm the identity of in silico predicted sequences of all other death genes. The protein sequences employed for the evolutionary survey were retrieved from NCBI. The species included in the phylogeny were *H. sapiens*, *S. purpuratus*, *Saccoglossus kowalevskii*, *Branchiostoma belcheri*, *Ciona robusta* (previously named *Ciona intestinalis*), *Danio rerio*, *Apostichopus japonicus*, *Acanthaster planci*, *Platynereis dumerilii*, *Mizuhopecten yessoensis*, and *Caenorhabditis elegans* (see supplementary information II, Supplementary Material online).

Proteins of interest were aligned by ClustalW (Sievers et al. 2014), and maximum likelihood phylogenetic reconstructions were performed using MEGA6 with 1,000 replicates and the WAG matrix (Tamura et al. 2013). The graphical representation of final trees was performed with Adobe Illustrator.

### Animal Collection and Embryo Cultures

*Paracentrotus* *lividus* were collected from the Gulf of Naples (coordinates of sampling point are: 40°47′50.5″N and 14°12′05.8″E). Sea urchins were transported in a refrigerated box to the laboratory where animals were maintained in large tanks with circulating seawater. To obtain gametes, sea urchins were injected with a 2 M solution of potassium chloride (KCl) and vigorously shaken. Female sea urchins were placed with the anal pore face down on a beaker containing filtered seawater (FSW) to collect eggs. Once eggs were released, they were washed using a cotton lint and FSW. Pool of sperms was collected with a pipet from the anal pore of few male sea urchins. Before starting any experiments, a fertilization test was carried out to evaluate egg and sperm quality and to analyze fertilization efficiency. Eggs were counted with an inverted microscope (Zeiss Axiovert 135TV), and the resulting concentration was used to set up three separate in vitro experiments, using crystallizing dishes containing around 8,000 eggs in 50 ml of FSW. Embryos were incubated at three incubation times (5, 21, and 48 h) in a thermostatic chamber with a 12–12 h light–dark cycle at 20 °C. At the end of specific incubation times, embryo cultures were collected by centrifuging in Falcon tubes at 3,600 × g, for 15 min, at 4 °C. Embryo pellets were quickly frozen in liquid nitrogen and then stored at –80 °C for further analysis.

All experiments were conducted in six replicates, collecting eggs from six different females, each fertilized with a mix of sperm from different males. Replicate experiments were carried out on different days.

### RNA Extraction, cDNA Synthesis, and Polymerase Chain Reaction Amplification

Frozen pellets of *P. lividus* embryos were dissolved in Trisure Reagent (adopting a ratio of 100 μl reagent: 10 mg embryos) for RNA extraction, promoting tissue degradation (Bioline). Direct-zol RNA MiniPrep (Zimo Research) was used to purify total RNA directly from biological samples lysed with Trisure Reagent. RNA concentration was assessed by the absorbance at 260 nm, using the nanophotomer NanodroP (ND-1000 UV-Vis Spectrophotometer; NanoDrop Technologies). Contaminating gDNA was removed by treating each sample with DNase RNase-free kit (Roche), in order to degrade traces of DNA still present in the RNA samples, according to the manufacturer’s instructions. Extracted RNA samples, resuspended in H_2_O plus DEPC (diethylpyrocarbonate, potent inhibitor of RNase), were stored at −80 °C until the reverse transcription step.

The samples were then resolved on a 1% agarose gel in order to assess the integrity of extracted RNA (Sambrook et al. 1989). Agarose gels showed intact rRNA subunits (28S and 18S), indicating no degradation of RNA samples.

Each sample was reverse transcribed (600 ng of total RNA extracted) with iScriptTM cDNA Synthesis kit (Bio-Rad), following the manufacturer’s instructions. Synthesized cDNA was stored at −20 °C and later used in real-time qPCR experiments.

Once all cDNAs were synthesized, polymerase chain reactions (PCRs) were performed using primers previously designed for the zinc-finger transcription factor *Pl*-*Z*12–1 to validate their quality (Costa et al. 2012) using a C1000 Touch Thermal Cycler (Bio-Rad). Each sample was prepared by mixing 1× PCR reaction buffer (Roche), 0.2 mM dNTP, 5 units of Taq DNA Polymerase (Roche), 100 ng/μl of each oligo, 2 μl of template cDNA and nuclease-free water to reach a final volume of 30 μl. The PCR program consisted of an initial denaturation phase at 95 °C for 2 min, followed by 35 cycles (each cycle consisted in three steps: 95 °C for 45 s, 60 °C for 1 min, and 72 °C for 30 s) and a final elongation phase at 72 °C for 10 min. At the end of the PCR process, all samples were run on 1% agarose gel (containing ethidium bromide) to observe the presence of specific band. Size marker was used for fragment size determination.

Primers were designed to amplify regions from 90 to 200 bp size on intron-to-exon boundary, and the Gene Runner program V3.05 (Hasting Software) was used to predict primer melting temperature (Tm) and to check whether they can form dimers, hairpins, bulges, and internal loops (see supplementary information III, Supplementary Material online).

Amplified PCR products were separated by 1% agarose gel and resulting bands were excised from the gel and extracted according to the procedure reported in QIAquick Gel Extraction kit (Qiagen). The specificity of the PCR products was checked by DNA sequencing, using 15 fmol of purified PCR product and 4.5 pmol of each primer in the Beckman CEQ 2000 Automated Sequencer. The results obtained were aligned, through algorithm BLAST, with the relative gene structure used for the primer design.

### Real-Time qPCR

Real-time qPCR experiments were run with all cDNA samples to study quantitative variations of gene expression in three different developmental stages of *P. lividus*. Prior to real-time qPCR experiments, the efficiency and specificity of amplification reactions was assessed for all primer pairs, through melting curve analysis. The efficiency (*E*) of each primer pair was calculated according to standard methods curves, using the equation:

$$E\;=\text{ 1}0^{-\text{1}/\text{slope}}.$$

Starting from a cDNA obtained from control embryos (about 200 ng/μl), five serial dilutions were generated. Using the cycle threshold (*C*_t_) values obtained versus the logarithm of each dilution factor, standard curves were designed for each oligonucleotide pair. PCR efficiencies were found to be 2 for endogenous control and target genes.

Real-time qPCR experiments were set up using undiluted cDNAs as template for the reaction mix containing 0.3 mM for each of the two primers (final concentration) and 1× FastStart SYBR Green master mix (total volume of 10 μl; Applied Biosystems).

All data obtained from real-time qPCR experiments with cDNA samples were normalized using the zinc-finger transcription factor *Pl*-*Z*12–1 mRNA as endogenous control, the expression of which remained relatively constant in all the developmental stages examined, according to Costa et al. (2012). Each assay included a no-template control for each primer pair. To capture intraassay variability, all real-time qPCR reactions were carried out in triplicate. Real-time qPCR amplifications were run in a ViiATM7 Real-Time PCR System (Applied Biosystems) thermal cycler, using a specific thermal profile consisting in four steps. The first cycle, for cDNA denaturation, was at 95 °C for 10 min. Second step was characterized by 40 cycles for amplification at 95 °C for 15 s and 60 °C for 1 min. The following step was for final elongation at 72 °C for 5 min. The last cycle, for melting curve analysis (from 60 to 95 °C), was set to verify the presence of a single product. Fluorescence was measured using ViiATM7 Software (Applied Biosystems). The expression of each gene was analyzed and internally normalized against *Pl*-*Z*12–1 endogenous control using REST software (Relative Expression Software Tool) based on the Pfaffl method (Pfaffl 2001; Pfaffl et al. 2002). The highest expression values for each gene at three developmental stages are considered 100% of fold expression, whereas the lower expression values of each gene are reported as percentage with respect to the maximum value.

## Results

### Apoptotic Genes in the Sea Urchin *P. lividus* Genome

The presence of factors with a key role in cell death signaling pathways, such as autophagy, extrinsic and intrinsic apoptosis, in the *P. lividus* genome was analyzed through a bioinformatics approach. Amino acid sequences of death factors, such as TNFRs, FAS, TRADD, AIFM1, BCL2, BAX, PINK, BID, PARP, and members of RIPK and ULK families, from *H. sapiens*, *S. purpuratus*, and other deuterostomes were used as query for the TBlastN and BlastP analysis. Casp3, Casp8, and NfkB were already identified in previous studies in *P. lividus* (Russo et al. 2014; Ruocco et al. 2016). *Paracentrotuslividus* genes studied are listed in table 1.

**Table 1:** Panel of Death Genes Investigated

Gene		Roles in *Homo sapiens*
**Extrinsic apoptosis**		
Pl_Tnfr16	Tumor necrosis factor receptor 16	It is a transmembrane receptor that plays a key role for the intracellular induction of cell death. It binds death ligands with the activation of death intracellular domains
Pl_Tnfr19/27	Tumor necrosis factor receptor 19/27	It is a transmembrane receptor that plays a key role for the intracellular induction of cell death. It binds death ligands with the activation of death intracellular domains
Pl_Ripk	Receptor-interacting serine/threonine kinase 4	It is an intracellular factor of death signaling pathway activated by the binding of ligands to death domain receptors
Pl_NfkB	Nuclear factor kappa B	It is involved in several intracellular mechanisms; it has central role in the apoptotic pathways through death receptors
Pl_Casp8	Caspase 8	It is involved in the programed cell death induced by Fas and various apoptotic stimuli; it interacts with Fas-interacting protein FADD
Pl_Casp3	Caspase 3	It plays a central role in the execution phase of apoptotic pathway; it is activated by caspase 8 and in turn activates caspases 6, 7, and 9
**Intrinsic apoptosis**		
Pl_Casp3	Caspase 3	It plays a central role in the execution phase of apoptotic pathway; it inactivates PARP and activates caspases 6, 7, and 9
Pl_Bcl2	B-cell CLL/lymphoma 2	It suppresses apoptosis regulating the mitochondrial membrane permeability, prevents the release of cytochrome c and binds APAF-1
Pl_Bax	BCL2-associated X protein	It binds and antagonizes the proapoptosis repressor BCL2; it undergoes a conformation change that causes translocation to the mitochondria, leading to the release of cytochrome c
Pl_Aifm1	Apoptosis-inducing factor, mitochondria associated 1	It is released from the mitochondria into the cytosol and to the nucleus, where it functions as a proapoptotic factor in a caspase-independent pathway. AIFsol is the soluble active form that induces fragmentation of DNA
Pl_Parp	Poly ADP-ribose polymerase 1	It is implicated in the induction of apoptosis and necrosis by DNA damage
**Autophagy**		
Pl_Ulk1/2	Unc-51 like autophagy activating kinase 1/2	It is a kinase involved in autophagy in response to starvation and stress conditions, regulating the formation of autophagophores
Pl_Ulk3	Unc-51 like autophagy activating kinase 3	It is a kinase involved in autophagy in response to cellular senescence, regulating the formation of autophagophores
Pl_Pink	PTEN-induced putative kinase 1	It is involved in the clearance of damaged mitochondria via selective autophagy (mitophagy), inducing activation of Parkin

**Fig. 1.**
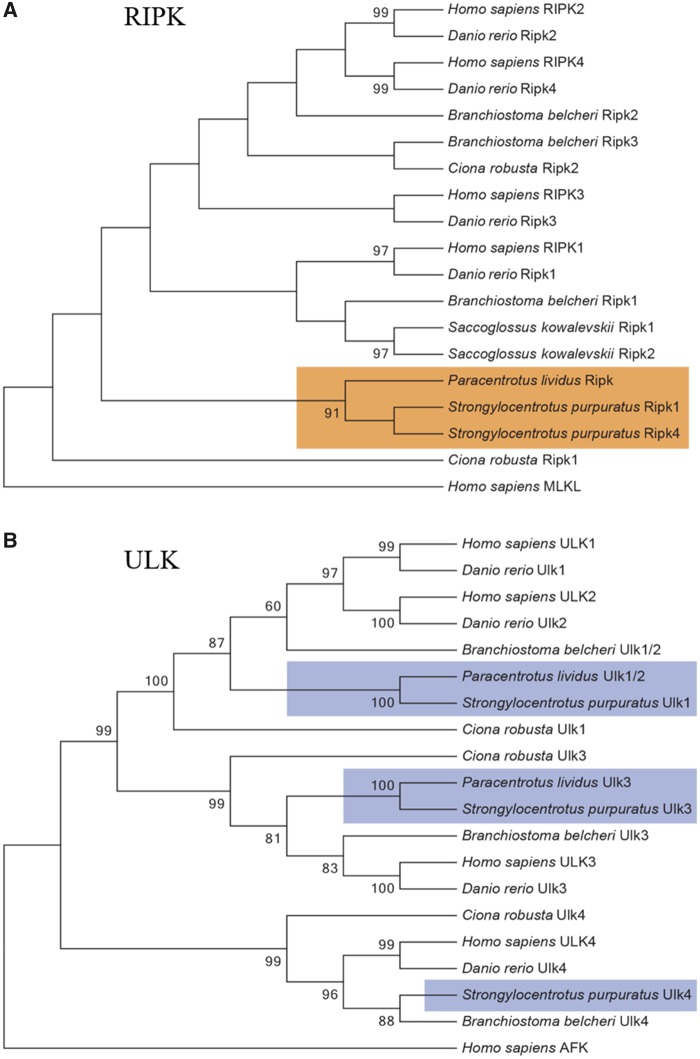
—Phylogenetic analysis of (*A*) the Receptor-Interacting Serine/Threonine Kinases (RIPKs) in *Paracentrotus lividus, Homo sapiens, Strongylocentrotus purpuratus*, *Danio rerio*, *Branchiostoma belcheri*, *Saccoglossus kowalevskii*, and *Ciona robusta*. The human protein MLKL was used as outgroup. The orange square highlights echinoderms genes. Phylogenetic analysis of (*B*) the Unc-51 Like Autophagy Activating Kinases (ULKs) in *Paracentrotus lividus, Homo sapiens, Strongylocentrotus purpuratus*, *Danio rerio*, *Branchiostoma belcheri*, and *Ciona robusta*. The human protein AFK was used as outgroup. The violet square highlights echinoderms genes. The numbers at the nodes indicate replicates and values for unsupported branches (<60) were removed.

The orthology of Aifm1, Bax, Bcl2, Parp, and Pink, present as single-copy genes in *P. lividus*, was confirmed by dedicated phylogenetic trees (see supplementary information IV, Supplementary Material online). In the case of Ripk, *P. lividus* database contains a single gene (*Ripk*), whereas two genes are present in *S. purpuratus*. The phylogenetic analysis confirms the orthology of the *Ripk* gene found (fig. 1*A*), although we cannot exclude that *P. lividus* lost a second *Ripk* gene or that it is indeed present in the genome but not identified yet in the current assembly. The same approach was applied for members of ULK family. *Paracentrotuslividus* genome seems to code for only two members of the ULK family. As shown in figure 1*B*, Ulk1/2 is ortholog of *H. sapiens* ULK1 and ULK2 and of *S. purpuratus* Ulk1, and Ulk3 of the single *H. sapiens* ULK3. The Ulk4 member, although present in all other deuterostomes analyzed, was not found in *P. lividus* genome (fig. 1*B*).

**Fig. 2.**
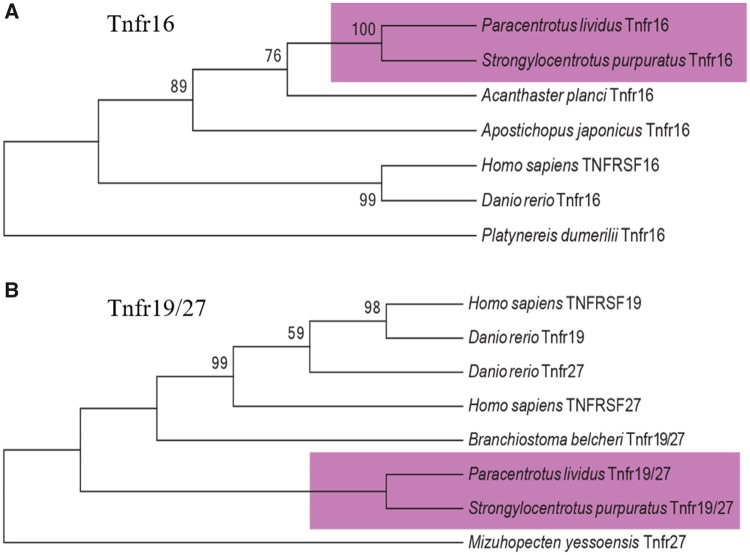
—Phylogenetic analysis of (*A*) the Tumor Necrosis Factor Receptors 16 (Tnfr16) in *Paracentrotus lividus, Homo sapiens, Strongylocentrotus purpuratus*, *Danio rerio*, *Acanthaster planci*, and *Apostichopus japonicas*. The *Platynereis dumerilii* Tnfr16 protein was used as outgroup. Phylogenetic analysis of (*B*) the Tumor Necrosis Factor Receptors 19/27 (Tnfr19/27) in *Paracentrotus lividus, Homo sapiens, Strongylocentrotus purpuratus*, *Danio rerio*, and *Branchiostoma belcheri*. The *Mizuhopecten yessoensis* Tnfr27 protein was used as outgroup. The violet squares highlight echinoderms genes. The numbers at the nodes indicate replicates and values for unsupported branches (<60) were removed.

In *P. lividus* genome, we found two sequences that most likely code for tumor necrosis factor receptor (Tnfr16 and Tnfr19/27). These two genes showed unequivocal phylogenetic relationship with *S. purpuratus* and *H. sapiens* members of the TNFR superfamily. For this reason, phylogenetic analysis conferred the specific annotations to Tnfr16 (ortholog of human TNFR16) and to Tnf19/27 (ortholog of *H. sapiens* TNFR16 and TNFR27) (fig. 2*A* and *B*). Additional members of the family could exist in *P. lividus*, nevertheless were not considered here for their uncertain orthology.

We did not find orthologs of human FAS, TRADD, and BID death factor in the *P. lividus* genome, suggesting that they could have been lost in this species (see supplementary information V, Supplementary Material online).

### Gene Expression Profile in Embryogenesis

Real-time qPCRs were performed in order to study the variation of expression levels of genes identified, during three developmental stages of the sea urchin *P. lividus* such as blastula, gastrula, and pluteus (5, 21, and 48 h post fertilization, hpf, respectively). In order to confirm cell pathway activation, we also studied three other genes, such as NfkB, Casp3, and Casp8, that are key actors in the downstream cascade of these pathways and that are already reported in the literature (Russo et al. 2014; Ruocco et al. 2016).

**Fig. 3.**
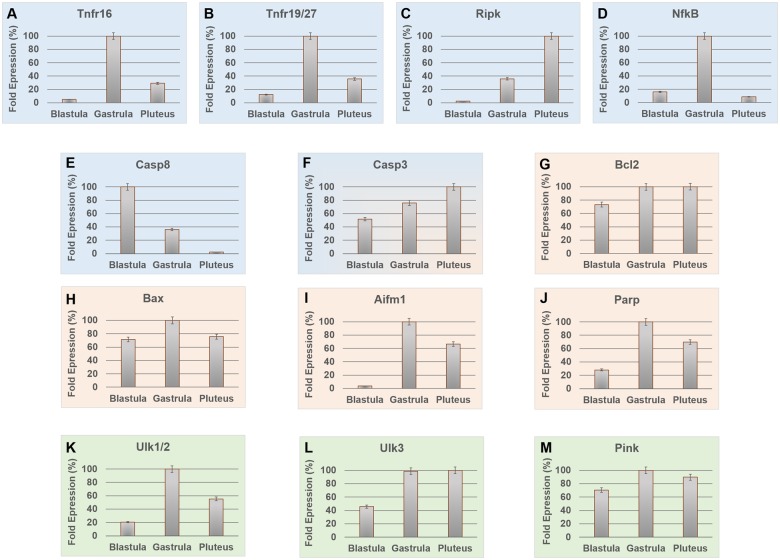
—Variation of gene expression levels of 13 genes during three stages of *Paracentrotus lividus* embryonic development (blastula = 5 hpf; gastrula = 21 hpf; pluteus = 48 hpf) by real-time qPCR. All graphs show the expression level (fold expression, in percentage) of each gene at specific developmental stages. 100% represents the lowest DeltaCt found for each gene, indicating the developmental stage with highest expression of the specific gene. Graphs with blue background (•) represent expression levels of genes involved in the extrinsic apoptotic pathway; with orange background (•) genes involved in the intrinsic apoptotic pathway, and with green background (•) genes involved in autophagy. *Paracentrotus lividus* Casp3 (blue and orange) is involved in both apoptotic mechanisms (extrinsic and intrinsic).

Tnfr16 was found highly expressed at 21 hpf (100%), whereas it showed lower gene expression levels at the other two developmental stages (5% at 5 hpf and 29% at 48 hpf) (fig. 3*A*). The same result was observed for the other TNFR. In particular, Tnfr19/27 showed maximum expression at 21 hpf and reduction at the other two developmental stages (12% at 5 hpf and 36% at 48 hpf) (fig. 3*B*). Ripk showed a linear increase with the progression of developmental stages (fig. 3*C*). In particular, gene expression level was 3% at the 5 hpf stage, 36% at 21 hpf, and 100% at the pluteus stage (48 hpf). NfkB showed 100% expression at 21 hpf and very low expression at 5 hpf and 48 hpf (16% and 9%, respectively) (fig. 3*D*). Casp8 and Casp3 showed an opposite expression pattern during embryonic development (fig. 3*E* and *F*, respectively). In particular, Casp3 expression level increased during the progression of the three stages (52% at 5 hpf, 76% at 21 hpf, and 100% at 48 hpf), whereas Casp8 expression level decreased from earlier stages to larva (100% at 5 hpf, 36% at 21 hpf, and 2% at 48 hpf). Bcl2 was highly expressed in the late developmental stages (21 and 48 hpf, 99% and 100%, respectively), whereas it showed a slight reduction in expression to 73% during the first stage (5 hpf) (fig. 3*G*). Bax exhibited the highest expression level at 21 hpf (fig. 3*H*) with an analogous low level of expression (about 25%) at 5 and 48 hpf. Aifm1 gene expression was very low at 5 hpf (4%) (fig. 3*I*) but at 21 hpf the expression level was highest (100%), decreasing to about 65% during the larval stage (48 hpf). Parp gene exhibited highest expression level at the intermediate stage (21 hpf, 100%), with 28% of fold expression at the 5 hpf and 70% at the larval stage (48 hpf) (fig. 3*J*). Also Ulk1/2 was highly expressed at the intermediate developmental stage (21 hpf), whereas the expression percentages were lower at the other stages (20% at 5 hpf and 55% at 48 hpf) (fig. 3*K*). Ulk3 showed high expression levels at 21 and 48 hpf (99% and 100%, respectively) but decreased during the first developmental stage (45%) (fig. 3*L*). Pink gene showed a different expression pattern and was highly expressed (expression level >70%) for all developmental stages studied (71% at 5 hpf, 90% at 48 hpf, and 100% at 21 hpf) (fig. 3*M*).

## Discussion

**Fig. 4.**
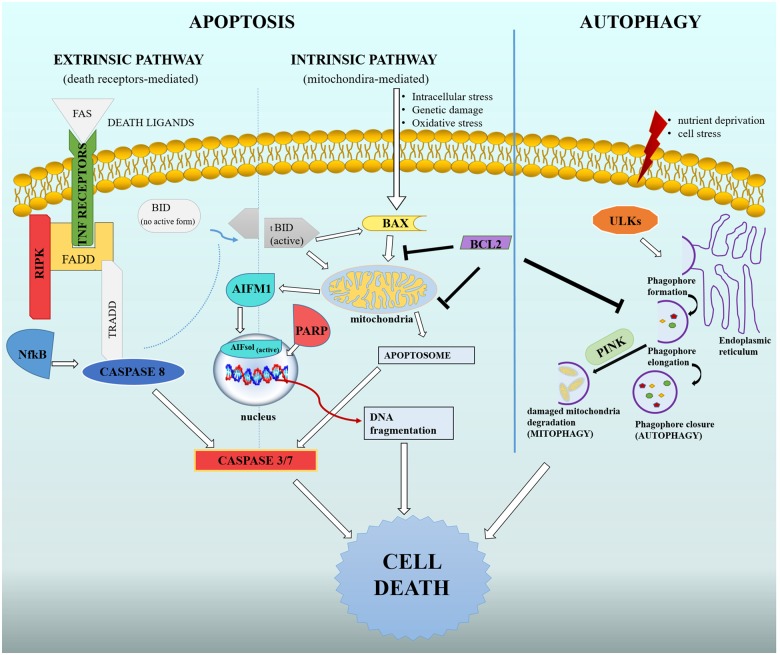
—Schematic representation of autophagy, extrinsic and intrinsic apoptosis in the sea urchin *Paracentrotus lividus*. All genes found are represented with a specific color, whereas those in white were not found using bioinformatic analysis.

The results indicate the presence of several key factors involved in different cell death pathways in the sea urchin *P. lividus* genome. In this nonvertebrate deuterostome, extrinsic and intrinsic apoptosis seem to be conserved as two separate death mechanisms and the two pathways are activated in different stages of its embryonic development. Moreover, autophagic factors are conserved in this sea urchin species and gene expression analysis revealed activation of an autophagic pathway in embryos, suggesting its fundamental role during development (fig. 4).

TNFR superfamily is involved in extrinsic apoptosis. In *P. lividus* genome some TNFRs were found, but this study focused on two receptors that showed higher phylogenetic support, such as Tnfr16 and Tnfr19/27. Differently from *H. sapiens* and vertebrates in general, genomes of nonvertebrates animals do not encode for a large number of TNFRs (Wiens and Glenney 2011). This is confirmed also in the sea urchin *S. purpuratus* genome, which contains only three TNFRs with death domain (Robertson et al. 2006). The two genes identified in *P. lividus* showed homology with deuterostome genes (in particular one with members 16, and the other with 19 and 27, as annotated in the gene names). TNFR16 in *H. sapiens* plays a role in the regulation of cell survival as well as cell death of neural cells starting at very early developmental stages (Twohig et al. 2001). Also, TNFR19 is highly expressed in humans during embryonic development and is known to induce apoptosis by a caspase-independent mechanism (Eby et al. 2000). Tnfr16 and Tnfr19/27, known to be involved in the same death mechanism, showed a similar expression level profile in *P. lividus*. They exhibited low expression levels at the blastula stage, with the highest expression at the gastrula stage and a reduction of more than 50% at the larval stage.

*Ripk* seems to be present in the *P. lividus* genome as a single-copy gene with a high homology with members of the deuterostome serine/threonine kinases family, although it cannot be excluded the presence of other *Ripk* genes. *Ripk* encodes for a downstream factor associated with intracellular death domains containing receptors (e.g., TNFRs), with a key role in the signaling transduction to executioner machinery (fig. 4). The expression profile of *Ripk* showed a stage-dependent activation of the gene, with a peak in expression at the pluteus stage. This result could be temporally related to receptor activation at the gastrula stage (*Tnfr16* and *Tnfr19*/27), able to promote an increment in gene expression of intracellular downstream death factors. This result could indicate that extrinsic apoptosis may be involved in cell death prevalently after gastrulation, mainly during the larval stage.

*NfkB* was upregulated at the gastrula stage; the expression pattern of the gene was similar to death receptors *Tnfr16* and *Tnfr19*/27. This is not surprising because TNF and NfkB signaling are tightly related in vertebrates (Chan and Aggarwal 1994; Gupta et al. 2005). In our study, NfkB level seems to be regulated at the same time as TNFRs.

Some of the main factors involved in the intrinsic pathway of apoptosis have been investigated. In particular, Bax (proapoptotic factor) and Bcl2 (antiapoptotic factor) are both conserved in the *P. lividus* genome. Moreover, they were found to be highly expressed during all developmental stages. This result is consistent with the key role of Bax and Bcl2 in the intrinsic apoptotic process, because they belong to the Bcl2 family, a group of pro- and antiapoptotic proteins that control cell fate and that are fundamental in remodeling processes, characteristic of embryogenesis in many organisms (Boumela et al. 2011).

Caspases are executioners of apoptosis. In particular, Casp3 is activated in both extrinsic and intrinsic apoptosis (Salvesen 2002; Ghavami et al. 2009), whereas Casp8 is involved in the signal transduction of death domains (extrinsic pathway). In our study, Casp3 proportionally increased in a time-dependent manner, reaching its highest expression level after 48 h. This result is coherent with the role of this factor and its temporal position along the apoptotic cascade, being a downstream molecule at the end of the cascade. The same result was observed in other studies, where Casp3 was described as a key molecule of apoptotic processes activated during *P. lividus* embryonic development (Roccheri et al. 1997; Agnello et al. 2006; Agnello and Roccheri 2010). On the other hand, *Casp8* showed a peak of expression in the earlier developmental stage examined, with a time-dependent decrease in expression levels in later stages. Our results indicate the involvement of two specific apoptotic mechanisms in early (via Casp8, probably with maternal mRNA influence) and late (via Casp3) development (Sakata et al. 2007). The fundamental role of these two caspases is confirmed by a previous study, which provided evidence of Casp3 and Casp8 activation in *P. lividus* embryos exposed to apoptogenic compounds (Ruocco et al. 2016).

Nucleic acids are one of the main intracellular targets during apoptosis. In vertebrates, two factors, AIFM and PARP, are involved in the DNA fragmentation process leading to cell death (Kim et al. 2006; Hong et al. 2013). We have identified Aifm1 and Parp as clear orthologs of deuterostome genes. They both had low expression levels (< of 30%) at the blastula stage, followed by maximum expression during gastrulation. At the pluteus stage, Aifm1 and Parp remained upregulated, decreasing their expression levels of about 30%. These two genes showed the same expression profile, coherently with their common localization (nucleus), same intracellular target (DNA) and involvement in the same phase of the apoptotic process (DNA fragmentation) (Liu et al. 2018).

Another finding of this study regards the identification of key genes involved in autophagy and mitophagy in the *P. lividus* genome. In particular, two Ulk genes (Ulk1/2 and Ulk3) were found in the *P. lividus*, which showed a substantially similar expression pattern, coherently with their involvement at the same stage of the autophagic pathway. Ulk genes encode for proteins responsible for the formation of autophagosomes, the execution step in autophagy that is required for the subsequent activation of Beclin1 (Joachim et al. 2015) (table 1 and fig. 4). During *P. lividus* embryonic development, both *Ulks* genes showed a low expression at the blastula stage (<50%) with highest transcript levels during gastrulation. The expression decreased after the gastrula stage, remaining at 50% at the pluteus stage. These comparable expression profiles suggest the involvement of these two factors in the execution phase of the autophagic signal transduction.

Pink gene encodes for a protein responsible for the activation of a specific autophagic pathway: mitophagy. *Paracentrotuslividus* genome contains a single-copy gene for Pink, and data on its expression profile showed a high transcript level during all developmental stages. In particular, at the blastula stage the expression was already over 50%, reaching maximum value during gastrulation and remaining high (>80%) at the larval stage. Elimination of maternal mitochondria is an essential process during embryonic development (Zhou et al. 2011; Rojansky et al. 2016). The expression profile of Pink may be related to the activation of mitochondrial clearance during *P. lividus* embryonic development.

Our study also highlights the simultaneous involvement of apoptotic and autophagic genes, confirming results described by Agnello et al. (2016) who found a temporal connection between induction of autophagy and apoptosis activation, which suggests that they are two functionally and temporally related mechanisms. This relationship has already been described in *P. lividus*, because the inhibition of autophagic mechanism produces a subsequent reduction in apoptosis (Agnello et al. 2016).

Orthologs of death genes FAS, TRADD, and BID were not found in the *P. lividus* genome (supplementary information V, Supplementary Material online). In any case, we cannot exclude that this could be due the low coverage of the genome database consulted. This result is confirmed by a previous investigation, where these death factors were also not found in the phylogenetically related sea urchin *S. purpuratus* genome (Robertson et al. 2006).

## Conclusions

Results emerging from this investigation are in accordance with previous studies conducted on other sea urchin species, mainly on the purple sea urchin *S. purpuratus*. Vega Thurber and Epel (2007) demonstrated biochemical activation of caspase-mediated apoptosis that represents the predominant death signaling pathway during blastula and gastrula stages of *S. purpuratus* development.

During animal gastrulation apoptotic processes are fundamental for cavity formation by the removal of inner ectodermal cells (Agnello et al. 2015). The expression profile of most of death genes here described corroborates in sea urchins the importance of ontogenic cell death mechanisms, prevalently at gastrula stage.

The fully sequenced and annotated genome of the purple sea urchin, *S. purpuratus* (Robertson et al. 2006), has promoted the use of this model organism for a wide variety of experimental applications in molecular biology, ecotoxicology, and evolution fields. However, this is the first study that not only identifies cell death genes at the bioinformatics level in the *P. lividus* genome but also describes their variations in expression levels in different developmental stages. Our results suggest an involvement of death mechanisms during *P. lividus* ontogenesis, but further studies are still necessary to demonstrate the functional role of death genes identified. Progress in sea urchin genome sequencing will shed further light also on the strong correlation between cell death mechanisms and elucidate what parts of the vertebrate apoptotic toolkit are also present in nonvertebrate deuterostomes.

In the future, it would be interesting to study spatial expression pattern of death genes and to compare the activation of death pathways during development in normal and stress conditions. Moreover, it would be fundamental to study whether specific proapoptotic stimuli are able to induce the same death pathway in different model organisms, pointing to a functional conservation.

## Ethics Approval

All animals were collected in the Gulf of Naples, from nonprotected or private areas, according to Italian legislation of the Marina Mercantile (Decreto del Presidente della Repubblica DPR 1639/68, September 19, 1980 confirmed on January 10, 2000). The study involves only sea urchins *Paracentrotus lividus*, not any protected or endangered species. All animal procedures were in compliance with the guidelines of the European Union (Directive 609/86), on the protection of animals used for scientific purposes.

## Supplementary Material

Supplementary data are available at *Genome Biology and Evolution* online.

## Supplementary Material

Supplementary Data
