# Supplementary material for: Identification of Cell Death Genes in Sea Urchin Paracentrotus lividus and Their Expression Patterns during Embryonic Development
Source: Genome Biol Evol. 2019 Jan 29;11(2):586–96. doi: 10.1093/gbe/evz020 (PMC6394757; doi:10.1093/gbe/evz020)
Supplement: Supplementary Data [file evz020_supp.zip › Supplementary Information I.pdf]

Supplementary Information I

SOURCE:
Gene prediction from *Paracentrotus lividus* genome (v3.0, Genoscope, Sept 2014) from BioDev (www.octopus.obs-vlfr.fr) using Genescan on reported contigs or scaffolds.

We list here contigs and scaffolds accession numbers, CDS prediction and protein prediction respectively, for each gene studied.

Aifm1

>lcl|Contig19680

>Pl_Aifm1
TASFAALRAIRANDPKAKVLVVGDEDATPYMRPPLSKELWFSDDKEGVENLKFKQWNGKEKSIFFE
PDAFYCKPSELSTKENGGVAVLKGHKVKGLNVQKKQATLADGSVITFDKCLIATGGVPRNLGSIEK
AGSEVIDRTTLFRNIKDFKSLDKATEDAKSVAIIGGGFLGSELACALGKRGKQTGMKVMQFFPEAG
NMGRVLPEYLSKWTTDKVSNEGVEVYPDMLLKNVSFNDESKQVELQFKNGEKVQADHVVVAVGLEP
NTDLAASSGLEVDDKFGGYRVNAELEARRDVWVAGDASCFYDIKLGRRRVEHHDHAVVSGRLAGEN
MTGAAKPYWHQSMFWSDLGPDVGYEAIGIVDSSLPTFGVFAKATKEDTPKAVVEATGEGIRSETEQ
AAVETTPAPEAPPLSDDYGKGIVFYMKDKVVVGMVLWNIFNKMPIARKVIKDGKEYDDVSELAKLF
NLHKTEES

>Pl_Aifm1
nggactgcatcgtttgccgccctcagagctatccgagccaacgaccctaaagcaaaggttctggtt
gttggggatgaagacgccacaccatacatgagaccacctctatccaaagaactctggttcagtgac
gacaaggaaggagttgaaaacctcaagttcaagcagtggaacggcaaggaaaagagcattttcttt
gagccagatgctttctactgcaagccatctgaactatcaacaaaggagaatggaggagttgcagtg
ctcaaaggtcacaaggtcaaaggtctgaatgtacagaagaagcaagctactttggcagatggatcg
gtcatcacttttgataagtgccttattgcaactggtggtgttccaaggaatctagggtcaatagaa
aaggctgggtctgaggtcatcgacagaacaacacttttcagaaatatcaaggacttcaaatcttta
gataaagcgacagaggatgccaaatcagtggctataattggaggggggattcctgggcagtgagctg
gcatgtgctcttggcaagaggggtaaacagactggaatgaaagtcatgcagttcttcccagaagca
ggtaacatgggcagagttctcccagagtacctcagcaaatggaccacagataaagtatcaaatgag
ggtgttgaagtttatcctgacatgctactgaagaatgtttccttcaatgatgaatcaaaacaagta
gaacttcagttcaagaatggggaaaaggtacaagctgaccatgtagtagtggcagtgggtctggaa
cccaacactgaccttgcagcaagttcagggttagaggtcgatgataagttcggaggttacagagtc
aatgctgagcttgaagctcgtagagacgtttgggtggctggtgatgcatcatgtttctacgacatc
aagctagggcgtcgccgtgtagaacatcacgatcatgctgtagtgagtggcagactagcaggagag
aacatgacgggagctgctaaaccatactggcatcaatcgatgttctggagtgatttaggtccagac
gttggatatgaagccatcggtatcgttgattccagtctaccaacctttggtgtgtttgctaaggct
acaaaagaagatacacccaaggctgtggttgaagctacaggagaaggtatccgctctgaaacagaa
caggctgcagtagaaaccaccccagcacccgaagctccacctctctcagacgattatggaaagggt

attgtattctacatgaaggacaaggttgtagtaggaatggtcttatggaacatcttcaacaagatg
cccattgcaagaaaggtgatcaaagatggtaaggaatacgatgacgtcagcgaactagccaagctt
ttcaacctccataaaacagaagaaagctga

Bax
>lcl|ATILLocus_3927_Transcript_8/8_Confidence_0.680_Length_2685

>Pl_Bax
MAEGGSDTPCDETDASTRLRIEQTRFERQVSRDDVGEQATVLLQHFIVERFQQDGFENAPGLDELRAGHAVSS
EQEVVWSEVGSMLRSIGDELDRDQELQRMINSIPADSPIEAIIAVAHVVFLDGDISWGRIVGLFYFAYRMAAR
AIDSVLEKSFPNWINKLIKEVVKFLVLKFAHWIISKGGWLAIREYMGSPTWIWGTL

>Pl_Bax
atggctgaaggggggctcggatactccttgtgatgaaactgatgcatcaacccgtttgcgtatcgagcagacac
ggtttgagcgtcaggtgagccgcgatgatgttggggagcaggccactgttctccttcaacatttcattgtgga
gcgtttccagcaggacggctttgagaatgccccaggtttagatgaactgagggcggggcatgctgtgtcgagt
gagcaggaggtcgtctggtcagaggtcgggtcaatgctgaggtcaatcggggacgagttggacagagatcagg
agctgcagagaatgataaactccataccagccgactctccaatcgaggctatcatagcagtggcccatgtggt
ctttcttgatggtgatatctcatggggcaggatagtaggtctattctacttcgcttacagaatggctgccagg
gcaatagactctgtattggagaagagctttccaaactggatcaacaaactgatcaaagaagtcgtcaagtttc
tcgtcctcaagtttgctcactggatcatcagcaagggtggatggctggccatccgagaatacatgggctcacc
cacatggatatggggcactctg

Bcl2
>lcl|scaffold12593

>Pl_Bcl2
MPDISTASIVADYLRYRLQCLGNGVDAEENDCLSTVMLVTEAVSSLHEAIVNLSEKLMNDYRDSFS
QAYNQVLAHGDGSMNYGIFKAGMKSMVKDTVPSWGRIVAFFSFGGALCVWCVEKDIRAFPTVEELM
VAYINSHLITWIHANGGWTNVDLDKIDSETWSEISSRNPSPQGAQSPSHTTPP

>Pl_Bcl2
atgccggatattagcaccgcgagcattgtggcggattatctgcgctatcgcctgcagtgcctgggc
aacggcgtggatgcggaagaaaacgattgcctgagcaccgtgatgctggtgaccgaagcggtgagc
agcctgcatgaagcgattgtgaacctgagcgaaaaactgatgaacgattatcgcgatagctttagc
caggcgtataaccaggtgctggcgcatggcgatggcagcatgaactatggcatttttaaagcgggc
atgaaaagcatggtgaaagataccgtgccgagctggggccgcattgtggcgtttttttagctttggc
ggcgcgctgtgcgtgtggtgcgtggaaaaagatattcgcgcgtttccgaccgtggaagaactgatg
gtggcgtatattaacagccatctgattacctggattcatgcgaacggcggctggaccaacgtggat
ctggataaaattgatagcgaaacctggagcgaaattagcagccgcaacccgagcccgcagggcgcg
cagagcccgagccataccaccccgccg

Parp
>lcl|scaffold06675

>Pl_Parp
MSPSAGLIFEGPHPIGKLLTSAAPPTSTLLYLVAFITRAFSEPAKSKSMSLLHLNRCQRHTSEKDT
RLNLYLVYTVTRVAGLSCHVYKEKGLLYSATLGLVDISRGTNSYYKLQLVQHDTQSRYWVYRSWGR
VGTTIGGNKKDNFGSDVKKAKEHFRAVYLEKTGNGFGAKNPIKRPMKFFPLDISYGEEEDRIISSK
ERAGKTSKLPKEVQSLMKMIFDLEELKKTMLEFEIDLEKMPLGKLSKKQIEDAYKVLTDLQKPFWL
CLIRLHAAVHQVCECGEEETNKTQMLDSLLDIEIAYSMLKETGEEGVDPVDVHYEKLKCPMEVVDK
KSDEFKMISDYTTNTHAATHNWYRLTVEEVFRIDREGEGSRFKPFKKLHNRQLLWHGSRKTNFGGI
LSQGLRIAPPEAPATGYMFGKGIYFADMVTKSANYCYANASSNIGLMLLTDVALGDMYELRGAKGM
SKPPAGKHSTKGLGRTCPDPSGLRTIEDNLQVPMGFDSTDKSYKTNLPIVLGAVPQNHKSILGQAI
GTRVVELTHVSMGCPKTYDGFVVRPPGEVRYRPEIYVNSKSHRRRISIKRSPHTSCAG

>Pl_Parp
atgagcccttctgctgggttaatatttgaagggcctcatcccattggtaagctcctaactagtgca
gcaccgcctacatcgactcttttgtatcttgttgcttttattaccagagctttttcagagcctgcc
aaatctaaatcaatgtctttgctgcatctcaaccgatgccaaagacacaccagtgagaaagacaca
agattgaacttatacctcgtttacactgtgactcgggtcgcaggtttgtcatgccatgtatacaag
gagaaaggattgttatacagtgcaacattgggcctggttgacatcagccgtggaactaactcctac
tacaaactacagcttgtccaacatgacacgcaatctagatattgggtgtaccgctcttggggacga
gttggcaccactattgggggcaacaagaaagacaactttggctctgatgtgaagaaggccaaggag
catttcagagctgtttaccttgagaaaaccggtaacgggtttggagccaagaacccaatcaaacgc
cccatgaagttctttcctctggatatcagctatggagaggaagaggataggataatatctagtaaa
gaacgtgcaggaaagacgtccaagctacccaaagaggtccaaagtttgatgaaaatgatatttgac
ttggaggagctcaagaaaacgatgctggaatttgagattgatctggaaaagatgcctttaggaaag
ctgtccaagaaacagattgaagatgcttacaaagtactcacagacctacagaagcctttctggctg
tgccttattcgcctgcatgctgctgttcaccaagtttgtgaatgtggagaggaagagaccaacaag
acccagatgctggacagtcttctagatattgagattgcctatagcatgctgaaagaaactggagag
gaaggtgtcgacccggttgatgttcattatgaaaaactcaagtgcccaatggaggtggttgataag
aagagtgatgagtttaagatgatcagcgattacacgacaaacacccatgctgccacgcacaactgg
tatcgccttaccgtggaagaggtcttcaggatagacagagagggtgaaggatcaaggttcaagcct
ttcaaaaaactccacaacagacagctgttatggcatggatcccgtaaaccaactttggaggaatc
ttatcacaggggcttaggattgcacctcctgaagcaccagcgactggctatatgtttggcaagggt
atctactttgctgacatggttacaaagagcgccaactactgttacgcaaatgccagcagtaacatt
ggtctcatgcttcttacagatgtcgcacttggtgatatgtatgagctgagaggagcaaagggcatg
tctaaaccacctgcaggcaaacacagcactaaaggtcttggacgtacatgtccagatccgtcaggt
ttgagaaccatcgaggataatctacaggttcctatggggtttgattcaactgacaaaagttataag
accaatttgcctatagttctgggggctgtaccacaaaaccataagtctattcttggacaagccata
gggacacgtgttgtagaattaacacacgtttcaatgggttgtccaaaaacttatgacggttttgtg
gtacggcccccaggagaagtaaggtatcgtccggaaatttatgtaaattcgaagtcacatcgccgt
cggatttcgataaagcgtagcccgcacacttcgtgtgcgggctaa

Pink

>lcl|scaffold08146

>Pl_Pink
MSFRHGLQAIARVVRRRLQQQAQQHTHEHHAGRQRPDVATSNPAQRYHSSRPTGSDVRLNTQNLAI
RAARVWRQTPSSWSSNGSRFVQSSRPVLPLLLGFAGIGLANQDGEGFAARGGIDLETALQVVQTVF
DDRGLKAGQDTSKEFPDNLASYAFSKTVLAKGTEGAVFAAKRRDTVLPSKGDEEFADAQARYMPVD
QPIDGGHIGEQPKAEMKVCDEGECNLAIKMMFNYTGTGGSKPADIMAEFGAEQLPLVLAGGSFSGR
SNKYRRYRTSLRDYLNKGSGLPDRSIMVIVAQLLEAVGYLGNQGVVHRDLKSNNILVDYEEGSDEV
PHVVVADFGCAISLRGKNLQETVSKDDLNRQGNAALMAPEVKKAYHCNSLRYHDYLKADVWAVGAI
MYEVCGKKNPFYAEGIDSSKYEAGDLPQLQSEAVGLRVVSELLLEKNPGNRPSAQVAANILHLLLW
QPSVSSFLRLSQGQSSKDSELTAWIIKVSLMQQLLSSNHTDTALTFTPDDPGLPQNVIESSLVETF
LKRVNSDSLIDASQRLVTALRPLGALAEFRGVSSTCMGTPQLIGSVSDTVTLMA

>Pl_Pink
atgtcgttccggcatggcttgcaagccatcgctcgagtggtgcgacgaaggctgcagcagcaggca
caacaacacacccacgaacatcatgcaggccgacagaggcccgatgtagcgacatcgaatcccgct
caacgctaccatagcagtagaccaactggatccgatgttcgtctaaatactcaaaatcttgcgatt
agggcggcacgggtttggaggcagactccttcatcatggtcatcaaatggtagtagatttgttcaa
tcatcacgtccggtattgccactcttactaggatttgcaggaattggacttgccaatcaggatgga
gaaggttttgcagcaagaggtggcattgatcttgagacagctctgcaggttgtacagactgtcttc
gatgaccggggcttgaaagcaggtcaggacacatctaaagagttccccgacaacctagctagttac
gcattctcaaaaactgtcctggcgaaaggcacagagggcgcagtctttgctgccaagaggagagac
acggttcttccatcaaagggggatgaagagtttgctgatgcccaagctaggtacatgcctgtggat
cagccaatagatggagggcatatcggagagcagccaaaggcagagatgaaagtttgtgatgaggga
gagtgcaatctagccattaagatgatgtttaattacactggcactggggggatccaaaccagctgac
atcatggcagaatttggggctgaacagctcccccctagtgttggctggagggtcattctctggtcga
tccaataaatataggaggtacagaacatccctcagggattatctcaacaaggggtctggccttcca
gatagaagtatcatggtcatcgttgcccagttgctggaagcagttggttaccttggcaaccagggt
gtggtccatcgtgacctcaagagtaacaacatcctcgttgactacgaggaaggctctgatgaggta
ccccacgtggtggtcgcagactttggatgtgcgatttcattgcgaggtaaaaacctgcaggagact
gtgagcaaagatgacctgaacaggcaaggcaatgctgcccttatggcccctgaggttaagaaggca
tatcattgtaactctctgcgctaccatgactacctaaaggcagatgtctgggcggtgggagccatt
atgtatgaggtttgcggtaaaaaaaatccattctatgctgaaggaatcgacagcagcaagtatgaa
gctggtgaccttccccagctccaaagtgaagcagtgggactgagggtagtgtcagagctcctgctg
gagaaaaaccctggaaatcgaccatctgctcaagtagctgcaaacattcttcatctattactgtgg
caaccatcagtcagcagtttcctgcgtctgtcccagggtcagtcgtcgaaagatagcgagcttacc
gcctggattattaaggtatctctcatgcaacagctcttgtcatcgaaccataccgatacggcgcta
accttcacccccgacgaccctgggcttccccagaacgttatagagagcagtcttgtggagacgttc
ttgaagcgtgtcaattcagacagtctgatcgatgcctctcaacgcctcgtcacagctcttcgccca
ctcggagcgttggcggaattcagaggggtgtcgtccacgtgcatgggaacgccacagctgataggc
agtgtctctgacacagtgacacttatggcctaa

Ripk
>lcl|scaffold10601

>Pl_Ripk
MKYMERGSLWDFRIKKWKDIPDLRPLTNRMVYQISSGMHFLHSIYIIHRDLKLENVLIDAQLIVKI
ADLGLATNLKTSSGDNCWGTDSHKPPEAFRTDLPSKTKVVTTKYDVYSFSMTLYQLLTGIHPYSDR
GIDMIRLLKVEAKQTPSLQPIPDNTTPELIEILKASWRYEANERPNFKEITNRVGDLDTDPSYESL
AVYFGQPSSKVTKP

>Pl_Ripk
atgaaatatatggaacgcggcagcctgtgggattttcgcattaaaaaatggaaagatattccggat
ctgcgcccgctgaccaaccgcatggtgtatcagattagcagcggcatgcattttctgcatagcatt
tatattattcatcgcgatctgaaactggaaaacgtgctgattgatgcgcagctgattgtgaaaatt
gcggatctgggcctggcgaccaacctgaaaaccagcagcggcgataactgctggggcaccgatagc
cataaaccgccggaagcgtttcgcaccgatctgccgagcaaaaccaaagtggtgaccaccaaatat
gatgtgtatagctttagcatgaccctgtatcagctgctgaccggcattcatccgtatagcgatcgc
ggcattgatatgattcgcctgctgaaagtggaagcgaaacagaccccgagcctgcagccgattccg
gataacaccaccccggaactgattgaaattctgaaagcgagctggcgctatgaagcgaacgaacgc
ccgaactttaaagaaattaccaaccgcgtgggcgatctggataccgatccgagctatgaaagcctg
gcggtgtattttggccagccgagcagcaaagtgaccaaaccg

Tnfr16
>lcl|scaffold20496

>Pl_Tnfr16
MFCPPGFGAVVPCSPQQSSRCELCDNGTYSDLVSSTEGCKTCSVCKEGSIVLKRCTDISDTVCSDT
YLPPVTSQPANDDITTMVWIPRSGPTSSGFSVVPIFCTLLGLVIFGLLAYVIFKKWSFKKMKLRQT
QKNMTRSSSCHTDIEGNTISILSLKNGQSYYASRDSALDRSGAGSICRQPLMAVPSAMVYQQLPPD
KRYEVERALSASRMDGRDWRGLARELGFSDLDIVHIAQTCTGSTPPGRAMLITWHSRDPKRASVGT
LVEALRRIRRNDVADLIPVFTYSAYHFPTS

>Pl_Tnfr16
Atgacgtcagcctgcagcatcatgcaagacactgtgtgcgagtgcgcgccaaactatttccggacg
gacgcgcagccgagtggcacttcggcggtggggaccgcctcaacgtgcagccagtgtatgttctgt
cctcctgggttcggtgcggtagttccttgttcgcctcaacagagttctagatgcgaactgtgtgac
aatggaacctactcggatctcgtcagttcaacggaaggatgtaaaacatgttcagtctgcaaggag
ggtagcattgtactcaagagatgcactgatatctcagatacagtatgctcagatacctacctacca
ccagtgacttcccaaccagccaatgatgacatcacgacgatggtatggattccgaggtcgggcccg
acatcgtccggtttcagcgtcgtgcccatcttctgtaccctccttggactcgtcatctttggtctg
cttgcttacgtcatcttcaaaaaatggtccttcaagaaaatgaagctgaggcagacacagaagaac
atgaccaggtcgagttcgtgccataccgatattgaaggaaacacaatatcaatcctttcactgaag
aatggtcagagctattatgcatccagggattcagccctagatcgatcaggtgccggcagtatctgc
agacaacctcttatggcagttcccagtgcgatggtgtatcagcaactgcctccagacaaacgttac
gaggtggaacgcgccctctccgcttccaggatggatggccgtgactggcggggtctggcccgggaa
ctaggattctccgatctagatattgtccacatcgctcagacgtgtacaggctcaacgcctccaggt

agagcaatgctcatttcctggcacagcagagaccccaaacgcgcatctgtcgggacgctcgtcgaa
gcgctgcggaggatacgacgaaatgatgttgccgatttaataccagttttcacctatagtgcttat
cattttcctacttcatga

Tnfr19/27

>lcl|scaffold13431

>Pl_Tnfr19/27
MAEPKVSYDGLEDCSSASFLNTGLQRMVVAIVILSILSSVQDSVDALPTSSPSLNHKDVNLTRQSL
MMIDKSMPDDDIQDLSSSSDYESLPLTVQVADDCSSDQFLHPSGVCILVVTIWGIKVAFLYSITGS
SDYESLPLTVQVDKDCSSDKYLNQSGVYILVIASYIRQTICLLYYITGSSDYDSLPLTVQVAEDCS
SDQYLDQSGVCRNCTTCGPGTQHGVDKKCGYGNGFEQICEPCPDGYYQGGTRSDLVRCNRCITCDE
LAETVQECSSVNNTECGPCPAGKYYYYPNVCFPCEAGGDDKEECMATKPTTPAPTSSTSPPPIRTT
VNMTFFNQTESPGNTTEEPSPGGTNKIVTACVTVFVIAIPVSLVAICVYKFLKKRHSRSDEESTGE
KGGEENGSKSGDPGVISDQAQYNARTGAVDLVRYSPVPLQEPPENGTDLSLPQTLRSDLPFDPPPC
VCNGQSQVEPDQGLHALAPAVSIVGQDTPPSRR

>Pl_Tnfr19/27
atggccgagccgaaagtctcctacgatggattggaagactgttcctcggctagtttcctgaataca
ggccttcagcggatggttgtggcaattgtaatactatctatattgtcctctgttcaagattcagtg
gatgctcttcccacatcaagtccttccttgaatcacaaggatgtgaatctaaccagacaaagtctg
atgatgattgacaagagcatgcctgatgatgacatccaagatctttcaagttcctctgactacgag
tcactgccactgactgtccaggtagctgatgactgttcatctgatcagtttctacacccatctggt
gtttgcatattagtcgttactatatggggcataaaagtcgcattttatattctataacaggttcc
tcggactatgagtcactgccactgactgtccaggtagataaagactgttcatcggataagtatctc
aaccaatctggtgtttacatattagtcattgctagctatatcaggcaaacaatttgcttattatat
tatataacaggttcatcggactacgattcactgcccctgactgtccaggtagctgaagactgttca
tctgatcagtatctagaccagtctggtgtttgcaggaactgtacgacatgtggaccagggacgcag
catggtgttgataagaaatgtggttatggaaatgggttcgagcagatctgtgagccttgccctgat
ggatactaccaaggtggtacgaggtctgatctcgtacgatgtaatcgttgcatcacttgtgatgaa
ttggctgaaactgtacaggagtgttcttctgtgaacaatactgaatgtggcccatgtcctgctggc
aagtactactattacccaaacgtatgctttccttgtgaggctggggagatgacaaagaagaatgc
atggcaacaaaacccaccactccagctccaacctctagcacttcaccacctcccatccggacaact
gtgaatatgactttttttaaccagactgaaagtcctggtaatacaactgaagagccttctccaggt
gggacaaataagattgttactgcatgtgtgactgtgtttgtcatcgctattccagtttcccttgtt
gcaatttgtgtatacaagttcctcaagaagcgtcatagtagaagtgatgaggaatctactggagaa
aagggcggtgaagagaatggatcaaagagtggagaccctggtgtgatatctgatcaagctcagtac
aacgctcgaactggagctgtcgacctggtaagatacagtcccgtcccccctgcaagaaccacctgaa
aatgggaccgatctatcactccctcagacgctccgtagtgacctaccattcgaccctcctccctgt
gtgtgcaacggacaaagccaagtggagccagaccagggccttcatgcactagcacctgcagtgagc
attgtgggacaggataccccaccatccagacgataa

Pl_Ulk1/2

>Contig6596

>Pl_Ulk1/2
MEYVDEYEYNKKDIIGHGAFAIVFRGRERKRPDQTVAIKCINKKNLSKSQTFPEKEIEILKELHHE
NVVSLLHFKETASSLFMVMEFCNGGDLADYLHIKGTLSEDTIRFFLGQIAAAMKAIHEKGILHRDL
KPQNLLLSHNSRNKVPHPKEIHLKIADFGFARFLESDMMAATLCGSPLYMAPEVITSKHYDAKADL
WSIGTIIFQCLTGSAPFKAPNPPELKKLYLKARTLDPNIPPGTSRALKDLLIRLLKRNQKDRIEFD
EFFNHDFLSKTLKSTSTSPMPVPGRTYSFSSDSPGERRSLSVSPLSGHMPISSPEEPSPSRATRDY
SPATPSRPSAAKGLQDKLSSGMGSSDLVEDDFVIVQPGLVSELSYETSGASVNVQTTTDVITIRSH
SSPIMSSSRCQPRPTTSPGSTATTTVRRQLPSPSERPSSLPISSSPSTSPNSGRHRVSPKQSPSSL
ISPSKIHAQTGGMEYKPPSPVQATGLSSPGSQYNPGVVHKFYKFHASPTSSSPSPPRIPRVSTEPS
MSPQGAGLYRGSSPQGTSPTNIPSPARRKLSSPARGSPQFFTGPSSLPTIAGSPTKKGFGNEITFT
IGSHGISPSEPLNMPFAKSRRVRASSCCLDGQDMSDSPGRDAIIPRSASSSRLSEPLCLKAAFDNL
AMNPAGSIEGIPGAIGASPPTHPTSFFIGSQSRRSSVLTEGSPSSQGSLTFATSPPNMEGPISFVA
PELPEETLLAPEHTETVDRLNVILGIVEAIVEVAKSRSIPLAESVYNQGSSIFSNSQVCFVSENYR
LAEQLVLYTRSLELLNAALTMAKEEFSAGRLKPSNAVRMVLQELNRVYHLCLTKSRQFCEGSPLQS
LDIDLNSAMITADKLMYSYAIEQCQSAGMDEMFGNSQECLQRYRTAQMLLHGLCLQAGTDHDRNLL
LKFKNALDQRLLFLERQQTPVTPMIGHL

>Pl_Ulk1/2
atggaatatgtggatgaatatgaatataacaaaaaagatattattggccatggcgcgtttgcgatt
gtgtttcgcggccgcgaacgcaaacgcccggatcagaccgtggcgattaaatgcattaacaaaaaa
aacctgagcaaaagccagacctttccggaaaaagaaattgaaattctgaaagaactgcatcatgaa
aacgtggtgagcctgctgcatttaaagaaaccgcgagcagcctgtttatggtgatggaattttgc
aacggcggcgatctggcggattatctgcatattaaaggcaccctgagcgaagataccattcgcttt
tttctgggccagattgcggcggcgatgaaagcgattcatgaaaaaggcattctgcatcgcgatctg
aaaccgcagaacctgctgctgagccataacagccgcaacaaagtgccgcatccgaaagaaattcat
ctgaaaattgcggattttggctttgcgcgctttctggaaagcgatatgatggcggcgaccctgtgc
ggcagcccgctgtatatggcgccggaagtgattaccagcaaacattatgatgcgaaagcggatctg
tggagcattggcaccattattttcagtgcctgaccggcagcgcgccgtttaaagcgccgaacccg
ccggaactgaaaaaactgtatctgaaagcgcgcaccctggatccgaacattccgccgggcaccagc
cgcgcgctgaaagatctgctgattcgcctgctgaaacgcaaccagaaagatcgcattgaatttgat
gaatttttaaccatgatttctgagcaaaaccctgaaaagcaccagcaccagcccgatgccggtg
ccgggccgcacctatagctttagcagcgatagcccgggcgaacgccgcagcctgagcgtgagcccg
ctgagcggccatatgccgattagcagcccggaagaaccgagcccgagccgcgcgacccgcgattat
agcccggcgaccccgagccgcccgagcgcggcgaaaggcctgcaggataaactgagcagcggcatg
ggcagcagcgatctggtggaagatgatttgtgattgtgcagccgggcctggtgagcgaactgagc
tatgaaaccagcggcgcgagcgtgaacgtgcagaccaccaccgatgtgattaccattcgcagccat
agcagcccgattatgagcagcagccgctgccagccgcgcccgaccaccagcccgggcagcaccgcg
accaccaccgtgcgccgccagctgccgagcccgagcgaacgcccgagcagcctgccgattagcagc
agcccgagcaccagcccgaacagcggccgccatcgcgtgagcccgaaacagagcccgagcagcctg
attagcccgagcaaaattcatgcgcagaccggcggcatggaatataaaccgccgagcccggtgcag
gcgaccggcctgagcagcccgggcagccagtataacccgggcgtggtgcataaattttataaattt
catgcgagcccgaccagcagcagcccgagcccgccgcgcattccgcgcgtgagcaccgaaccgagc
atgagcccgcagggcgcgggcctgtatcgcggcagcagcccgcagggcaccagcccgaccaacatt
ccgagcccggcgcgccgcaaactgagcagcccggcgcgcggcagcccgcagtttttaccggcccg
agcagcctgccgaccattgcgggcagcccgaccaaaaaaggctttggcaacgaaattacctttacc
attggcagccatggcattagcccgagcgaaccgctgaacatgccgtttgcgaaaagccgccgcgtg
cgcgcgagcagctgctgcctggatggccaggatatgagcgatagcccgggccgcgatgcgattatt
ccgcgcagcgcgagcagcagccgcctgagcgaaccgctgtgcctgaaagcggcgtttgataacctg
gcgatgaacccggcgggcagcattgaaggcattccgggcgcgattggcgcgagcccgccgacccat

ccgaccagctttttattggcagccagagccgccgcagcagcgtgctgaccgaaggcagcccgagc
agccagggcagcctgacctttgcgaccagcccgccgaacatggaaggcccgattagctttgtggcg
ccggaactgccggaagaaaccctgctggcgccggaacataccgaaaccgtggatcgcctgaacgtg
attctgggcattgtggaagcgattgtggaagtggcgaaaagccgcagcattccgctggcggaaagc
gtgtataaccagggcagcagcattttagcaacagccaggtgtgctttgtgagcgaaaactatcgc
ctggcggaacagctggtgctgtatacccgcagcctggaactgctgaacgcggcgctgaccatggcg
aaagaagaatttagcgcgggccgcctgaaaccgagcaacgcggtgcgcatggtgctgcaggaactg
aaccgcgtgtatcatctgtgcctgaccaaaagccgccagttttgcgaaggcagcccgctgcagagc
ctggatattgatctgaacagcgcgatgattaccgcggataaactgatgtatagctatgcgattgaa
cagtgccagagcgcgggcatggatgaaatgtttggcaacagccaggaatgcctgcagcgctatcgc
accgcgcagatgctgctgcatggcctgtgcctgcaggcgggcaccgatcatgatcgcaacctgctg
ctgaaatttaaaaacgcgctggatcagcgcctgctgtttctggaacgccagcagaccccggtgacc
ccgatgattggccatctg

Pl_Ulk3

>singlet16017

>Pl_Ulk3
MAASLSSRIPKLPGFVFTEKLGSGTYATVYKAYRKGQQREVVAIKVVSKESLNKLSTENLLQEIEI
LKKIKHEYIVELKDFQWDTHYIYLIMEFCSGGDLSQTISKRIALPEATVKTFLRQLACALKFLYSR
NITHMDLKPQNLLLSNSYNPVLKMADFGFAQHITEDVQTDMLRGSPLYMAPEIITDRIYNAKADLW
SAGVIMFECLFGSAPLASSSYAQLAEKIRSPKPIEIPTFVQTSDSCRDLLSRLLKRDPGERIEFED
FFSHPFIDLEHIPCSESLDKARATVIEAVKADQKGEWKEAIRLYLKAMEYFIPAIQYERDASKKDS
LRVRSLEYMRRAEELKGLLKPQKTSLTECNSSQDNAEQTSSSSTESGLVHDIHLLDKMANGYPEMK
VAVKLTRDAIREDSNEEYQIALDLYEQALNQLMPLLQVEQKGKRRDLLLKETEKCMSRAEVLKQYI
EIEKMRPMRQSSLDHRLQGPDHIKDSCVLQ

>Pl_Ulk3
atggcggcgagcctgagcagccgcattccgaaactgccgggctttgtgtttaccgaaaaactgggc
agcggcacctatgcgaccgtgtataaagcgtatcgcaaaggccagcagcgcgaagtggtggcgatt
aaagtggtgagcaaagaaagcctgaacaaactgagcaccgaaaacctgctgcaggaaattgaaatt
ctgaaaaaaattaaacatgaatatattgtggaactgaaagattttcagtgggatacccattatatt
tatctgattatggaattttgcagcggcggcgatctgagccagaccattagcaaacgcattgcgctg
ccggaagcgaccgtgaaaacctttctgcgccagctggcgtgcgcgctgaaatttctgtatagccgc
aacattacccatatggatctgaaaccgcagaacctgctgctgagcaacagctataacccggtgctg
aaaatggcggattttggctttgcgcagcatattaccgaagatgtgcagaccgatatgctgcgcggc
agcccgctgtatatggcgccggaaattattaccgatcgcatttataacgcgaaagcggatctgtgg
agcgcgggcgtgattatgtttgaatgcctgtttggcagcgcgccgctggcgagcagcagctatgcg
cagctggcggaaaaaattcgcagcccgaaaccgattgaaattccgacctttgtgcagaccagcgat
agctgccgcgatctgctgagccgcctgctgaaacgcgatccgggcgaacgcattgaatttgaagat
ttttttagccatccgtttattgatctggaacatattccgtgcagcgaaagcctggataaagcgcgc
gcgaccgtgattgaagcggtgaaagcggatcagaaaggcgaatggaaagaagcgattcgcctgtat
ctgaaagcgatggaatattttattccggcgattcagtatgaacgcgatgcgagcaaaaaagatagc
ctgcgcgtgcgcagcctggaatatatgcgccgcgcggaagaactgaaaggcctgctgaaaccgcag
aaaaccagcctgaccgaatgcaacagcagccaggataacgcggaacagaccagcagcagcagcacc
gaaagcggcctggtgcatgatattcatctgctggataaaatggcgaacggctatccggaaatgaaa
gtggcggtgaaactgacccgcgatgcgattcgcgaagatagcaacgaagaatatcagattgcgctg

gatctgtatgaacaggcgctgaaccagctgatgccgctgctgcaggtggaacagaaaggcaaacgc
cgcgatctgctgctgaaagaaaccgaaaaatgcatgagccgcgcggaagtgctgaaacagtatatt
gaaattgaaaaaatgcgcccgatgcgccagagcagcctggatcatcgcctgcagggcccggatcat
attaaagatagctgcgtgctgcag
