# Supplementary material for: Identification of Cell Death Genes in Sea Urchin Paracentrotus lividus and Their Expression Patterns during Embryonic Development
Source: Genome Biol Evol. 2019 Jan 29;11(2):586–96. doi: 10.1093/gbe/evz020 (PMC6394757; doi:10.1093/gbe/evz020)
Supplement: Supplementary Data [file evz020_supp.zip › Supplementary Information II.pdf]

Supplementary Information II
Accession numbers and protein sequences used in the phylogenetic trees.

Aifm1

>Paracentrotus_lividus_Aifm1
TASFAALRAIRANDPKAKVLVVGDEDATPYMRPPLSKELWFSDDKEGVENLKFKQWNGKEKSIFFEPDAFY
CKPSELSTKENGGVAVLKGHKVKGLNVQKKQATLADGSVITFDKCLIATGGVPRNLGSIEKAGSEVIDRTT
LFRNIKDFKSLDKATEDAKSVAIIGGGFLGSELACALGKRGKQTGMKVMQFFPEAGNMGRVLPEYLSKWTT
DKVSNEGVEVYPDMLLKNVSFNDESKQVELQFKNGEKVQADHVVVAVGLEPNTDLAASSGLEVDDKFGGYR
VNAELEARRDVWVAGDASCFYDIKLGRRRVEHHDHAVVSGRLAGENMTGAAKPYWHQSMFWSDLGPDVGYE
AIGIVDSSLPTFGVFAKATKEDTPKAVVEATGEGIRSETEQAAVETTPAPEAPPLSDDYGKGIVFYMKDKV
VVGMVLWNIFNKMPIARKVIKDGKEYDDVSELAKLFNLHKTEES

>Homo_sapiens_AIFM1_NP_004199
MFRCGGLAAGALKQKLVPLVRTVCVRSPRQRNRLPGNLFQRWHVPLELQMTRQMASSGASGGKIDNSVLVL
IVGLSTVGAGAYAYKTMKEDEKRYNERISGLGLTPEQKQKKAALSASEGEEVPQDKAPSHVPFLLIGGGTA
AFAAARSIRARDPGARVLIVSEDPELPYMRPPLSKELWFSDDPNVTKTLRFKQWNGKERSIYFQPPSFYVS
AQDLPHIENGGVAVLTGKKVVQLDVRDNMVKLNDGSQITYEKCLIATGGTPRSLSAIDRAGAEVKSRTTLF
RKIGDFRSLEKISREVKSITIIGGGFLGSELACALGRKARALGTEVIQLFPEKGNMGKILPEYLSNWTMEK
VRREGVKVMPNAIVQSVGVSSGKLLIKLKDGRKVETDHIVAAVGLEPNVELAKTGGLEIDSDFGGFRVNAE
LQARSNIWVAGDAACFYDIKLGRRRVEHHDHAVVSGRLAGENMTGAAKPYWHQSMFWSDLGPDVGYEAIGL
VDSSLPTVGVFAKATAQDNPKSATEQSGTGIRSESETESEASEITIPPSTPAVPQAPVQGEDYGKGVIFYL
RDKVVVGIVLWNIFNRMPIARKIIKDGEQHEDLNEVAKLFNIHED

>Strongylocentrotus_purpuratus_Aifm1_XP_783530.3
MYRCRAALQGLRPYSRSISTSCRKARLPNGALPRNKVLQANTSRTMAHISTEGVGKNGLYWVIGGAAAVVA
SATYVQTQGLVTWGGEPKRTNVSFAPTGSAAASTEAAPADTAPAGKASPADVPPPVVLPEIPEHATYLLIG
AGTASFAALRAIRANDPKAKVLVVGDEEATPYMRPPLSKELWFSDDKEGVENLRFKQWNGKEKSIFFEPDA
FYCKPSELPTKENGGVAVLKGHKVKGLNVQKKQATLEDGSVISFDKCLIATGGVPRNLRSIEEAGSEVTER
TTLFRNIKDFKSLDKATQDAKSVAIIGGGFLGSELACALGKRGKDTGMKVMQFFPEAGNMGRVLPEYLSKW
ATDKVSNEGVEVHSDMFLKNVSFNGKSKQVELQFSNGDKVQADHVVVAVGLEPNVNLAASSGLEVDEKFGG
FRVNAELEARRDVWVAGDASCFYDIKLGRRRVEHHDHAVVSGRLAGENMTGAAKPYWHQSMFWSDLGPNVG
YEAIGIVDSSLPTYGVFAKATAEDTPKAVVEATGEGIRSETEQAAVETTPSPAASPTTSEDYGKGVVFYMK
DKVVVGMLLWNIFNKMPIARKVLKDGQEHEDVSELAKLFNLHQSEES

>Apostichopus_japonicus_Aifm1_APA32591
MYRTILRSVRPLSRRATRQSRQKNGSILIGQTVRQMAVESSEGIGSKGTLWLVGGAAAFVGLGTFVYSNG
YIVFNNTPDKSVLSKSVVADTEKDVPLASTEGNPPATEDEAASQAASSSASEPVELPAIPGHAPYLLIGA
GTASFAAFRAIRANDSTAKILVIGEEDFLPYMRPPLSKELWFSDDKEKVNDLKFQQWNGKERSVFFEPDS
FYCKPSELPIKEKGGVAVLSGHQVVRLDVHKKTVTLDDGTKITYDKCLIATGGNPRNLPEIEKAGSDVME
RVTLFRNIKDFKRLDEATQTAKSVAIIGGGFLGSELACALGKRGKEIGAKVTQFFPEAGNMGRVLPEYLS
KWTTNKVESEGVDVITGVVLKSASLNTDSNQVALTLSNGAEVLADHVVVAVGLEPNTALSITSGLEVDDR
LGGYRVNAELEARRDVWVAGDASCFYDMKLGRRRVEHHDHAVVSGRLAGENMTGAAKPYWHQSMFWSDLG
PNVGYEAIGIVDSSLETVGVFAKATEKDTPKAVVEATGESIRSTTEEAAPSQAPTTSTAEPPVQSSTEDY
GKGVVFYMKDETVVGIVLWNVFNRMPIARRVIKEGKQYSDLSELAKLFSLYDKED

>Ciona_robusta_Aifm1_XP_002131727
MFRCVRLCEHSSSISRSLLNKNAPKTQAVIVSRYQDQVTPVHCKQNRAFYSQKATFHSDQSSNYGSGFCII
GGTLFFGIGIYKYLTESKANAAEAQTQGKSVEQATEDVFGVPPAAEFPSQVQYLLIGGGTASYAAMRAIKK
RDVTAKILIISEDSYPPYMRPPLSKELWYSDDEEAAKKLQFKSWNGKWRDVYFEKPDYYCAPSDLPAKSEG
GVAVANNTRVVSLNPTDNIATLKDGTEIKYDKCLIATGGQPKNLPVFRKASAEIQKKVSTYRTASDFTELN

NITDGVKSITVIGGGFMGSELACALGHKGQKVGFEVNQIFPENGNMGNVLPEYLTKWTTSKVKSEGVNVIT
DTVVNNVTMQDGRLKLQLRTGQEILTDHVVVAVGLGIDTSLAESGGLETDERFGGYRVNAELQARSNIWVA
GDASCFFDAKLGRRRVEHHDHAVVSGRLAGENMAGGQKSYYHQSMFWSDLGPDVGYEAIGIIDSALPTVGV
FAKATEKDTPKAAEGAGLRDDGAENPAENTEKQPTTTTTTASSEEFGKGVVFYLRQQKVVGVLLWNVFGRM
PIARKMIRDQQAYDDYTEVAKLFKIHSEELD

>Danio_rerio_Aifm1_XP_005165182
MFKCKTVWNKLAPLARASSTLCLQNARKSVLRHGRRLEMVQRAQMSSGPPGGGGENAVYFVLVGAACLGGG
IYAYRTVSQDSERYNERMRQIASRQSQESKPAKALAEVAESKEEPVAEEPTLIEPAVEESPVTEDIVESPE
AVAEPIVKSSIPEPAVEIAVTEPVVEASVPESVIESPLMEPVVEASVQEPIIEATATETVAEAPEVIQSEE
PIAPVVESEPVPVVSTEHVPEASSVSESAPETSPAAAPAAEPIDPTAPHLDVLEHAPYLLIGGGTASFAAA
RSIRARDPGARVLIITEESDLPYMRPPLSKELWFSDDPKVTESLRFKQWNGKERSIYFQPPSFYVSPADLA
KVENGGVAVLTDSKVVHMDVRGNKVKLSDGSEISYEKCLIATGGVPRNLQVIDRAGEEVIKRTTLFRKIED
FRSLEKISREVKSITIIGGGFLGSELACALGRRSADPGLEVMQLFPEKGNMGKVLPEYLSNWTTEKVRKEG
VNVITDAVVKNVTYKNDKLEIKLKDGRLVKTDHIVAAVGLEPSVELAKSAGLEVDSDFGGYRVNAELQARS
NIWVAGDAACFYDIKLGRRRVEHHDHAVVSGRLAGENMTGANKPYWHQSMFWSDLGPDVGYEAIGIVDSSL
PTVGVFAKATAKDTPKAATEQSGTGIRSESETEAVAGVLEVGTVTPAPPVQQKEDYGKGIIFYLRDKVVVG
IVLWNVFNRMPIARKIIKDGEAHTDLNEVAKLFNIHED

>Saccoglossus_kowalevskii_Aifm1_XP_006825404
MSSQGSNEKLGPKIVVAVGAAAVLVGGAYMYYEQKPKQEKKDTPSAPAEKRQSQIPSSNVSKVESSESAPG
LSKLQPQDAGWVPPSYTYPDLDEAPELPGYIPYLLIGGGTASFAAFRAIRAKDSTAQILIVTAEDHLPYMR
PPLSKEFWFSDDKKAVENLRFKQWNGRERSIFFEPAAFYCRPNELLYKEKGGVGVATGRKVVELDPVEKKA
YLDNGWEIYYDKCLIANGGTPRTLPALVNADDKIKKKTTLFRTIDDFKELDRITNNVGSVGIVGGGFLGSE
LACALAKRGKENGLKVHQIYPEFGNMARVLPEYLSHWTTNKIIEEGVDVMPKHRVKSVSLDNGKLVLKMKE
GKDEVKVDHLIVAVGIEANTDLAKTSGLEIDDAHGGYRVNAELEARSNVWVAGDAACFYDIKLGRRRVEHH
DHAVVSGRLAGENMTGAGKPYWHQSMFWSDLGPNIGYEAIGITDSSLPTVGVFAKATEKDTPKAASEASGE
AMRSDSEMSAMNEESVPSTDQSAAPVLKVPNMDENFGKGVIFYMKDKIIVGVILWNVFNKMPIARQILKEE
KEHEDLNEVAKLFAIHES

>Caenorhabditis_elegans_Aifm1_NP_001263729
MKPSESTEENTTTTSADGLLHCEYVIIGSGTAAYYASLSIRAKQAEAKVLMIGEEPELPYNRPPLSKELWW
YGDETSATKLAYTPLSGKKRDIFYEVDGFFVSPEDLPKAVHGGVALLRGRKAVKICEEDKKVILEDGTTIG
YDKLLIATGVRPKKEQVFEEASEEAKQKITYFHYPADFKRVERGLADKSVQKVTIIGNGLLASELSYSIKR
KYGENVEVHQVFEEKYPAEDILPEHIAQKSIEAIRKGGVDVRAEQKVEGVRKCCKNVVLKLSDGSELRTDL
VVVATGEEPNSEIIEASGLKIDEKLGGVRADKCLKVGENVWAAGAIATFEDGVLGARRVSSWENAQISGRL
AGENMATAAADGKSEGKAFWYQPSFFTKFAPHLHINAIGKCDSSLETVSVHAEPDKDTPLEKAVVFYKSKE
DGSIVGVLLLNVFGPSLDVARRIIDDRKKVDEYKEIAKLFPLYDPVKSDEDDAKSA

Bax – Bcl2

>Paracentrotus_lividus_Bax
MAEGGSDTPCDETDASTRLRIEQTRFERQVSRDDVGEQATVLLQHFIVERFQQDGFENAPGLDELRAGHAV
SSEQEVVWSEVGSMLRSIGDELDRDQELQRMINSIPADSPIEAIIAVAHVVFLDGDISWGRIVGLFYFAYR
MAARAIDSVLEKSFPNWINKLIKEVVKFLVLKFAHWIISKGGWLAIREYMGSPTWIWGTLVTLSASCLLFS
LYKLSR

>Strongylocentrotus_purpuratus_Bax_XP_011667030
MYCNRNCDSSTTSLHFEQTRIQLERQVSRDDVGEQATVLLQHFIVERFQQDGFADAPGLDDLRAGLAVPSE
QEVVWSEVGVMLRSIGDTLDRDRELQRMISSVPVDSPIEAIIAVAHVIFGDGDITWGRIVGLFYFAYRMCA
RAIETIMDKSFPSWINKLIKEVVKFLVIKFAHWIVNKGGWLAIREYMGSPTWIWGTLMTLSASCLIFSFYK
LSR

>Homo_sapiens_BAX_NP_004315
MDGSGEQPRGGGPTSSEQIMKTGALLLQGFIQDRAGRMGGEAPELALDPVPQDASTKKLSECLKRIGDELD
SNMELQRMIAAVDTDSPREVFFRVAADMFSDGNFNWGRVVALFYFASKLVLKALCTKVPELIRTIMGWTLD
FLRERLLGWIQDQGGWVRLLKPPHPHHRALTTAPAPPSLPPATPLGPWAFWSRSQWCPLPIFRSSDVVYNA
FSLRV

>Saccoglossus_kowalevskii_Bax_XP_006822173
MAEGGPPDEYLREDEGAVGGAERQQSQEQDVIGAQATQLLMEFIIERFQRDGIENAPTMEEIQDPDVLTPM
QEENWGQIGATLRTIADEMDRDYELQRMIDRVPTNCSVDAVIAVARVIFDDGIVNWGRVVALFYFAYRMCV
KAINSMMSQYLPGWMTRLIREIVKFLVEVFAAWIISRGGWGAIIEYLGSPAWQFYSLCAISFTCFVAAVVR
MNWR

>Branchiostoma_belcheri_Bax_XP_019646409
MSETQSEEKKDPPETGDSAPATGHHIVPPPGHHVATPSDHGIPGHHGYQHGNTGHHGVMDHHGNMDHHGN
LDHHGNLDHHGNGHGSTGPGHFLYGSDQNILTDIPGLGGEPQREEEQQTQQPAAGSQGEGQQQEEEPLVR
SPQETEENVAAQTESVVRDFLFQRHQREQAREAQVGECTPSMPELVPDVNPLSRESEVGRKLAMIGDEIE
TRYESEFKAMIKTLRVTPSTAYEAFAGVARRLFRDGINWGRIVALLCFGYRMAVDVLERGIPDFVRQIIK
YVVQFIISERIARWIAEHGGWRGVLSYTLNEGFQALGTVFVVAAVTVIAAVWLFRRS

>Ciona_robusta_Bax_XP_002123003
MASNNTNQNTNMNNSAGVEDCNDGEGDGIAQTREEPKVPPAVRPRPMTAVGERRRPAAGRGEARDMGGASA
YANNSRSNEPTNYESGDSAASTNNRNTNTYSGYDDQGPSTSSKCTGATSTQRRQRTYSQQVSREESRIGEQ
ARFLLNMFIQDRAGVENSPVEPVVRNVCQADMSPQSAGFTDENLSDIAVTLRRIGDDMSRDIQLNRFIDQV
PLKSTKDIFIKVCLQMFEDGNFNWGRIVALFYFAYRLIVRSLLSGLDSLPWIRELISWVVDFIVKKFAKWI
ISRGGWTMIKEWFGISSQTFGVLICVTLAIVGWAVFKKD

>Danio_rerio_Bax_NP_001314995
MEALLDYVVRIGSGNDQTLDAGSAVLFNFIFEWLHQHLDKEAEITCWLQNNLGIVEKSDPSHKDAIECMVR
IANEMEGNEELQGMLNSALLNPTLEHYILVVNGTFSDVTLSWGSVVALFYVACRFVVKAAEINSVDLVRSI
INWTMPFIRKTCILTWIREQGGWGAIRSYFGTPTWQTVGVFLAGVLTVGLVLYKM

>Paracentrotus_lividus_Bcl2
MPDISTASIVADYLRYRLQCLGNGVDAEENDCLSTVMLVTEAVSSLHEAIVNLSEKLMNDYRDSFSQAYNQ
VLAHGDGSMNYGIFKAGMKSMVKDTVPSWGRIVAFFSFGGALCVWCVEKDIRAFPTVEELMVAYINSHLIT
WIHANGGWTNVDLDKIDSETWSEISSRNPSPQGAQSPSHTTPP

>Strongylocentrotus_purpuratus_Bcl2_XP_003730897
MPDISTASIVADYLRYRLQCLGNGVDAEENDCLSTVMSSVENVPEDLHEAIVNLSEKLMNDYRDSFSDMPN
QLKITENTISSTFKAVTSELFVDGVISWGRIVAFFSFGGALCEWCQEKDIPRAFPTVEELMVAYINSHLIT
WIHANGGWVMK

>Homo_sapiens_BCL2_NP_000624.2
MAHAGRTGYDNREIVMKYIHYKLSQRGYEWDAGDVGAAPPGAAPAPGIFSSQPGHTPHPAASRDPVARTSP
LQTPAAPGAAAGPALSPVPPVVHLTLRQAGDDFSRRYRRDFAEMSSQLHLTPFTARGRFATVVEELFRDGV
NWGRIVAFFEFGGVMCVESVNREMSPLVDNIALWMTEYLNRHLHTWIQDNGGWDAFVELYGPSMRPLFDFS
WLSLKTLLSLALVGACITLGAYLGHK

>Saccoglossus_kowalevskii_Bcl2_XP_002740789.2
MQQPQNCVGVRNWLSCKTQKTVSVFRDFFDLLTYSCMNPTTHNANTMVNLHSRTLVVDYIFYRLNSRGLEW
PTCPRLSPPTKVNFTMRALGDEFETRYRDVFQGMCDQLHITPSTAYPTFTAVTNELFTEGITWGRVVAMYA
FGGALAVQCVDREMAQYVDRVVDWITQYTDNNLSQWISENGGWNGLVHFYEGTRDNPSNSWPSGKALFGVA
ALGLVTLGATLLTKT

>Branchiostoma_belcheri_Bcl2_XP_019630513

MVTSDSRSIVTDYVFYKLNKKGLPWPSAPPLPPPTDAHRLMRELGDKFEERYREQFDEMGDQLHLTPDTAY
QKYHNVVAELFREGVRWGRLVALVAFTGACTVVAVEREMPQFVDRYVDWTVQYIDNNLAQWLQENGGWEGF
EDFARKARSNEKDENSWWWRGILMGAMAVTAVGAIVARS

>Ciona_robusta_Bcl2_XP_002127107
MVQQTRRVVEDYVLFRIHETIASESYDSFSSRCKLEDQASRGFPLPQTNPARLNVSIRKIAAEYERRYRET
FPDLLEEIKTVNMSTDDVDKTFSRICKDLFQLPLKRNYPDNAMKSQATSNSKIFIQSDILEVEDNHVKWGH
VIALLVFAAIVAVRAVELKKHEQVDAIVSWVSKFIDTELTGWLNKQGGWENVIEWSEAGETRLQLQKDTSF
DPNNFSSSFRSAISVGVVAACVGLGALIMTRK

>Danio_rerio_Bcl2_XP_001341214
MAQENVYNNRSIVENYIHHKLWKKGYVWEVNGHDSVSNGLSMGRQENSVVSPSSRHDPYSALHNVLREAGD
ELESLYQSDFAEMSKQLHVTSITAHQRFNAVIDELFRDGVNWGRIIAFFEFGGTVCAECVNKEMTGQVDNI
AVWMADYLNGPLHGWIRENGGWEAFVELYSSQRDSVFHSSWSSIVTVFGLAALGAVGLTIGAYLAQQ

>Homo_sapiens_BID_NP_932070
MCSGAGVMMARWAARGRAGWRSTVRILSPLGHCEPGVSRSCRAAQAMDCEVNNGSSLRDECITNLLVFGFL
QSCSDNSFRRELDALGHELPVLAPQWEGYDELQTDGNRSSHSRLGRIEADSESQEDIIRNIARHLAQVGDS
MDRSIPPGLVNGLALQLRNTSRSEEDRNRDLATALEQLLQAYPRDMEKEKTMLVLALLLAKKVASHTPSLL
RDVFHTTVNFINQNLRTYVRSLARNGMD

Parp

>Paracentrotus_lividus_Parp
MSPSAGLIFEGPHPIGKLLTSAAPPTSTLLYLVAFITRAFSEPAKSKSMSLLHLNRCQRHTSEKDTRLNLY
LVYTVTRVAGLSCHVYKEKGLLYSATLGLVDISRGTNSYYKLQLVQHDTQSRYWVYRSWGRVGTTIGGNKK
DNFGSDVKKAKEHFRAVYLEKTGNGFGAKNPIKRPMKFFPLDISYGEEEDRIISSKERAGKTSKLPKEVQS
LMKMIFDLEELKKTMLEFEIDLEKMPLGKLSKKQIEDAYKVLTDLQKPFWLCLIRLHAAVHQVCECGEEET
NKTQMLDSLLDIEIAYSMLKETGEEGVDPVDVHYEKLKCPMEVVDKKSDEFKMISDYTTNTHAATHNWYRL
TVEEVFRIDREGEGSRFKPFKKLHNRQLLWHGSRKTNFGGILSQGLRIAPPEAPATGYMFGKGIYFADMVT
KSANYCYANASSNIGLMLLTDVALGDMYELRGAKGMSKPPAGKHSTKGLGRTCPDPSGLRTIEDNLQVPMG
FDSTDKSYKTNLPIVLGAVPQNHKSILGQAIGTRVVELTHVSMGCPKTYDGFVVRPPGEVRYRPEIYVNSK
SHRRRISIKRSPHTSCAG

>Strongylocentrotus_purpuratus_Parp_XP_011667940
MTLLALPIITLALARLPYVALAFQGINSCRVPIYTTWVECGKCRSMSCQRTLVRRRDSNRGPCDRQSGGFP
SLRWDDQQRIKETLSGKETGGALKSADPDAEVDTITTLAQFIVGYASSGRAQCHGSGCSEKIAMKTVRIAL
LKPMEEHLDYGPTPCWYHVDCFVNAKHKENDYTEWPANGKASMLTDFNLLKDTDKKMLEEKFKKAMPKTKG
QGKGKTDPPPAKKAAPKISKLDRDLKTQNDQLWAIRDQLYRWVKNNGLKDILEANDQLVPPGESRLLDSVA
DGMMFGALEKCPECENGQLVVSSSGLSFKCTGNLTSWTKCQYRSKDVPRSKTWHIPEYLKEDIDYLKNFKF
VAYNKGKRLFPEEEEGASTSAASQDGAGDGKRKIVKKPEDKALYNCTIVLHGKLSKSNKQLKTTIQDLGGE
VATKITPETACVISNEAEVKKMNKKMKEAQSNDVHVVSEDFLDAVKNGGAALMITQHSISTWGSDPTTRIA
DTVAGQLPTKSIGQLQREKDESKFTSVMPKTSKMQLKGGAVVEDQNLGHATHVHKEKGVLYSATLGLVDIS
RGTNSYYKLQLIKHDSQAKYWVYRSWGRVGTTIGGSKSEHYGSDLAGAKEMFKNTYLEKTGNKFGAKNPIK
HPMKFFPLDISYDEEEERITSSKERAGKTSQLPKQIQSLMKMIFDLEELKKTMLEFEIDLEKMPLGKLSKK
QIEDAYRVLTDLQKLLTDKAPRSGILDASNQFYTLIPHNFGLKSIPLLDSLDIIQAKTQMLDSLLDIEIAY
SMLKETGEAGVDPIDVHYQKLKCPMEVVDKKSDEFKLIKEYTDNTHAATHNWYRLSVEEVFRINRDGEGTR
FKPFKKLHNRQLLWHGSRKTNFGGILSQGLRIAPPEAPATGYMFGKGLYFADMVTKSANYCYANASSNIGL
MILSDVALGDMYELYGAKGMSKPPAGKHSTKGLGRTCPDPSGLVTIEDNLQVPMGKGCDSNINNTSLLYNE
YIVYDVAQVQMRYLIKMKFNYNC

>Homo_sapiens_PARP_NP_003757

MAESSDKLYRVEYAKSGRASCKKCSESIPKDSLRMAIMVQSPMFDGKVPHWYHFSCFWKVGHSIRHPDVEV
DGFSELRWDDQQKVKKTAEAGGVTGKGQDGIGSKAEKTLGDFAAEYAKSNRSTCKGCMEKIEKGQVRLSKK
MVDPEKPQLGMIDRWYHPGCFVKNREELGFRPEYSASQLKGFSLLATEDKEALKKQLPGVKSEGKRKGDEV
DGVDEVAKKKSKKEKDKDSKLEKALKAQNDLIWNIKDELKKVCSTNDLKELLIFNKQQVPSGESAILDRVA
DGMVFGALLPCEECSGQLVFKSDAYYCTGDVTAWTKCMVKTQTPNRKEWVTPKEFREISYLKKLKVKKQDR
IFPPETSASVAATPPPSTASAPAAVNSSASADKPLSNMKILTLGKLSRNKDEVKAMIEKLGGKLTGTANKA
SLCISTKKEVEKMNKKMEEVKEANIRVVSEDFLQDVSASTKSLQELFLAHILSPWGAEVKAEPVEVVAPRG
KSGAALSKKSKGQVKEEGINKSEKRMKLTLKGGAAVDPDSGLEHSAHVLEKGGKVFSATLGLVDIVKGTNS
YYKLQLLEDDKENRYWIFRSWGRVGTVIGSNKLEQMPSKEDAIEHFMKLYEEKTGNAWHSKNFTKYPKKFY
PLEIDYGQDEEAVKKLTVNPGTKSKLPKPVQDLIKMIFDVESMKKAMVEYEIDLQKMPLGKLSKRQIQAAY
SILSEVQQAVSQGSSDSQILDLSNRFYTLIPHDFGMKKPPLLNNADSVQAKAEMLDNLLDIEVAYSLLRGG
SDDSSKDPIDVNYEKLKTDIKVVDRDSEEAEIIRKYVKNTHATTHNAYDLEVIDIFKIEREGECQRYKPFK
QLHNRRLLWHGSRTTNFAGILSQGLRIAPPEAPVTGYMFGKGIYFADMVSKSANYCHTSQGDPIGLILLGE
VALGNMYELKHASHISKLPKGKHSVKGLGKTTPDPSANISLDGVDVPLGTGISSGVNDTSLLYNEYIVYDI
AQVNLKYLLKLKFNFKTSLW

>Saccoglossus_kowalevskii_Parp_XP_006812383
MADEHHDYPFKAEYAKSSRASCKFCKGSISKESLRLAKMVQSPHFDGKMPNWFHYSCFWKRNKATSHGEFG
GLETLRWDDQQKIKDKIAGKDSGATKGEGDGDDTGSLGDEFSLGYAKSNRSVCRGCNEKIMKDTFRIARIL
METQRGIHMAVEKWHHLDCFCEKLDELGWTEGCSPEDIPGFKVLKDEDKKELLAKLKTDKKNVKRKTTETD
GTAKTKKPKTKKETKEEEALRVQSQFIWGIRDQLYKHVPNSDLKELLEENNQNIPSGESKLLEYATDGMAF
GALLPCPECKEGQLVVGSSGYRCTGNITSWTKCQYKTKEPKRKKWKIPESLNDIDYLKKFKFKAFNKGVRV
FEDKPEISSPPSAAASASTSSSRSALQDCKIVLVGKLSESNKDITKKIKQLGGEVVTKINKNVACVISTQD
EVNNMSKKIKEAKAAGVHVVSEDFLNTVKKGGAALMIQQSSIANWGTDPVTRISVGSTAVDSAIKSGALKS
GVSAREEKMYTKKVPDKLKMTVKGGAAVDPDSGLEDEAHVYSAKGSVYNAILGLVDVTKGTNSYYKLQLLE
HDNTKKWYVFRAWGRVGTTIGGNKVEKFHSLLNAMDHFCSLYGEKTGNDFGTKDFQKYPNKFYPLEMDYGQ
EEEELKKMNMEVGSNSKLHKSIQELIKMIFDIDEMKKTMLEFEIDLKKMPLGKLSKRQIESAYSVLSELQK
LLDEDKATDSAILDCSNRFYTLIPHDFGMKKPPMLDNIDIIKNKTQMLDNLLDIEVAYSLLKGGDSGKDPI
DANFEKLKCGIEVLEHKSDEFTMIEKYMKNTHAATHTQYSLELLDVYQLDRAGECSKYKPFKQLHNRQLLW
HGSRTSNYAGILSQGLRIAPPEAPVTGYMFGKGVYFADMSSKSANYCRTTPSNNIGLMLLCEVALGNMYEL
TNAKSLSKPPSGKHSTKGLGATGPDPTAAITTKDGVTVPLGKGAIDGSLKTSLLYNEYIVYDVAQVQMKYL
CKAKFNYKIY

>Ciona_robusta_Parp_XP_018669690
MDENDLPYLAEYAKSSRASCKLCMSHIEKDSLRIALMVQSRHFDGKQPNWHHYTCFFGRCRPKFVDEISKF
HNLRWDDQEKIRAQIERGGGGGPSKKSSKKTLQDFVVQYALSNRSTCKKCEEKIEKDEIRISHKEIDPEKP
QVGLIDRWHHVGCFIKSRKDLGWIDGQFTSDMLKGFKGLDSEDRATISKHLDKKKKGKKKTKIVKEEEEND
ENLDAMNKLLRDQSAKLWKMIDHLKNMDLKKKELVEILEENSQKVPKGLDKIFERVADGMLFGRLPLCTLC
GDGQLVASSRGFYQCVGNISGWTSCTNTTLDVKRSPWIIPQDLHEDFPYLKKYKYKPTVRIFPPRPKPKPL
KDKKVVIIGKLSKPRDELKQLVENLGGSVTTSLNKAYCCVTNQAEVETTNKRIKSAEIQDVFCVSEEFLDC
CKDLSTPLNSLLPFLKKYSLCPWGSERLNNAALKRSMAEESRSLSTKKQKLVVKSGAAVDPESNLEDETEV
LQHDGRSYTATLAMVDMKSGTNSYYKLQLLADTSGRSWHVFRSWGRVGTSIGGTKVEKFRTLDNALQNFNE
IYTEKTGNDFGSKDFVKQPHKFYPLEIDYGMDDEKKVQHLDTAGKSSKLKQEVQDLVKMIFNIESMKKAML
EFEIDLNKMPLGKLSKRQMQQAYLVLNEVNELVTAGDHGSGKVLDCSNRFYTLIPHDYGVHQPPLLDNLDI
IKAKTEMIDNLIDIEVAYNLLTTTTEDTQTDPLDLSYEKLQCDILPLDKDSEEFKMVEKYTQLTHASTHNQ
YKLTIEHLFKINRKGELSRFRPFQSLPNHKLLWHGSRMTNYAGILSQGLRIAPPEAPVTGYMFGKGLYFAD
MVSKSANYCSTSSRNPIGLALLADVALGNPYELKYAKDVRKLPKSKHSVIGLGKTVPDPSTHITVDSNLVV
PMGKGVEADLGKDKETSLLYNEYIVYDVAQVNLKYLVQFKFHYNTLW

>Branchiostoma_belcheri_Parp_XP_019618726
MLAYRVQEAAKISMADDEGKAYKAEYAKSNRASCKLCKGNITKDSLRLARMVQSPHFDGKVPNWFHYSCFF
KKCKPSSTVEFSGVTGLRWDDQEKLKKTIGGEGSSAGADAGGDEVDAPGVQVFSVEYAKSNRSACRGCSEK
IDKGLVRISKKVDEGDQWGPKDLWHHVDCFVEKREELGFTTDMAPSVIQGYKKLSKDDQDILVKKLGSGTT
GKRKAKGSNGAAAKKVKAEETEEEKKLKEQSKLVWKIRDELSKSMENSDLKELLLANDQDIPSGESALLDR
LSDGMAFGALQRCPECKHGQLFYRSDGYHCSGNLTEWTKCIYTTREPKRKKWIIPDDLKEEVPFLKKFKSK

VAARVFSAAHVAAASESTDSFSSSSRADEKKPLHHVKVVLGKTTKSKAEMTKAIEKLGGTVASKVDSTVAC
VISSKEDVQKMSKKIKDAKAADVHVVSEDFVTDVEKGGAALLIMQKSIASWGSDPHSRIATVEEKPSKSKS
KSKYEEMESGTKKLKMMVKGGAAVDPDSGIEHSAHVLEDKGKVYNAVLGLVDLVRGTNSYYKLQVLEADKG
NRWYVFRAWGRVGTTVGGNKLETFHSRQGALEQFLNLYEEKTGNEFGTKNFVKYPKRFYPLDIDYGQEEED
LQKLKIKPGSKSTLAREVQEIIQMIFDIESMKKAMVEFEIDLKKMPLGKLSRKQIESAYSVLTELNGILTG
ERSATRILDASNRFYTLIPHDFGMKKPPMLDNEEVIKAKTTMLDNLLEIEVAYNLLKSGDDGEGKDPIDAH
YEKLKCKMEVVEKASDEFAMVQEYVKNTHAKTHSHYTLEVEELFKIAREGEASRYRPFQQLHNRQLLWHGS
RVTNYAGILSQGLRIAPPEAPVTGYMFGKGLYFADMVSKSANYCATSTASPTGLLLLCEVALGNMYERKHA
EYVSKLPKGMHSTKGLGATGPDPGATKTLPNGTQVPIGHGVPSGVSGSSLLYNEYIVYDVAQVEMKYLIRM
KFNYKSLW

>Danio_rerio_Parp_NP_001038407
MADSQDDKLYKAEYAKSGRASCKKCKDNIAKDSLRMAIMVQSPMFDGKVPHWHHFSCFWLRAAVQSPSDIS
GFTDLRWDDQEKVKTAIESGGATGGKGGQKGAAKGEKTLNDFAVEYAKSNRSTCKGCDQKIEKDQIRVSKK
TVDPEKPQLGLIDRWYHTGCFVSRREELIFKPEYSAAQLKGFAVLRDEDKEELKKRLPAVKSEGKRKADEV
DGGVSKKQKKEDEKLEQNLKDQSQLIWGIKDKLKKFCSINDMKELLIANSQEVPSGESNIVDRLSDCMAFG
SLKPCETCKGQLVFKSDAYYCTGDISAWTKCVFKTQTPDRKDWVTPKEFSEIPFLKKFKFKRQDRVFPKDA
PPAAATPSSGSTTSAATSVSSASKNLTEAPADKPLTGMKLLAVGKLSKNKDDLKKFVEDLGGKITGTASKA
ALCISSKKEIEKMSKKMEEVRDAGVRVVADDFLTDIKESGKALQELISLHAISPWGAEVKVEAPAAAAATK
STGAHSSKSTGKVKEEEGGSKSKKMKLTVKGGAAVDPDSGLENCAHVLEQNGKIYSATLGLVDIVRGTNSY
YKLQLLEDDVQKRYWVFRSWGRVGTTIGGNKLDKFYDKNSAMDNFCGVYEEKTGNAWASSNFTKYPNKFYP
LEIDYGQDEEAVKKLTQSAGAKSQLEKPVQDLIRMIFDVESMKKAMVEFEIDLQKMPLGKLSKRQIQSAYS
LLSEVQQAVADSSSESLILDLSNRFYTLIPHDFGMKKPPLLSNVDYIQQKVQMLDNLLDIEVAYSLLRGGV
ENNEKDPIDINYEKLKTKIEVVDKSSHEAQLILQYVKNTHAATHNTYTLDVEEIFKIEREGEYQRYRPFKE
LPNRQLLWHGSRTTNYAGILSQGLRIAPPEAPVTGYMFGKGVYFADMVSKSANYCHTSQADPVGLILLGEV
ALGNMHELKKASHITKLPKGKHSVKGLGRSAPDPRATVSLNGVDIPLGKGMNTNIDDTSLLYNEYIVYDVS
QVNLKYLLKIRFNYQTSLW

>Caenorhabditis_elegans_Parp_NP_491072
MIHSNEPLPYAIEYAKSGRSNCKTCKKNIALDQLRMSMNRPSTFFDGNMDSWFHYNCFWIKMIRGRDDINI
SSIRGVDWLRWEDQEKLRQEIQHFKTASPPTLTPLCSTTTVILSTIKTEKSLSNRGKCGKCGQNFERGEIK
AHNKGKANHFKCFLQEFDKISGTVEDIPGWADYEENFKIKAVGEYVEALAAKRRSTEPATPASASPTPPEA
ETPVLSAEGSPESSNKRPASSEIIEIDGEGNPDENDFAKKRRMKKEARLMEVQKKRMKKQSDLLWEYRQIF
ERMPYTDKISILRENEQDIPEGHDPTAQVIERLVDNALFGCPIICQTCSNGKIVYNSSCRTYVCTGYATEY
SKCTYESKNPIRTPFEVSHRLTEKHKLQDIVFNQMSERLYIGEEDGESVVKIDKRKSKGGTRGEQFIYAAE
AFDSTNNVPIKVGDLTSTNTHIIKKGTVVDAKFALADRCHVFKNEIDGSLYQATLSFTDLTQNKNSYYKIQ
LLKDDQRENYYVFRSWGRVGTEVGGNKHESYSNSNEAILKFQDVFHEKTKNDWIYRKHFRKMPGMFSYVET
DYSEFAQITDTEITPGSKTLLPKSVKEVVMSIFDVENMKSALKSFEMDVNKMPLGRLSHNQINLAFEVLND
ISDLLVKLPIDASRILDFSNKFYTIIPHNFGMRVPEPIDSFHKIKEKNNMLNALLDIKFAYDQISGGDVPA
STSLGIDPVDINYQKLKCIMEPLQQGCDDWNMIHQYLKNTHGATHDLKVELIDILKLNRDNESSKFKRHIG
NRRLLWHGSGKMNFAGILGQGLRIAPPEAPVSGYMFGKGVYFADMFSKSFFYCRANAKEEAYLLLCDVALG
NVQQLMASKNVSRQTLPAGFQSVQGLGRQCPREIGSYNHPDGYTIPLGLTYMQLQGKQDVDYHLLYNEFIV
YDVDQIQLKYLVRVKMHHARHL

>Homo_sapiens_DNAligase3_NP_039269.2
MSLAFKIFFPQTLRALSRKELCLFRKHHWRDVRQFSQWSETDLLHGHPLFLRRKPVLSFQGSHLRSRATYL
VFLPGLHVGLCSGPCEMAEQRFCVDYAKRGTAGCKKCKEKIVKGVCRIGKVVPNPFSESGGDMKEWYHIKC
MFEKLERARATTKKIEDLTELEGWEELEDNEKEQITQHIADLSSKAAGTPKKKAVVQAKLTTTGQVTSPVK
GASFVTSTNPRKFSGFSAKPNNSGEAPSSPTPKRSLSSSKCDPRHKDCLLREFRKLCAMVADNPSYNTKTQ
IIQDFLRKGSAGDGFHGDVYLTVKLLLPGVIKTVYNLNDKQIVKLFSRIFNCNPDDMARDLEQGDVSETIR
VFFEQSKSFPPAAKSLLTIQEVDEFLLRLSKLTKEDEQQQALQDIASRCTANDLKCIIRLIKHDLKMNSGA
KHVLDALDPNAYEAFKASRNLQDVVERVLHNAQEVEKEPGQRRALSVQASLMTPVQPMLAEACKSVEYAMK
KCPNGMFSEIKYDGERVQVHKNGDHFSYFSRSLKPVLPHKVAHFKDYIPQAFPGGHSMILDSEVLLIDNKT
GKPLPFGTLGVHKKAAFQDANVCLFVFDCIYFNDVSLMDRPLCERRKFLHDNMVEIPNRIMFSEMKRVTKA
LDLADMITRVIQEGLEGLVLKDVKGTYEPGKRHWLKVKKDYLNEGAMADTADLVVLGAFYGQGSKGGMMSI

FLMGCYDPGSQKWCTVTKCAGGHDDATLARLQNELDMVKISKDPSKIPSWLKVNKIYYPDFIVPDPKKAAV
WEITGAEFSKSEAHTADGISIRFPRCTRIRDDKDWKSATNLPQLKELYQLSKEKADFTVVAGDEGSSTTGG
SSEENKGPSGSAVSRKAPSKPSASTKKAEGKLSNSNSKDGNMQTAKPSAMKVGEKLATKSSPVKVGEKRKA
ADETLCQTKVLLDIFTGVRLYLPPSTPDFSRLRRYFVAFDGDLVQEFDMTSATHVLGSRDKNPAAQQVSPE
WIWACIRKRRLVAPC

Pink

>Paracentrotus_lividus_Pink
MSFRHGLQAIARVVRRRLQQQAQQHTHEHHAGRQRPDVATSNPAQRYHSSRPTGSDVRLNTQNLAIRAARV
WRQTPSSWSSNGSRFVQSSRPVLPLLLGFAGIGLANQDGEGFAARGGIDLETALQVVQTVFDDRGLKAGQD
TSKEFPDNLASYAFSKTVLAKGTEGAVFAAKRRDTVLPSKGDEEFADAQARYMPVDQPIDGGHIGEQPKAE
MKVCDEGECNLAIKMMFNYTGTGGSKPADIMAEFGAEQLPLVLAGGSFSGRSNKYRRYRTSLRDYLNKGSG
LPDRSIMVIVAQLLEAVGYLGNQGVVHRDLKSNNILVDYEEGSDEVPHVVVADFGCAISLRGKNLQETVSK
DDLNRQGNAALMAPEVKKAYHCNSLRYHDYLKADVWAVGAIMYEVCGKKNPFYAEGIDSSKYEAGDLPQLQ
SEAVGLRVVSELLLEKNPGNRPSAQVAANILHLLLWQPSVSSFLRLSQGQSSKDSELTAWIIKVSLMQQLL
SSNHTDTALTFTPDDPGLPQNVIESSLVETFLKRVNSDSLIDASQRLVTALRPLGALAEFRGVSSTCMGTP
QLIGSVSDTVTLMA

>Strongylocentrotus_purpuratus_Pink_XP_003724360
MHRAFVDDVRVPSLSSAVSLFDVALPVRLNPMGAGADTTMYIVMKRYRTSLRDYLSMECDLPDHTVMVIVA
QLLEAVGYLGNQGVVHRDLKSNNILVDYEEASGASPHVVIADFGCALSLRGRNLQDSVSVEDRNRQGNSAL
MAPEVTKGYHHDCLHYHDYLKADAWAVGAIIYEVCGQKNPFYSDGVNSCGYEAGDLPQLQSQAVGLKIISK
LLLQKDPGERPSAQVAANILHLLLWQPSVSSLLRLCHSQSQMDSELSAWIIKVSLMQLVQDQAEKTLPSTR
ADPGIPLNPVEKGLLETFLKRVNSGHLICATQHLVKAFQPLALFAVSSLPVGREQLTDLSSNADRFVTLQA

>Homo_sapiens_PINK_NP_115785
MAVRQALGRGLQLGRALLLRFTGKPGRAYGLGRPGPAAGCVRGERPGWAAGPGAEPRRVGLGLPNRLRFFR
QSVAGLAARLQRQFVVRAWGCAGPCGRAVFLAFGLGLGLIEEKQAESRRAVSACQEIQAIFTQKSKPGPDP
LDTRRLQGFRLEEYLIGQSIGKGCSAAVYEATMPTLPQNLEVTKSTGLLPGRGPGTSAPGEGQERAPGAPA
FPLAIKMMWNISAGSSSEAILNTMSQELVPASRVALAGEYGAVTYRKSKRGPKQLAPHPNIIRVLRAFTSS
VPLLPGALVDYPDVLPSRLHPEGLGHGRTLFLVMKNYPCTLRQYLCVNTPSPRLAAMMLLQLLEGVDHLVQ
QGIAHRDLKSDNILVELDPDGCPWLVIADFGCCLADESIGLQLPFSSWYVDRGGNGCLMAPEVSTARPGPR
AVIDYSKADAWAVGAIAYEIFGLVNPFYGQGKAHLESRSYQEAQLPALPESVPPDVRQLVRALLQREASKR
PSARVAANVLHLSLWGEHILALKNLKLDKMVGWLLQQSAATLLANRLTEKCCVETKMKMLFLANLECETLC
QAALLLCSWRAAL

>Branchiostoma_belcheri_Pink_XP_019643039
MAKERLMGVKNLVERAVQLARGVAQGSRQTLHTIRHRVEQKAGLHQVPGRHPQAVTTVTTTQQQAPSFFTR
VLLPKQINSLAATLRRSAALRLLAYRSPSHNQIPIFAFVGLAYASAVSNSDKEAEEHCDSLCKDIRAIFHP
FMPKLRGSLSLLKPGKEYQLDDFALGECVGQGCNAVVYEASLKDDGNRNMEETEEQTQEPSNNNSVIPEPE
EQPATGAVNHPATCHGDCPSLAVKMVFNLEVESCSQAIMREMRREVVPARQVKVNGRIMGRDWENGNRVKK
KRLPPHPNIIEMLTVFTDTVPLLPDAMQNYPAALPARFNPEGFGRNMTLFIVMKRYPVTLKEYLETNTPSS
RVAMAMVAQLLEGVSHLVDHGIAHRDLKNDNILVELHGDCCPRLILTDFGCCLCNNDNSLQVPFPHSELDT
RDGNSALMAPEVATAVPGPRNFVDYTKSDVFAVGALAYEILGQPNPFYKSEAGTRLNTCTFKEADLPPLPS
SADGKFCNIVKLLLKRDPTQRPNARVAANMVHLLMWDGGHLTSSKDLIGWLMQLSAITLLEKVKGHKVRGQ
GSDNTEHLLKREFLCNVTVGELMEAFHLLRFC

>Danio_rerio_Pink_NP_001008628
MSVKHVLSRGLELGRSVFQLGLLKPAGRVAAKFRGERLRVSRPTRTVQPQTFLPGRYRFFRLSVSGLAAQL
QSGAFRRVIGGGSARNRAVFLAFGVGLGLIEQEQEEDRTSAALCQEIQAVFRKKKFQSLPKPFTSGYRLED
YVIGKQIGKGCNAAVYEAAAPFAPPVESKKCSLVELNQKEAEDDNKKEEPLRLSASPSFPLAMKMMWNIGA

GSSSDAILRSMSMELVPSCPQALRKEQGELTLNGHFGAVPKRLSAHPNVITVYRAFTAEVPLLPGAREEYP
DVLPTRLNPHGLGSNRTLFLVMKNYPCTLRQYLEVCVPKRTQASLMFLQLLEGVDHLCRQNIAHRDLKSDN
ILLEFDNTGCPRLVITDFGCCLAEDSGLKLPFSSWWVNRGGNSCLMAPEVSTAVPGPGVVIDYSKADVWAV
GAIAYELFGQPNPFYTLESRSYQEKQLPALPAAAPDDVQLVVKLLLRKNPHKRPSARVAANILHISLWGRR
VLMGLDKVQMAEMMAWLQCQSAVVLLKGRGRDQSSVEAELQRSFLANIELEDLRTAVSFMTYERKQWRSLL
MSNTQP

>Caenorhabditis_elegans_Pink_NP_495017
MSMKRFGKAAYRIANELVAKGGRLPIFQRFLPRIFPATYNLGVHVVLKKAPFPRQNALRIARLVTRHGRVF
RPFSSVIIERHRFQNQNDWRRKFQPIRKELPRNVDLVERIRQIFGNSLRYNEDLKSTEWPNRIDSYEFGEF
LGQGCNAAVYSARLANSDAESSGNTHYGAGFNEVTNILAEIPPVSKVAQKKFPLAIKLMFNFEHDRDGDAH
LLKSMGNELAPYPNAAKLLNGQMGTFRPLPAKHPNVVRIQTAFIDSLKVLPDAIERYPDALHTARWYESIA
SEPKTMYVVMRRYRQTLHEYVWTRHRNYWTGRVIIAQLLEACTYLHKHKVAQRDMKSDNILLEYDFDDEIP
QLVVADFGCALACDNWQVDYESDEVSLGGNAKTKAPEIATAVPGKNVKVNFEMADTWAAGGLSYEVLTRSN
PFYKLLDTATYQESELPALPSRVNFVARDVIFDLLKRDPNERVKPNIAANALNLSLFRMGEDVKQMMEKCG
ISQMTTLLAGSSKVLSQKINSRLDKVMNLITAETIMANLAPHLISRAERQLRATFLSRMNREDIWRSLQYF
FPAGVQLDTPATSSDCLETISSLMSSFSNDSENYEKQQKPAKNGYNNVPLLLRNVIRTDADGINGIVHRVR
SK

>Homo_sapiens_PRKCQ_AAA75571
MSPFLRIGLSNFDCGSCQSCQGEAVNPYCAVLVKEYVESENGQMYIQKKPTMYPPWDSTFDAHINKGRVMQ
IIVKGKNVDLISETTVELYSLAERCRKNNGKTEIWLELKPQGRMLMNARYFLEMSDTKDMNEFETEGFFAL
HQRRGAIKQAKVHHVKCHEFTATFFPQPTFCSVCHEFVWGLNKQGYQCRQCNAAIHKKCIDKVIAKCTGSA
INSRETMFHKERFKIDMPHRFKVYNYKSPTFCEHCGTLLWGLARQGLKCDACGMNVHHRCQTKVANLCGIN
QKLMAEALAMIESTQQARCLRDTEQIFREGPVEIGLPCSIKNEARLPCLPTPGKREPQGISWESPLDEVDK
MCHLPEPELNKERPSLQIKLKIEDFILHKMLGKGSFGKVFLAEFKKTNQFFAIKALKKDVVLMDDDVECTM
VEKRVLSLAWEHPFLTHMFCTFQTKENLFFVMEYLNGGDLMYHIQSCHKFDLSRATFYAAEIILGLQFLHS
KGIVYRDLKLDNILLDKDGHIKIADFGMCKENMLGDAKTNTFCGTPDYIAPEILLGQKYNHSVDWWSFGVL
LYEMLIGQSPFHGQDEEELFHSIRMDNPFYPRWLEKEAKDLLVKLFVREPEKRLGVRGDIRQHPLFREINW
EELERKEIDPPFRPKVKSPFDCSNFDKEFLNEKPRLSFADRALINSMDQNMFRNFSFMNPRMERLIS

Ripk

>Paracentrotus_lividus_Ripk
MKYMERGSLWDFRIKKWKDIPDLRPLTNRMVYQISSGMHFLHSIYIIHRDLKLENVLIDAQLIVKIADLGL
ATNLKTSSGDNCWGTDSHKPPEAFRTDLPSKTKVVTTKYDVYSFSMTLYQLLTGIHPYSDRGIDMIRLLKV
EAKQTPSLQPIPDNTTPELIEILKASWRYEANERPNFKEITNRVGDLDTDPSYESLAVYFGQPSSKVTKP

>Homo_sapiens_RIPK1_NP_001341859
MQPDMSLNVIKMKSSDFLESAELDSGGFGKVSLCFHRTQGLMIMKTVYKGPNCIEHNEALLEEAKMMNRLR
HSRVVKLLGVIIEEGKYSLVMEYMEKGNLMHVLKAEMSTPLSVKGRIILEIIEGMCYLHGKGVIHKDLKPE
NILVDNDFHIKIADLGLASFKMWSKLNNEEHNELREVDGTAKKNGGTLYYMAPEHLNDVNAKPTEKSDVYS
FAVVLWAIFANKEPYENAICEQQLIMCIKSGNRPDVDDITEYCPREIISLMKLCWEANPEARPTFPGIEEK
FRPFYLSQLEESVEEDVKSLKKEYSNENAVVKRMQSLQLDCVAVPSSRSNSATEQPGSLHSSQGLGMGPVE
ESWFAPSLEHPQEENEPSLQSKLQDEANYHLYGSRMDRQTKQQPRQNVAYNREEERRRRVSHDPFAQQRPY
ENFQNTEGKGTAYSSAASHGNAVHQPSGLTSQPQVLYQNNGLYSSHGFGTRPLDPGTAGPRVWYRPIPSHM
PSLHNIPVPETNYLGNTPTMPFSSLPPTDESIKYTIYNSTGIQIGAYNYMEIGGTSSSLLDSTNTNFKEEP
AAKYQAIFDNTTSLTDKHLDPIRENLGKHWKNCARKLGFTQSQIDEIDHDYERDGLKEKVYQMLQKWVMRE
GIKGATVGKLAQALHQCSRIDLLSSLIYVSQN

>Homo_sapiens_RIPK2_NP_003812

MNGEAICSALPTIPYHKLADLRYLSRGASGTVSSARHADWRVQVAVKHLHIHTPLLDSERKDVLREAEILH
KARFSYILPILGICNEPEFLGIVTEYMPNGSLNELLHRKTEYPDVAWPLRFRILHEIALGVNYLHNMTPPL
LHHDLKTQNILLDNEFHVKIADFGLSKWRMMSLSQSRSSKSAPEGGTIIYMPPENYEPGQKSRASIKHDIY
SYAVITWEVLSRKQPFEDVTNPLQIMYSVSQGHRPVINEESLPYDIPHRARMISLIESGWAQNPDERPSFL
KCLIELEPVLRTFEEITFLEAVIQLKKTKLQSVSSAIHLCDKKKMELSLNIPVNHGPQEESCGSSQLHENS
GSPETSRSLPAPQDNDFLSRKAQDCYFMKLHHCPGNHSWDSTISGSQRAAFCDHKTTPCSSAIINPLSTAG
NSERLQPGIAQQWIQSKREDIVNQMTEACLNQSLDALLSRDLIMKEDYELVSTKPTRTSKVRQLLDTTDIQ
GEEFAKVIVQKLKDNKQMGLQPYPEILVVSRSPSLNLLQNKSM

>Homo_sapiens_RIPK3_NP_006862.2
MSCVKLWPSGAPAPLVSIEELENQELVGKGGFGTVFRAQHRKWGYDVAVKIVNSKAISREVKAMASLDNEF
VLRLEGVIEKVNWDQDPKPALVTKFMENGSLSGLLQSQCPRPWPLLCRLLKEVVLGMFYLHDQNPVLLHRD
LKPSNVLLDPELHVKLADFGLSTFQGGSQSGTGSGEPGGTLGYLAPELFVNVNRKASTASDVYSFGILMWA
VLAGREVELPTEPSLVYEAVCNRQNRPSLAELPQAGPETPGLEGLKELMQLCWSSEPKDRPSFQECLPKTD
EVFQMVENNMNAAVSTVKDFLSQLRSSNRRFSIPESGQGGTEMDGFRRTIENQHSRNDVMVSEWLNKLNLE
EPPSSVPKKCPSLTKRSRAQEEQVPQAWTAGTSSDSMAQPPQTPETSTFRNQMPSPTSTGTPSPGPRGNQG
AERQGMNWSCRTPEPNPVTGRPLVNIYNCSGVQVGDNNYLTMQQTTALPTWGLAPSGKGRGLQHPPPVGSQ
EGPKDPEAWSRPQGWYNHSGK

>Homo_sapiens_RIPK4_NP_065690.2
MEGDGGTPWALALLRTFDAGEFTGWEKVGSGGFGQVYKVRHVHWKTWLAIKCSPSLHVDDRERMELLEEAK
KMEMAKFRYILPVYGICREPVGLVMEYMETGSLEKLLASEPLPWDLRFRIIHETAVGMNFLHCMAPPLLHL
DLKPANILLDAHYHVKISDFGLAKCNGLSHSHDLSMDGLFGTIAYLPPERIREKSRLFDTKHDVYSFAIVI
WGVLTQKKPFADEKNILHIMVKVVKGHRPELPPVCRARPRACSHLIRLMQRCWQGDPRVRPTFQEITSETE
DLCEKPDDEVKETAHDLDVKSPPEPRSEVVPARLKRASAPTFDNDYSLSELLSQLDSGVSQAVEGPEELSR
SSSESKLPSSGSGKRLSGVSSVDSAFSSRGSLSLSFEREPSTSDLGTTDVQKKKLVDAIVSGDTSKLMKIL
QPQDVDLALDSGASLLHLAVEAGQEECAKWLLLNNANPNLSNRRGSTPLHMAVERRVRGVVELLLARKISV
NAKDEDQWTALHFAAQNGDESSTRLLLEKNASVNEVDFEGRTPMHVACQHGQENIVRILLRRGVDVSLQGK
DAWLPLHYAAWQGHLPIVKLLAKQPGVSVNAQTLDGRTPLHLAAQRGHYRVARILIDLCSDVNVCSLLAQT
PLHVAAETGHTSTARLLLHRGAGKEAMTSDGYTALHLAARNGHLATVKLLVEEKADVLARGPLNQTALHLA
AAHGHSEVVEELVSADVIDLFDEQGLSALHLAAQGRHAQTVETLLRHGAHINLQSLKFQGGHGPAATLLRR
SKT

>Strongylocentrotus_purpuratus_Ripk1_XP_011682107
MGRKKKQKKCERASTPQPAKHRTERQAPRPPDVTRPEMALEAAGDEADARVSDPQVEVKLEKIMMEDLTGI
DSTGERCAFHEKKARDHGRWHLGGGSFGEVYKAVHALHGQVAVKLAKPGKEHRDHVVMYQKEAQKHENSLR
CEDIVSIKGFVQCEDDACYCGLIGIVMKYMEKGSLWDFRISEKWQARPDLWPLTNRMVYQISSGMLFLHSK
KIIHRDLKLENVLLDGNLHAKVADLGLATNLQTSFGDERCGTNSHKPPEAFRTDLPNSTKVVTPEYDVYSF
AMTLYELLTGIHPYSDRGFFMMMLLKVDSKQSPCLQPVPANTPEDLIRVMEESWSYEPNDRPNFREITDLV
ANLDTDPTHESLGYYLGEQRSSKKEGSADKESKRRGMENVTEQDQAPSRHHSNTNAAVHDTQTAIDNESVR
MEGLTLNDPTLVDQRNGNRAKPITETVTLHQMEGQESHPPHLGQDNVVAQPEIQHSQPSSQESSKPSSYGQ
LKSQTSGSQYGCHDNNFQPTVEAEQLMEFTGFVSASTENSPGPSVSQPLQSYREDNKPVPVESSHGNTEYA
VGHMAGGPIHQESLVVAASRPISTESPGVVPAQPSSTESPVEAPSQPRSIERDPTVIFDSSQPSDEDFEIK
VKVRLNIQPRASMASKLDQIFICLERCKHRDYWDSNSSLSTMFSFSDITLKSLQLPATVDDDDFTLYFRVC
NKRDLDKLHELSEKGRLRQSLYGFFVDVHLKEVCLPGEPDLKVESWEEQFNKGTAHFQKKEKANESKSDSE
SSESRQKPPNEIRSGASFTQTNCSGATQINTGKVRITMNSAPKNMVIGDQYNYYNHYRGSRGAGEGAGGHS
SGRQPQKKNQGETKLCGNCE

>Strongylocentrotus_purpuratus_Ripk4_XP_011683628
MVDLNVKVSSGVKFAFHEKKARELEGCHLGGGSFGEVFKAVHKVHGDVAVKLAKPGKGDQLKMHQKEARKH
GLSLTCDYIVAIKGFVKCEEDTCPCGLLGIVMQYMENGSLWDFRISEWQPFPDLWPLTNRMVYQISCGMHF
LHSKNIIHRDLKLENVLVDGDLHVKIADLGLATNLQTSSGDRCWGTDSHKPPEAFRTDLPNSTKVVTPKYD
VYSFAMTLYELLTGIHPYSDRGFDMMKLLKVEAKQPPCLQPVPVNAPKDLIEVMQECWSYEANERPNFREI
RDRVKNLGTEPTYASLGFYLGEKLQIETDYYRL

>Saccoglossus_kowalevskii_Ripk1_XP_006818289
MAAKDSSTLTGEGLYVIDIDKVTNEEYLDKGSFGMVYKALHDDWGEVVVKRIFENTDVSESERKEMVEEAK
KMSEVRSAHIVMLYGVIAHPSQYSLVIEYMCYGSLNNFQSKYRIPWALKAQMVHHIILGMNYLHKTAKIVH
RDLKIDNVLVGKGFQVKVADFGLAVWRTYSRKYVKKRSRRSKGTSGAGTITHIPPEHLKNVNLRTEFTFDV
YSFGITLWEMFTEHIPYRNAINSAHIFNAIENGQRPDIELVPSECPEFLKQMMEDCWHGDPVRRPSFGALK
ITIEAKWKAEFASKIHDAEMDVQNQIKIRKLKLEGTTSTGKGVYQPEDGDQMKSLTSKMTKVSFATPIRTE
EGDLAEDGDPKNNSKSDSKSSDMSYRDPVPETQPKGSYRPFSQKPATDAVAEGKDQKSQGVPPQGVSSVPP
QGVHGVPPQGIHGVPPQGVHGVPPQGVQGVPPQGVQGVPGTYTYPQVFYPQYYQAPPINVYKYGTGEGTSA
IHITGSKGVQIGNQNVMYVGSKESEQKPNMQRSKQEEKAVIKVT

>Saccoglossus_kowalevskii_Ripk2_XP_006823146
MATANPDIGHIVDEYAITNENFGGGGFGNVYKASHIDWGVVAVKRIFKNLQIIERDRKMLEDEARTMTNIR
SSYVVQLLGVVMNPSNYALVLEYMCHGSLRDYQKNNDVSWPIILDLVHDVILGMNYLHKTLKLVHRDVKID
NILVDKGPKAKVSDFGLSVWHEYSKKVSRHSRVSNTPDIDVVHSAVIISAVTRNQRPDEKEIPVECPLILK
SMMEECWNGNPYKRPTFQALKEMMEKSEDSVTGLNFDRIDINKTKLIDAAAKGDIKEVKRLLSEGYDVTFR
IRMEIQHCTMQHGIDMGKWLIYL

>Branchiostoma_belcheri_Ripk1_XP_019629185
MPNTSQDVNVLEFKSLTVGETLGRGAFGTVFKAKHKHLGVVAVKLVYSRSDLDGRNELVLRQEVTMMNQLN
CDHVVRLLGIVLEPGNYCLVVQYMRYGGLADFLTKMDIPWPLRFRMSFQIIQGMNYLHHMTPPVIHRDLKA
DNVLVDDAINVKIADLGLSTWKTWRSLSKLESRQRGEQPQHERQRIGTISHVAPECLQDPNRKQNEKSDVY
SFGIVLWEILSRQRVFGNALNSTTLAFGIVSGKRPDLQLIPPGSPSELVELMKKCWDQEPDRRPDFTGCKQ
AIEPVYDTSYEPDVDSAVIWTKQELQKMDKLVHHPGRGVYAQDSAQQDPLPSMETTVDQPPPTEPDAQDGE
PPTISQTVEAEGQGQLNEAVADLSLAHSPSESDSSLTVDAELGTPSPPPTDEKKSFPAGDRKERPATGATG
ATPVRATDQHDIRFNLQESSKYANKGEAQDCKGASAQEEGPQHPLLTSRGKGAKPITEHVQFTSPVESSEK
NTSIPTAQQYAMPPRPQAGPFYATASGPPGHGQYQPAYLPAPGGYWYTPAPGMQPTFIPQGFPMPSWPQQG
GGQGQNAPIFYQKQSSDGSTTVTMTMSNVEGVQIGDKNVMYVGGAQGPRSGGRHHPVRQGNITYRGKITES
TRKVETRDIQAVQEGIGKHWKVLARKLGFLEGKIDSFDHDFDRDGLDEKVYQMLRKWSMEWGKDATVKALA
DALCSIGKADVAALLKD

>Branchiostoma_belcheri_Ripk2_XP_019626679
MNLGSLNDFPSVTVDSGFPIIDFEQLEFPEKSLLGQGSFGSVRKAFHRNWRQDVAVKSLTFEIYGTSEQEL
LYSEARKLNLGSRSDYVIRLLGICLEPQVAIVMPYMENGCLSELLQDVDVPWALRWRMARQIALGMTFLHC
QNPQIIHCDLKAENVLLDGDFHVKISDFGLSKWKMQSRIVTETSPMGGTPTHVPPEFFDTDGGPTDKTDVY
SFSVLLWEIASRRKPYRDAEGRLNLSRPLSCYVTGGGRPDMSLVPRGVPGVDTVSQLMQACWSQCPDDRPS
FQECVDQLRDVNSMFSDEDILEAIINVRRKMKAVVK

>Branchiostoma_belcheri_Ripk3_XP_019629560
MDPQSRFSVISKDKLKIYDSSLLGSGGFGFVVKAKHVDWSMDVAVKCICSKQTMTEKQQKDLYLEAEKLEA
ARFRYVIHFYGMCIEPTFYCLIMDMAENGSLKSLLGVDIPWALRWRIAFETAIGMNYLHNLAPRILHCDLK
SENICLDEEYHVKITDFGLSKWKSITGATSIGNQGEVGGTATHIPPESWEDVNCKPDTPWDVYSYGIVLWE
ILTRRQPFEHAHNSAHIEIAVRGNQRPDQKLIPTEPQECADFSRLMRQCWSQKPDDRPSFKDCVDRIDPIY
RNYKQEVPAAIAALQSGSSSNNTAGSRSSVSSIQGKLGDLKLEDVVRHYTGRAAPQLDEAVPIQVQSAQGG
QQHQVPQSAQGGQYQPTQDPQGGQYQPTQDPQGGQYQPTQDPQGGQKYQAPPAGGAWGGGQPPWGHNIPGL
GAGANFNLQDCQNVIIGNNNVITVNNAPTQPSSAKRKNPPAQKTSAPKVSEPGRQVTNKMLQVVASHIGRD
WKKLARELDMTEPQIDQIQYDYHHEGLQEITYQMMIKWKQQNGKRATVGKLAQAFSNIDKGYLAKHL

>Ciona_robusta_Ripk1_XP_002119844.2
MASPTDIAKFNYKDFSEKKILGEGSFNQVYQVYNPYIGNCVVKIVSRCFYGQEREKNLLLKEAADMQRVRH
DHLLPLLGVVMEPEHLCLVFDVADNGSLLHALQRIQIPWSIKNMNAYQVILAVCFLHEQDDQIVHRNLKTS
NVLLDAEFNAKVCDFVLPNRFQWISQATQQRDRTGRYRRQGPTKQRNGSEIAFLAPEAIKNINAKPDPAFD
VYSYGGILKELLTGKQPFTDKTTKEVFEYIANGNSMPIGELLPGHPPELVELMRETLKSEPHDRPLSSEVL
RRIQIIHETTKHEVPQDLKTCKQSYKKYTVVRKLRQPPQGQLKTFEENNTLNKPSNERNVVGQVMASSAKP
ATSSSSGIQYGAQPEGESEEDETKPQSYIGHAQDLGVGAQPIPQYSSSLGAAMSPPPGASMSPPLGAQGRQ

TITTENIGGSTSIRIDVNTNHNGPVTPTPQDFTIVRALSSNRILSKTDMIFVASKMDNRFKLFARMMELED
SVIEHVRYNNEKYGLEEIVYQLIQRWVRGKGGQVLVQNMAQVLVDAGYQHIAQLLEP

>Ciona_robusta_Ripk2_XP_002128565
MDNCIKLSELKVDPIALAPIGQGSYGTVYEAWHNRHHSVAVKICRIHMDEQNFQEEKELNKRATDENACVP
YFGFISEYYRGLLYNGLVFKFMECGSLGDLLHRHNVYPDNCLAFRMLHEIAVAMNKLHEINADQRLLHCDL
KPCNILLDIDLHAHITDFGAARFSTVTENRDRVIKQDPNSMRTIAYCAPEFFSDNFRRTSKYDVYSFSILA
WELLAREIPYRGQSNNDIPPRVLANCRPDLNLLRGTIDNYEAFEHFLRRCWAQNSNDRPTFLDIATELKPL
THTDEEVLQHAVMQAKQELLTKLNRLSFQRSLENYRYNFTEVFTGHHPTAEQSKRMEIGDNLAAQPDNNQH
IAQNENDQLSVGDRNLTDEDCRRLGKNYARSWETLASDLGISFAQIDNIKQDHRRNEERKIAVFQKWKEID
PNPTVRNLIQKLRQIKVDIDCYSFLDPDRVIRDDLRNEGA

>Danio_rerio_Ripk1_NP_001036815
MSTSVSPSLMKSADLIKKEPLDYGGFGEVYLCYHKTLGHVVLKTVYTGPPRSGRQKQSLLEEGSLMSRLNH
QRVVKLLGVILEDGDYSLVMELIPKGNLLTMLEKVTVPISVKGRIILEILEGMVYLTKNQVIHKDLKPENI
LVDKNFHIKIADLGLATSEVWSKLTKEESRRQSRLGKKTSAHAAGTLCYMAPEHLKSIHTRSSEKSDVYSF
AIVLWVILTGREPYEDARSEDQICHCVCQGERPDEALIPPNTPTDITDLMKSCWHQDPLQRPSFTDCYNRF
LPFYKEKLAANVKADLENLMKLYEGPEELVEKLKLLNTNALIPEDPSLCSRDSPTPLRSSDIGPVEASIED
LSFRSCEDSVLEADATPSCPNLELKLEQEYNYHKFGSRIVDQSDGSMWSPAQQPASSTDASSQMSSVKSWT
KPSAPSTEEELYPRAAASFDSLHRPELRSQFSVPQSDHERLRPHAHLPWNRVKSCPEGANHVAESLIDTNS
NLRFNSPVTYPASDSAFPVSISNASAIQIGSYNTMNLRISDSSPYSSLSTTGSTTSRYKELLLMYESHTVT
ESHLELVRQNVGANWKQVARKLGLSEIDVETIEHDYDRDGLTEKVHQMLERWKMREGLMGITVGKLCQALE
GCIKPNALLHMLRQLQDSAAAP

>Danio_rerio_Ripk2_NP_919392.2
MSRNMEHTGCVNICSLTSTLPVIPYRKLTDLHYISKGGFGTVFRAQHSDWRTTVAIKCLKLDSPVGERERN
CLLKEAEVLHKARFNHIIQIFGVCNEPEFFCIITEYMTNGSLDELLHEKDIYPAVAWPLRLRILYEIALGV
NFLHNMSPPLLHHDLKTQNILMDGEYHVKIADFGLSKWRQLSITKGSGSKPAEMGGTVIYMPPEEYEPSKT
RRTDVKYDMYSYAIIMWEVLSRRIPFEEATNPMQIMFSVLRGARPDTGLDSLPVDIPSRETLINLMTSGWT
ANPDERPSFLHCLIELEPMLRRFDEIDVLEAVLEVKRIKYRRPSSCCSSTQSNGKKIEEKCVKELNVPWPD
NSSTSGSGSCSSQEAEISQPGFLTISTPSQGAYAGLPSSLMSLPLDPPKPLMDNCSPNNLSPEYQTAQVVS
DLNIPFKAHAPHSESELALAIQPLTLHPHPQDFVTAFDDQGPAARWIAARREEVVRQMTEACLNQSLDALL
SRELLMREDYELVVNQTTRTAKVRKLLDTCERHSEEFCRVVVRKLQDNKQMGLQPYPDLSTTSSPPSPYVA
PSAPFLSNSFNNTRNF

>Danio_rerio_Ripk3_XP_001343827
MDGGEMRFRVSVVRDDCLDRFLRVGSGGFGQIFRARHTLWGTDVAVKLLHYKEGSASSAQREAELMFDAGN
SNVVRVLGVYEGRLGDPQRPLQCGLVLEFLARGSLEDLLQRLAAPPPCALALRMALQVSLGMNFLHQLTPP
ILHLDLKPSNVLLSDSLDAKITDFGLSRVAENVCKYTGVNDEDEGGTLSYMPPEALQSSSYKPSKAFDVYS
YGILLWSIITGKEPYSGVQSSLVRFRIPLGDRPDLASVDCSETEGLDELLKLMMQCWDQEPHRRPSFLDCV
HAITAIYTMHARRLNDDVHDVLEQLQDDEICSSLRSVQISRKPHREVDPQDLSCVTGPAPQQETAAAYTET
VQKECSPRAAPPVGAIRKTQRQTSNPAVQVSMSNVFGVQIGNNNSMNITQKPRQRHRTAPSAVNTHSSHPQ
NPPQKHQ

>Danio_rerio_Ripk4_NP_998243
MDVPENSPGIMGLLKTFDASEFGSWEKIGSGGFGQVYKVRHMQWKTWLAIKCPPSLHSDDKERAELLEEAK
KMEAAKFRYILPVYGVCSDPQGLVMEYMETGSLETLLATEPLPWELRFRIIHETAVGMNFLHCMNPPLLHL
DLKPANILLDAHYHIKISDFGLARWNGFARDDDISRDGFCGTIAYLPPERIIEKDRVSDTKHDVYSFSIVI
WGILTQKKPYQGENNILHIMVKVVKGVRPDLSLIPRSRPQACSGFLSLMQKCWAQSPQARPSFEEITSEAE
ELCTKPHEESRASVSSEPECSPCPAPASSEQTNDQKPVRPKSAMLPEKDYSLSELLTQIDSGFSRSLSNVQ
EESLESKDNTSKRLSGISSVDSAFSSQGSITLSFDKENAVNDSSELQKKKLCDAIRTEDIAKLMKILQPQD
VDLLLDGGSNLLHYAVSLANEEAVKFLLLSNCNPNLANAQGATPLHQAAEKRLKGVSEILLSRKTTNVNAK
DEDQYTPLHFAAQNGDEALTRLLLDRSASINETDAQGRTPTHIACHHGQENVVRVLLSRGADVHVKGKDDW
TALHLAAWKGHLGIVKLLVKQAGADVDGQTSDGRSPLHLASQRGQYRVARILVELGANVHLTSDDLYAPLH
VAAETGHTSTSRLLVKHDADIKSRTANGCTALHLASQKGHLPTVKMLLAEGADPESVNHDLRTPCHLAAQN

GHCEVLKELLRSCSDVANAQDRNGLTALHLAVSGGHKDAICVLLEGGADAAPLTPQPVGDRSFDYTSESEP
ESPNPLTPTRKVVILKLTNRKNSEHTLIPVDARCESAPC

>Homo_sapiens_MLKL_NP_689862
MENLKHIITLGQVIHKRCEEMKYCKKQCRRLGHRVLGLIKPLEMLQDQGKRSVPSEKLTTAMNRFKAALEE
ANGEIEKFSNRSNICRFLTASQDKILFKDVNRKLSDVWKELSLLLQVEQRMPVSPISQGASWAQEDQQDAD
EDRRAFQMLRRDNEKIEASLRRLEINMKEIKETLRQYLPPKCMQEIPQEQIKEIKKEQLSGSPWILLRENE
VSTLYKGEYHRAPVAIKVFKKLQAGSIAIVRQTFNKEIKTMKKFESPNILRIFGICIDETVTPPQFSIVME
YCELGTLRELLDREKDLTLGKRMVLVLGAARGLYRLHHSEAPELHGKIRSSNFLVTQGYQVKLAGFELRKT
QTSMSLGTTREKTDRVKSTAYLSPQELEDVFYQYDVKSEIYSFGIVLWEIATGDIPFQGCNSEKIRKLVAV
KRQQEPLGEDCPSELREIIDECRAHDPSVRPSVDEILKKLSTFSK

Ulk

>Paracentrotus_lividus_Ulk1/2
MEYVDEYEYNKKDIIGHGAFAIVFRGRERKRPDQTVAIKCINKKNLSKSQTFPEKEIEILKELHHENVVSL
LHFKETASSLFMVMEFCNGGDLADYLHIKGTLSEDTIRFFLGQIAAAMKAIHEKGILHRDLKPQNLLLSHN
SRNKVPHPKEIHLKIADFGFARFLESDMMAATLCGSPLYMAPEVITSKHYDAKADLWSIGTIIFQCLTGSA
PFKAPNPPELKKLYLKARTLDPNIPPGTSRALKDLLIRLLKRNQKDRIEFDEFFNHDFLSKTLKSTSTSPM
PVPGRTYSFSSDSPGERRSLSVSPLSGHMPISSPEEPSPSRATRDYSPATPSRPSAAKGLQDKLSSGMGSS
DLVEDDFVIVQPGLVSELSYETSGASVNVQTTTDVITIRSHSSPIMSSSRCQPRPTTSPGSTATTTVRRQL
PSPSERPSSLPISSSPSTSPNSGRHRVSPKQSPSSLISPSKIHAQTGGMEYKPPSPVQATGLSSPGSQYNP
GVVHKFYKFHASPTSSSPSPPRIPRVSTEPSMSPQGAGLYRGSSPQGTSPTNIPSPARRKLSSPARGSPQF
FTGPSSLPTIAGSPTKKGFGNEITFTIGSHGISPSEPLNMPFAKSRRVRASSCCLDGQDMSDSPGRDAIIP
RSASSSRLSEPLCLKAAFDNLAMNPAGSIEGIPGAIGASPPTHPTSFFIGSQSRRSSVLTEGSPSSQGSLT
FATSPPNMEGPISFVAPELPEETLLAPEHTETVDRLNVILGIVEAIVEVAKSRSIPLAESVYNQGSSIFSN
SQVCFVSENYRLAEQLVLYTRSLELLNAALTMAKEEFSAGRLKPSNAVRMVLQELNRVYHLCLTKSRQFCE
GSPLQSLDIDLNSAMITADKLMYSYAIEQCQSAGMDEMFGNSQECLQRYRTAQMLLHGLCLQAGTDHDRNL
LLKFKNALDQRLLFLERQQTPVTPMIGHL

>Paracentrotus_lividus_Ulk3
MAASLSSRIPKLPGFVFTEKLGSGTYATVYKAYRKGQQREVVAIKVVSKESLNKLSTENLLQEIEILKKIK
HEYIVELKDFQWDTHYIYLIMEFCSGGDLSQTISKRIALPEATVKTFLRQLACALKFLYSRNITHMDLKPQ
NLLLSNSYNPVLKMADFGFAQHITEDVQTDMLRGSPLYMAPEIITDRIYNAKADLWSAGVIMFECLFGSAP
LASSSYAQLAEKIRSPKPIEIPTFVQTSDSCRDLLSRLLKRDPGERIEFEDFFSHPFIDLEHIPCSESLDK
ARATVIEAVKADQKGEWKEAIRLYLKAMEYFIPAIQYERDASKKDSLRVRSLEYMRRAEELKGLLKPQKTS
LTECNSSQDNAEQTSSSSTESGLVHDIHLLDKMANGYPEMKVAVKLTRDAIREDSNEEYQIALDLYEQALN
QLMPLLQVEQKGKRRDLLLKETEKCMSRAEVLKQYIEIEKMRPMRQSSLDHRLQGPDHIKDSCVLQ

>Homo_sapiens_Ulk1_NP_003556
MEPGRGGTETVGKFEFSRKDLIGHGAFAVVFKGRHREKHDLEVAVKCINKKNLAKSQTLLGKEIKILKELK
HENIVALYDFQEMANSVYLVMEYCNGGDLADYLHAMRTLSEDTIRLFLQQIAGAMRLLHSKGIIHRDLKPQ
NILLSNPAGRRANPNSIRVKIADFGFARYLQSNMMAATLCGSPMYMAPEVIMSQHYDGKADLWSIGTIVYQ
CLTGKAPFQASSPQDLRLFYEKNKTLVPTIPRETSAPLRQLLLALLQRNHKDRMDFDEFFHHPFLDASPSV
RKSPPVPVPSYPSSGSGSSSSSSSTSHLASPPSLGEMQQLQKTLASPADTAGFLHSSRDSGGSKDSSCDTD
DFVMVPAQFPGDLVAEAPSAKPPPDSLMCSGSSLVASAGLESHGRTPSPSPPCSSSPSPSGRAGPFSSSRC
GASVPIPVPTQVQNYQRIERNLQSPTQFQTPRSSAIRRSGSTSPLGFARASPSPPAHAEHGGVLARKMSLG
GGRPYTPSPQVGTIPERPGWSGTPSPQGAEMRGGRSPRPGSSAPEHSPRTSGLGCRLHSAPNLSDLHVVRP
KLPKPPTDPLGAVFSPPQASPPQPSHGLQSCRNLRGSPKLPDFLQRNPLPPILGSPTKAVPSFDFPKTPSS
QNLLALLARQGVVMTPPRNRTLPDLSEVGPFHGQPLGPGLRPGEDPKGPFGRSFSTSRLTDLLLKAAFGTQ
APDPGSTESLQEKPMEIAPSAGFGGSLHPGARAGGTSSPSPVVFTVGSPPSGSTPPQGPRTRMFSAGPTGS
ASSSARHLVPGPCSEAPAPELPAPGHGCSFADPIAANLEGAVTFEAPDLPEETLMEQEHTEILRGLRFTLL

FVQHVLEIAALKGSASEAAGGPEYQLQESVVADQISLLSREWGFAEQLVLYLKVAELLSSGLQSAIDQIRA
GKLCLSSTVKQVVRRLNELYKASVVSCQGLSLRLQRFFLDKQRLLDRIHSITAERLIFSHAVQMVQSAALD
EMFQHREGCVPRYHKALLLLEGLQHMLSDQADIENVTKCKLCIERRLSALLTGICA

>Homo_sapiens_ULK2_NP_055498.3
MEVVGDFEYSKRDLVGHGAFAVVFRGRHRQKTDWEVAIKSINKKNLSKSQILLGKEIKILKELQHENIVAL
YDVQELPNSVFLVMEYCNGGDLADYLQAKGTLSEDTIRVFLHQIAAAMRILHSKGIIHRDLKPQNILLSYA
NRRKSSVSGIRIKIADFGFARYLHSNMMAATLCGSPMYMAPEVIMSQHYDAKADLWSIGTVIYQCLVGKPP
FQANSPQDLRMFYEKNRSLMPSIPRETSPYLANLLLGLLQRNQKDRMDFEAFFSHPFLEQGPVKKSCPVPV
PMYSGSVSGSSCGSSPSCRFASPPSLPDMQHIQEENLSSPPLGPPNYLQVSKDSASTSSKNSSCDTDDFVL
VPHNISSDHSCDMPVGTAGRRASNEFLVCGGQCQPTVSPHSETAPIPVPTQIRNYQRIEQNLTSTASSGTN
VHGSPRSAVVRRSNTSPMGFLRPGSCSPVPADTAQTVGRRLSTGSSRPYSPSPLVGTIPEQFSQCCCGHPQ
GHDSRSRNSSGSPVPQAQSPQSLLSGARLQSAPTLTDIYQNKQKLRKQHSDPVCPSHTGAGYSYSPQPSRP
GSLGTSPTKHLGSSPRSSDWFFKTPLPTIIGSPTKTTAPFKIPKTQASSNLLALVTRHGPAEEQSKDGNEP
RECAHCLLVQGSERQRAEQQSKAVFGRSVSTGKLSDQQGKTPICRHQGSTDSLNTERPMDIAPAGACGGVL
APPAGTAASSKAVLFTVGSPPHSAAAPTCTHMFLRTRTTSVGPSNSGGSLCAMSGRVCVGSPPGPGFGSSP
PGAEAAPSLRYVPYGASPPSLEGLITFEAPELPEETLMEREHTDTLRHLNVMLMFTECVLDLTAMRGGNPE
LCTSAVSLYQIQESVVVDQISQLSKDWGRVEQLVLYMKAAQLLAASLHLAKAQIKSGKLSPSTAVKQVVKN
LNERYKFCITMCKKLTEKLNRFFSDKQRFIDEINSVTAEKLIYNCAVEMVQSAALDEMFQQTEDIVYRYHK
AALLLEGLSRILQDPADIENVHKYKCSIERRLSALCHSTATV

>Homo_sapiens_ULK3_NP_001092906.3
MAGPGWGPPRLDGFILTERLGSGTYATVYKAYAKKDTREVVAIKCVAKKSLNKASVENLLTEIEILKGIRH
PHIVQLKDFQWDSDNIYLIMEFCAGGDLSRFIHTRRILPEKVARVFMQQLASALQFLHERNISHLDLKPQN
ILLSSLEKPHLKLADFGFAQHMSPWDEKHVLRGSPLYMAPEMVCQRQYDARVDLWSMGVILYEALFGQPPF
ASRSFSELEEKIRSNRVIELPLRPLLSRDCRDLLQRLLERDPSRRISFQDFFAHPWVDLEHMPSGESLGRA
TALVVQAVKKDQEGDSAAALSLYCKALDFFVPALHYEVDAQRKEAIKAKVGQYVSRAEELKAIVSSSNQAL
LRQGTSARDLLREMARDKPRLLAALEVASAAMAKEEAAGGEQDALDLYQHSLGELLLLLAAEPPGRRRELL
HTEVQNLMARAEYLKEQVKMRESRWEADTLDKEGLSESVRSSCTLQ

>Homo_sapiens_ULK4_NP_060356.2
MENFILYEEIGRGSKTVVYKGRRKGTINFVAILCTDKCKRPEITNWVRLTREIKHKNIVTFHEWYETSNHL
WLVVELCTGGSLKTVIAQDENLPEDVVREFGIDLISGLHHLHKLGILFCDISPRKILLEGPGTLKFSNFCL
AKVEGENLEEFFALVAAEEGGGDNGENVLKKSMKSRVKGSPVYTAPEVVRGADFSISSDLWSLGCLLYEMF
SGKPPFFSESISELTEKILCEDPLPPIPKDSSRPKASSDFINLLDGLLQRDPQKRLTWTRLLQHSFWKKAF
AGADQESSVEDLSLSRNTMECSGPQDSKELLQNSQSRQAKGHKSGQPLGHSFRLENPTEFRPKSTLEGQLN
ESMFLLSSRPTPRTSTAVEVSPGEDMTHCSPQKTSPLTKITSGHLSQQDLESQMRELIYTDSDLVVTPIID
NPKIMKQPPVKFDAKILHLPTYSVDKLLFLKDQDWNDFLQQVCSQIDSTEKSMGASRAKLNLLCYLCVVAG
HQEVATRLLHSPLFQLLIQHLRIAPNWDIRAKVAHVIGLLASHTAELQENTPVVEAIVLLTELIRENFRNS
KLKQCLLPTLGELIYLVATQEEKKKNPRECWAVPLAAYTVLMRCLREGEERVVNHMAAKIIENVCTTFSAQ
SQGFITGEIGPILWYLFRHSTADSLRITAVSALCRITRHSPTAFQNVIEKVGLNSVINSLASAICKVQQYM
LTLFAAMLSCGIHLQRLIQEKGFVSTIIRLLDSPSTCIRAKAFLVLLYILIYNREMLLLSCQARLVMYIER
DSRKTTPGKEQQSGNEYLSKCLDLLICHIVQELPRILGDILNSLANVSGRKHPSTVQVKQLKLCLPLMPVV
LHLVTSQVFRPQVVTEEFLFSYGTILSHIKSVDSGETNIDGAIGLTASEEFIKITLSAFEAIIQYPILLKD
YRSTVVDYILPPLVSLVQSQNVEWRLFSLRLLSETTSLLVNQEFGDGKEKASVDSDSNLLALIRDVLLPQY
EHILLEPDPVPAYALKLLVAMTEHNPTFTRLVEESKLIPLIFEVTLEHQESILGNTMQSVIALLSNLVACK
DSNMELLYEQGLVSHICNLLTETATLCLDVDNKNNNEMAAPLLFSLLDILHSMLTYTSGIVRLALQAQKSG
SGEDPQAAEDLLLLNRPLTDLISLLIPLLPNEDPEIFDVSSKCLSILVQLYGGENPDSLSPENVEIFAHLL
TSKEDPKEQKLLLRILRRMITSNEKHLESLKNAGSLLRALERLAPGSGSFADSAVAPLALEILQAVGH

>Strongylocentrotus_purpuratus_Ulk1_XP_011676912
MEHIDEYEYNKKDIIGHGAFAIVFRGRERKRPDQTVAIKCINKKNLSKSQTFPEKEIEILKELHHGNVVSL
LHFKETTSSLFMVMEFCNGGDLADYLHIKGTLSEDTIRFFLGQIACAMKAIHEKGIIHRDLKPQNLLLSHN
SKHKVPHPNEIHLKIADFGFARFLEGDMMAATLCGSPLYMAPEVITSQHYDAKADLWSIGTIIFQCLTGSA
PFKAANPPELKKLYMKARTLDPNIPPGTSKALKDLLIRLLKRNQKDRIEFDKFFSHDFLGKNLKSTSTSPM

PVPSRTYSFSSDSPGERRSLSVSPLSGHMPISSPEEPSPSSVGCGPRGYSISPLAAPPLISTDRPSAAKGL
QEKLRLSSGMGSSDLVEDDFVIVQPSIVSELSYETSGASINVQTTTDVITIRSNSSPIMSSSRGHSAQPKS
STSPVSGRIMAAVVRRKLPSPSERPSSLPISSSPSTSPNTGRHRVSPKQSPSSLISPSRIHAQAGGVDNKP
PSPVQVTGLSSPGSQYNPGVVHKFYKFHASPTSPSPSPPHIPRVSTDPSMCSQGAGLYRGSSPQSQGSGTS
PTNIPSPARRKLSSPARSSPQFFTGPSSLPTIAGSPTKKGFGNEITFTIGTHGISPSEPLNMPFAKSRRVR
ASSCCLEGDGQQDISDSPGRDALIPRSASSSRLSEPLCLKAAFDNLAMNPGSSIEGIPGAIAASPPMHPTS
FFIGSQSRRNSVLTEGSPSSQGSLTFATSPPNMEGPISFVAPELPEETLLAAEHTETVDRLNVILGIVEAI
VEVAKSRSVPLAESIYNQGSSIFSNSQVCFVSENYRLAEQLVLYTRSLELLNAALTMAKEEFSAARLKPSN
AVRTVLQELNRVYHLCLIKSRQLCEGSPLQSLDIDLNSAMITADKLMYSYAIEQCQSAGMDEMFGNTQECL
QRYRTAQMLLHGLCLQAGTDHDRNLLLKFKNALDQRLLFLERQQTPVTPMIGL

>Strongylocentrotus_purpuratus_Ulk3_XP_790989.3
MAAPDAYGIPRLPGFVFTEKLGSGTYATVYKAYRKSQQREVVAVKCVSKKSLNKLSTENLLQEIEILKKIK
HEYIVELKDFQWDQHYIYLIMEFCSGGDLSQTIHKRIALPEATVKTFLRQLASALMFLNSRNITHMDLKPQ
NLLLSNSYNPVLKVADFGFAQHITEDIQADMLRGSPLYMAPEIITDRIYNAKADLWSVGVIMFECLFGGPP
LASSSYAQLAEKIRSPKPIEIPTFVESSGACRDLLSRLLKRDPGERIEFEDFFHHPFIDLEHIPCSESLDK
ARTTVMEAVKSDQKGEWKEAIRLYLKAMEYFIPAIQYERDATKKEALKVRGMEYMRRAEELKGLLKPKKTS
LNNSESVQDDSEQASRSSTDSGLVHDIHLLDKMANGNPEMKVAVKLAKDASREDNNEEYHIALDLYEQALS
HLMPILQVEQKGKRKDLLLKETEKCMTRAEVLKQYIEIQKMKPIRQSSLDRHLQATESFKDSCVLQ

>Strongylocentrotus_purpuratus_Ulk4_XP_790944.3
MENFVLYDEIRQTETSTMYKGRRKGSVNFVAIHCVDKARRSEVTNRVRLTHELSHSNIVEFYEWYETTNHL
WLIVELCTGGSLELILSQDGHFPETAVRGFGLDLVRGLHHIHSVGMLFSDFKPSKILVDSSGSLKYDDFSL
ARLEEENLDDIFDMTSAEGTSDQTKQGGEGTATSTLGCPNYLAPEVLQGQPHSIATDLWSLGCLLYEMYTG
NPPFQADEFPKLVEQILTKDYPTPKVKGPRLATKPTPDFCSLIKVLLTKDPARRATWHELVIHPFWQGQLQ
VDDGTKKSLGTTADDMDKSRTSFNARTPHEMKNVTLVTLSTEGRPVSSLKIPDNLMKASLHSNRPESRPQS
SPDTAKKDPHFLLSSRPRTSPAVPPEEPPEEPPPTKKKTGKRRKSDGAIKSRSRYQQERKISHEDSLDVDV
NIFTQWDLSVSPIIDNPKIQKPASLKYDTRSLGIPVHSNDKLLKMTDTALQIYLGNLTEALASKSGDKSAG
TQRTKLQLLNYVGTICGNGAVAKAVAVSKLIPCMINLLKTSQPLEIRCRSGRMLGLLAQNLDELPEDLPLT
EVVTALTEVLRDNNRNSKLKQSMVPAIGELLFLAATHDQDEASNPSTVPALVYTMISRCLREGEDAIVQHF
AAKVVENVAATNSVHGEKLSTNEIGHLLWQLFTHSMADTPKICAISALCRICRHSFGIFQSILDKVGLPKV
LEGLAVNISRVQQALVTMFGHLLTEGSHVQRTTQDKEFCLHVMKLVESPSVLIRAKALLVVLEIIKVSPEM
IMACCQARLITYMERDIRQQMTRGKGEGQAQWKYMDQCLTLLSDHLVNLLPQILGDAESSLAAVSGRKHPS
TIQAKQLKGSLPLVSLALHLITSQVFRPRIVGERFLSNIGALLTHVKSMDCGETNIEPAIGMLGIVEFNNI
ILSILEAVTQHPSILMEHHVEVIDSILNPLAALVASRNDETRMFCVRLFAEIMCLYLNHDNLTENGAITNP
QLLTDITSQILPSLQQLLLDADPLPLYGLKLILALIETSPDFIRKMEELELVPVLFQVLIDHRNSPTSSTI
RYVISVLNCLVADTNADMEGLYDQGIQDHISILLEDVERMYLSSEDTKVDSKALTLLMIGLLDLLHTLLKH
IACIVRNVLQAKKSGGSADTQGAERILMANKPVVSGSLIIIRLLCHKDKEIHTSACECLSLLGQLYGGEYP
NSFSADNSQAMAKALQTFDTKRQKLVLRYIKRVVSTETHHIEQLNNEGAILVEALGRMRQTASSHADVAVS
SLAADILRVVGVQ

>Branchiostoma_belcheri_Ulk1/2_XP_019618852
MAESVGEFEYHKKDLIGHGAFAIVFKGRRKKRPESPVAIKCISKKNLSKSHALLGKEIKILKELQHEHIVS
LLDCVETHTNVYLVMEYCNGGDLADYLQAKGTLSEDTIRLFFKQIASAMKALHAKGIIHRDLKPQNLLLSH
SRPNPAPQDIKIKIADFGFARYLQSNMMAATLCGSPMYMAPEVITSQHYDAKADLWSIGTIMFQCLTGKAP
FQASSPQGLKHFYERNKVLIPNLPAGTSSALRDLLTKLLKRNHKERMDYEEFFVHPFICGSPTKPTSPVPV
PLRHTSSSSSPSSKSSASPAPLPMGSSPLTAAGNLRPRETPDLLSSPQEPPSFLDVSRDTQSSKSSSDQED
FVMVPTNLPSDISAEGHLSRPRGLESAYSASPPRVSATPPSRAQAPVAVRTDSRPSSLPVHNPVHPPTGSM
AEPVPVPSQLEAYHVIQQRRGSLPSPNTPPSPARRGCGTSPGGQYSSPKLAAPGVHRDKVKSTTPPQLGTA
LLNMNPTTLPTIVGSPTSKFPLTTADSPAAAAMVVQTTPGQSMRDGAVVSQLQGSRVRAITPSSIAEWSKS
PLFKGQGSRTGMGRRQLSFGNASSLLRAAFGSPSGSTGGASSSGSMEVSVPVRSQGATGVSPPSSMSPPSG
GAMTQFRRPSTPGAIMASSPDMEGPISFVAPELPEETLLEPAHNETLQRIHFALILGECIMEVAQSKGAPG
ASLTESLQVKQNEDFQSDQICFVSEGVRRAEQIVLYMRATQLFSSALQHAREEVRASQLKPSKAVKERVAK
LNDRYKECIRACKTLHEQGDVKMAEISASMANMTADKLLYNHAVECCQGAALDELFGNPGECASRYRTALI
LLHGLALTIEDEDDRGMLLKYSDAVDKRLTCLEKVHQPVLVFD

```
>Branchiostoma_belcheri_Ulk3_XP_019647373
MSRPGTARPGSARLGSARLAAVPQLPDFVLTERLGSGTYATVFKAYSKSKRRQVVAIKCIQKSNLNKAATD
NLLTEIEILKNVRHPHIVELKDFQWDRNNIYLIMEYCSGGDLSRFIHSKRTLPEYLAKRFGQQLAMALQFL
RSKNISHMDLKPQNILLSSRDNPVLKLADFGFAQYMGDEARMTSLRGSPLYMAPEMFCNTKYDARVDLWSL
GVILYEALFGRAPFYSRSYAELEVKIRDTRPVEIPQGIQISGKCRDLLLGLLQRDPNQRITFEEFFNHPFI
DLEHVPSHDSLDKAVAIVTEAVKYDEEGSHAEAVKKYCDALEYFVPAVHYETDESKKDVLRKRVMEYMARA
EELKSMIKPAEEEEPTNEDGEDDGREATPKEAETLWEMAGSCTRLHQALKRAAIAELRAEHELYDSALEEY
KHALEELLSLLEAEPKGRRRDLLHMEVEKLLNHAQAVKDYKNMMKRDAPRLSDSGERYNDSRDDMNRCVVQ

>Branchiostoma_belcheri_Ulk4_XP_019636504
MENFVLYDEVGRGTKSVVYKGRRKGTINFLAIHCVDKSKRAEVTNHVRLTHEIDHSNIVRFHEWYETSNHL
WLVVELCTGGSLETLLAQDGSLPEDVIRQFGVEIIRGLHHIHEMGVLFCDLNPRKILLDGPGVLKLSNFSL
ARVEEEDLEEFFRQTSAEYEQSEDHGDASDDSTPKKRRTLGSPVYMAPEILQGDGHSMQGDLWSLGCLLYE
LFTGNPPFFADNFTELADQILNKDFPTPRVRGIIRPSPDFLTLLVGLLKKEPQERLDWEGLLSHPFWQGAF
LARTGSADEKDGEKEDQTETSPEGSLQRHKNIMLSPLGKSEEGSPRKSMIASLKLDDTGELLVPQNAPDVK
EGVFTLSSTHKSAMIDQEDQEKLAATARPQTPKDAKNKNKGRESSASNVTRSTRKTSVCEDNEEVLELAYH
DSDWTVTPIVDNPKIQKPAPLKWESKGLPVPAHSADKLLKMKTVEARAHLSACLEYIRAPDKGAAGHKAKL
QLLNYLTAIASKDGVANMVIKMNMLTSLAQVMKTTTSLDLRQKLARMIGVSANHSTVVHDDAQLSEIFTAL
AEVIRDNFRNARLKQSLLPALGELMFLVSSLEEQKGGAVENWTISPLCFTLVMRCLREGEDAVVHHIAAKI
VENMATTSGAHRERFVTPEIGPMLWYLFTHSTVDALRVTAVSAMCRLSKHSSTVFQSVIDKVGLPHVLESL
VLGISRVQQSVVTMFITMLASGGHLQRLVQDKDFVQKLMRLLESPSLIIRAKAFLAIHEVIRNNREMLLTC
CQARLVMYIERDMRRTLPAKPDQQEDIEYMMRCLDLLNGLFVAELPQVMGEAISSLEAISGRKHPSSTQAK
QLKLHVPLLPVMLHLATSQVFRPKIVTEQFMSGIGTLLTHLQSLDSGATSIETALGPSAMEDLMHVVLSVL
EALAQHPTILTQYHTVVVQKILPPLAALVSSENANTRALCLRMLSEIASLYFINDDFYDMGATSDQTAANN
SQLNLIVVKMLFPQYEQLLLEPDPLPCYALKLLQALLERSPVFVRNLEEMGLVHVLFQVIIEHQDNATSST
MQSLMGILNCIVGYKDTNMQTLYDLGLVDQLMSMLLEVTSQCMESEEKGDVKAPIASLLSLLDTLHCLLKY
ITNLIRQALQGQKSGEQTQAAEHLLVVNKPLVDLTSLLIQLLCHDDPDIKDWASRCLSLMVQLYGGEYADT
FSPECVECFAEALTTADARRQKLLLRIIKRVIVTEKKHAAVLREQGQPLVDAMTSMVKVASSHADVAVSSL
AADILKTIGVKL

>Ciona_robusta_Ulk1_XP_002124150
MNGPNSNTWMVTMESIGEYEYNKADHIGHGAFALVYKGRHKVKKHHEVAIKCIDKKKVGRAQTVLDKEIRI
LKELQHENIVQLYECKESSSSVFLVMEYCNGGDLAEYLQAKGTLSEDTIRMFLQQIVSAMAAIHSKGILHR
DLKPQNLLLSHKVPNPRPQDITLKIADFGFARYLQSNAMAATLCGSPMYMAPEVITSQHYDAKADLWSIGT
IVYQCLVGKAPFQASTPQELRNFYERNRQMIPKIPSGTSPALKDLLLKLLQKRIQDRIGFKSFFNHPFLAM
GSKQKSSAPVPVPTRQTAYETSPISSNNSDPRSIIPLSPRFNHGVSPNDDGSMLTMSIASSPDSSKGSEDF
VMVSHLVSSSGSYGGEPNNNNHSTKDRKSSRSKLSGSPRGASSPILSYGRRRSIQSPPLSSSPVMRRANQV
RRQSGTSISGQTSQLESSKSKPTSKPSAMPIPVPTQVENYEKIERRNSQASMESLTHHARSGSSHSLESSS
PFQTGISPSSISPKLDPSPTQGRHIRRLSGVSRAVEFSTIMENPTDESCNSSIPSNQKSVISNSSPNSSLS
KAQTVPDLVRYGKLHRENSLLRCQSSGKLSEQVMRMLFNSKQNAFFGYTPANRLAVCRLSMDKLSIQADTS
SSITPPNSPTDQTELRKYNNNKNLLSHASKPTLTQQAKQFLPSSPTDGAIFSVGTPPRLSSNVDGDDNKWV
AQSTQPTSNSQENKNSPTREEKSIATCGSSSEINEALLHFYCKLSDAIDDVASEHALPLNTQLIAGGFLSR
STVQRQHETFSTDPLHNVTSEQRQVEQVLLYIRSLQILATALHTVRNKVKAGELQLNEEMRQLIGDLNKRY
KVSCKRCQDAKSKCDMTSLTQKSYKSADRLLYYYAVHDCRTSALDEMFEGSVERCMKRYRRALVLLEGISL
SASDALDKQRLAKYKASIDHRLQHLEKLWSTKVNS

>Ciona_robusta_Ulk3_XP_002128179
MASSSKSINKTTYVTPKLKDFVFTEKLGSGTYATVYKAYRKSCEHRQVIAVKCIQKSNLNRVSIENLLLEI
EILKQIKHEHVVELFDFQWDDSFIYLIMEYCGGGDLSGFIQSKRMIPEYTVRRFLQQIASAVKVLHDHNIS
HMDLKPQNILLTSNYQPVLKIADFGFAQHIESVQEYSLRGSPLYMAPEMILMKKYDAKVDLWSIGVILYES
LFGEAPFASRTLEDLEAKIQSQDPILVPRTPQTSNDCKNLLYGLLRRDPDQRISFEDFFAHPFVDLEHKPS
QDCLPKATEIVMSAVASDRAGDLAKAAKLYSEAIEYFVPAIYYENDQLKKAALRTRVTEYCNRAEQLKILS
KPVTTPTDSLEQLKLLSEDNQVITEAIQMIFEARKAEKNDKFQQALDLYTEVLGNLIPILGKEKPTRRKEL
LHSQVQFLLTRAESIRSYVSVQEIEIQQPNVQVENKPTKCSIS
```

>Ciona_robusta_Ulk4_XP_002125058.1
MENFVLYEEIGRETSSIVYKGRRRGTINFLAIQCIEKDRRAAITNWVRLTHEFNHQNIVQFHEWYETSNHL
WMVTELCTGGTLDECLRQDSCLPGVIVCQFGKDIIRGLNYLHEHSVVFADLCPKNVLLDGPGVLKLTNFCL
SRLVDEDLNEVYLQAANKMEDSDSMSIVNDNLLGELHYRSPEILKGGNPTTNSDIWAFGVLLYRMYSGTVP
FSGKDDNFISQVLQNDFLDPQQKRNKLHYQNTHVLSDGQISEDFQHLLSKTLEKDLNVRFNSKQALNHKLW
NVIEETDITMDESGADGFNVAEDSLRDTVSSLSFAPESVNVLDSTFTLSSRPRTANHRENEKVVKNSTAKS
VPINNSSIQVQSIDKTMTFSQTLDIERDSLSICEPEIQQESDITFEDIQNVIYCDTEPKECIIQDNTQIKK
TEQLRWDAKTIPFKLLSQVHNTTPDELYSHLKLIVDLFTQQSASNNSQSRLKANVLSYLSHLCVASPEVSN
FVVNSNLLLLMVNLCKNSMQQFPDMAFKAIRTIGLAARSATELQQCTQVTEVFNVLSEILREGFKHDRIKF
SIVPALGQLLCLVATHEDSLLVTTDGRATSSESHGQRKGNWYIPGATYTLITRCLHEGEDSALQHYACKII
ECVACVSDQHGRKFISISPQSSGGSSHSRDVIGPLLWSTSDKAKSEGIRSTSLMALCSLNRISGSGSIFQA
VLERAGVQSVLIFLNRSSYKCQQAILTSFNETLNSGQKGHSVRLANAKDILPSIMKLLDSPSNLVRAKVNL
SLLLLLRLNPKLLMTACQHRLIMHIERDCRKISPSHFARPLHVQHVSEEDKYIHSTLCLLIRYMILQSKEV
TTSSIDALNEAMERKHPSSKQVKQLRISLPCCMVLQNLITSHVFRMQMVTENFIENVGKLLDHAVKFVKKA
PASSAGIVTAIGDDTAQKFIDTILSIIEVLSQHPPDLANNEEASMKCLVPRLVILCTVSDSNERQAGCVKL
LTDVLDTLLHFRAISTDASTVTLTNERNSIKTPASRLSSRATVRHEMPDEFGIRECVIVNIIPEFQSILLR
QMPLPAYGVRLLSILLEHWPSISTSLCYYGVIPILFKLLDEHKSSPLSSSYQGIITIFIYLIQYRNVDVVN
ALYNNGLVETVNTCLLENFSFYKQVRKNPDDDGAVDCLSHNLSLLKYMLDRVTTAVKEALLAKEDSNSARP
NTTEGAERLLVLHKPLTDLHTVCISLLCEEKELSDSALACLSLLVQLYGGDHPDTMLTENMNSLAAALTVA
NQKNQRILLKLIRRIATLNVHHAHRLASEDGQLLIENVRNTLYAAEQCADSSLAHLANEILVSIKDKRKE

>Danio_rerio_Ulk1_XP_002665971.2
METVGKFEFSRKDLIGHGAFAVVFKGRHREKHEWEVAVKCINKKNLAKSQTLLGKEIKILKELKHENIVAL
HDFQETASSVYLVMEYCNGGDLADYLHSKGTLSEDTIRVFLQQITGAMRVLQAKGIIHRDLKPQNILLSHP
AGRKSHFNNTCIKIADFGFARYLQNNMMAATLCGSPMYMAPEVIMSQNYDAKADLWSIGTIVFQCLTGKAP
FQASSPQDLRLFYEKNKTLSPNIPRETSTHLRHLLLGLLQRNHKDRMDFDEFFRHPFLEASSSMKKSTPVT
VTCFPNSASASSCSSSSTSHLASPPQSLAEIQHVRAKALASPTQDSPGYLLKDSGGGGGGSSSKNSSCDTD
DFVMVPAHFPSELTCDMPTGKVLQDSLMYSGSSLLASGGQCSQGKTPPRSPSFSSSPGPSGRPSEFSGSSY
GNYGQSVPIPVPTQIHNYQRMEQNFQFPGQEGSPRSGLAQRCSSGSFLACGRTGPSPPRSGSAVMSRRLST
GGSKPFQLSPQVGTIPELPGQVCPASAEVGHRGSRGGEAKTWPQQQGLGTRLNSAPCLLEAAGGCRQKIRK
QHSDPVVAPQANVMPFRTLHSSPRLSELMQRNPLPTILGSPSRAMPPFEFPKPPSSPNMVTFLTQQGLRTA
QGEPSYSPAYQPEERKGFGRSQSAGRLSDVLLMAAFGGQRGERGSMENLNTDRAIDITAPPGGGGIVVGSG
SPARVVFTIGSPPSGATPPQNSRSRKLSAGSSSSVSPVGSLTSRYTQAGNYMDCYEGSSSPRYGFSDPISA
VTFEAPELPEETLMEQEHTETLCRLRFMLDFARCMVEVAGARSGEMAQTELSSTSLLQQQSLMADQISSLS
REWSYAEQLVLYMKTAELLSSSLQTAMEGIKQGKLYPSSTVKQVVRRLNDLYKSSVMSCRSLSNQLERFFT
RKHKLMDHINSITAERLLFGHTVQMVQAAALDEMFHQGEASIQRYHKALLLMEGLSLLLTEQADILSISKC
KQCIERRLTALQSGLCV

>Danio_rerio_Ulk2_XP_002664661.3
METVGDFEYSRKDLIGHGAFAVVFKGRHKKKTDWEVAIKSINKKNLSKSQILLGKEIKILKELQHENIVAL
YDVQETPSSVFLVMEYCNGGDLADYLQAKGTLREDTLRVFLQQIAAAMRILNSKGIIHRDLKPQNILLSYT
GRKKSSINGIRIKIADFGFARYLQSNMMAATLCGSPMYMAPEVIMSQNYDAKADLWSIGTVIYQCLVGKPP
FQANSPQDLRMFYEKNKSLVPNIPRETSPQLEDLLLGLLQRNQKDRIDFDTFFSHPFLEPISTIKKSCPVP
VPSCSGLVSDSTCGSSPSCRYVSPPSLPDMQTLPEDVLSSPPLGPPNYLQLSKESGGSTSSKNSSCDTDDF
VLVPHLSGEQSYDLPMGAVGRRPSSEFLLCGGSPQPSTGQTPMVSPRSETTPIPVPTQVRNYQRIKQNLSS
SPTTTLYGSPRSGTVRRSNTSPMGFPKMVSASPSPADTVQTVGRRLSTGSSRPYSPSPLVGTIPEQLGHCC
CGHPQSHEPRSRSSSGGSPVPSSQLLGARLQSAPTLTDIYQSKQKLHKQLSDPVHPTSSAYPSNHSPQLGR
PANLGTSPTKHLGSSPRTSDWLTKSPLPTIIGSPTKVTAPFKIPKTQASCNLMALADSPIPNKTLMDGREL
CAHHCTAYPSSRQPAPEASKTSFGRSVSAGRLSEPPVRITLGGQPYQGSTDSLNTERPMDTAPAGMCALAA
GGGSPRTVVFTVGSPPSSSTPPTCSHLASRPRATSVGSNSSAGSLCSTSGKVYMGSPPGMTIGSSPPGAEA
GPSSLRYVPYGTSPPSLDGFITFEAPELPEETLMEREHTDTLMYLRMMLSFTDCVLEIAALRAGGPDLGAS
AASLYPPQDSVVVDQISQLSREWGQVEQLVLYMKAAQLLASSLHLAKAQIKSAKLNPSTAVKQVVKSLNER
YKSCISLCRRLTDKLNHFFSDKQRFVDEINSVTAEKLIYNHAVEMVQSAALDEMFQQTEDIAYRYNKASML
LEGLTKILQDPADIENVIKYKASVDRRISALCYCTVTLYE

>Danio_rerio_Ulk3_NP_001082941
MAAAGFAPPKLKDFILTERLGSGTYATVYKAFRKTDSREAVAVKVVSKKSLNKSSMENLLTEIEILKTVRH
PHIVQLKDFQWDSENIYLILEWCSGGDLSRFIRSRRILPERVARRCLQQIACALQFLHERNISHLDLKPQN
ILLSGNMLKLSDFGFAQYMSPWDEQHALRGSPLYMAPEIVCRKHYDARVDLWSVGVILYEALFGRAPFASR
SFTELEEKIRSERPVELPAAAGVSRDCRDLLLRLLLRDPDRRICFEEFFLHPFVDLEHMPSAESLPKAKAL
VLQAVQKDQDGDRSAALSLYCSALEQFVPAIHYETDRQRKEALRQKVNQYVCRAEELKALVRSDNKISFEE
ARSARNILIEMSRDQPRLLAALEVASTAVAREESGAEDYDTLDLYQQSLGEMLLALAAEAQGRRRELLHSE
IKSLMSRAEYLKELIKMRETQTDESLKKDAAAESVRSSCCLQ

>Danio_rerio_Ulk4_XP_009290329
MENFILYEEIGRGSRSVVYKGRRKGSIHFVAIICSEKHKRPELTNHVRLAHDIKHDNVVAFYEWYETSNHL
WMVVEMCTGGSLAALIAQDECLSEYVVREFSIDLVKGLKYIHDSGIIVSDLTPAKILLDGPGTLKYSNFCL
AKAQGESLEEFFSLVMAEETGLGESKENNSSPRNIKNRIKGSPVYCSPEVLRGSETNVDSDLWALGCVLYE
MFTGKPPFVSESMTGLVDLILNEDPPPLRPKEPASSQPSPDFENLVMGLLQKDPVLRLSWDPLLSHPFWRD
AFLEAHNSKGEPQDDVSSISSLHLAPVAPQTAEEAQTAPADPASPNSKSFTLDNVIEFRPKSALDIDAREA
IFLLSSRPTPRTSTARETEDSRAEIHESSVMAEEQSDLNTCIKSLLYTDCDLTVTPIMDNPKILKTAPVRF
DPKTLCVPAHSAERLSCLSSEDWSQFLQQVCAVLESVDRAGSANATAPRAKLNLLCYLCTVCNHRDTATRL
IHSELFPVLVQQMRQAPNWDIRSKVMRAIGLLASHCTELRDHAPVSEAVAMFTELIRENFRSSKLKQCLIP
PMGELLYLIATQEEKKERPGGLWVVPAAAYTVLMRCLREGEDLVVNHMAAKTIENVCSRESQSAQGFITAE
VGPALWYLFTHSTVDALRVSAISALCKITRQSAGAFQSVIDKVGLPSILSCLVSGISRVQQHMLTMFAAML
ASGAHSHRLVQDRDFVMKIIRSLESPSSVIRAKAFLVLLQVLMNNREMLLLCCNSRLVMYIERDVRKATPG
KEQQSGNEYLSKCLTLFIRHTVRELPAILDDILSVLGSIVGRKHPSPTQSRQLKQSLPMMAVVLHLLTSQI
FRPQVVTEAFLIKFGKLLNHITSIDSNETSLGSAIGQASSEELIRNTLSAVEAISQHPALLSLYPFIVVDL
IVPPLASLAFSKNVEWRIVSLRVLSEITLLLLSQDEVEERERAGEGEREWEGEGNISSNTRLLTLISEALL
PQYETLLLEPDPVPVYALKLLVSLTEHSSPISRLVRESRLLPAIFQVIEEHQSNSVGGTMQNAVALLCNLI
GQKGTDLRPFYQQGVIEVVCNVFVEVSGVYLEREMQSGLKSCSSLLVSLLDIIHILLKNLSSVVRMALQRS
DSNEDAEAAEELLLINKPLTDLNNLLIQLLTCDDSEVYEEASHCVSLLAQLYGGEGADRLQPEELLSLAHA
LQTHTEPRQQKLLLRVLKRLMSAVGSSCWGSSEGQILVHTLQKLMLLNRPQSDMTVESLAADILKSIGSHT
ETLSPGI

>Homo_sapiens_AFK_NP_000692.2
MGKGVGRDKYEPAAVSEQGDKKGKKGKKDRDMDELKKEVSMDDHKLSLDELHRKYGTDLSRGLTSARAAEI
LARDGPNALTPPPTTPEWIKFCRQLFGGFSMLLWIGAILCFLAYSIQAATEEEPQNDNLYLGVVLSAVVII
TGCFSYYQEAKSSKIMESFKNMVPQQALVIRNGEKMSINAEEVVVGDLVEVKGGDRIPADLRIISANGCKV
DNSSLTGESEPQTRSPDFTNENPLETRNIAFFSTNCVEGTARGIVVYTGDRTVMGRIATLASGLEGGQTPI
AAEIEHFIHIITGVAVFLGVSFFILSLILEYTWLEAVIFLIGIIVANVPEGLLATVTVCLTLTAKRMARKN
CLVKNLEAVETLGSTSTICSDKTGTLTQNRMTVAHMWFDNQIHEADTTENQSGVSFDKTSATWLALSRIAG
LCNRAVFQANQENLPILKRAVAGDASESALLKCIELCCGSVKEMRERYAKIVEIPFNSTNKYQLSIHKNPN
TSEPQHLLVMKGAPERILDRCSSILLHGKEQPLDEELKDAFQNAYLELGGLGERVLGFCHLFLPDEQFPEG
FQFDTDDVNFPIDNLCFVGLISMIDPPRAAVPDAVGKCRSAGIKVIMVTGDHPITAKAIAKGVGIISEGNE
TVEDIAARLNIPVSQVNPRDAKACVVHGSDLKDMTSEQLDDILKYHTEIVFARTSPQQKLIIVEGCQRQGA
IVAVTGDGVNDSPALKKADIGVAMGIAGSDVSKQAADMILLDDNFASIVTGVEEGRLIFDNLKKSIAYTLT
SNIPEITPFLIFIIANIPLPLGTVTILCIDLGTDMVPAISLAYEQAESDIMKRQPRNPKTDKLVNERLISM
AYGQIGMIQALGGFFTYFVILAENGFLPIHLLGLRVDWDDRWINDVEDSYGQQWTYEQRKIVEFTCHTAFF
VSIVVVQWADLVICKTRRNSVFQQGMKNKILIFGLFEETALAAFLSYCPGMGVALRMYPLKPTWWFCAFPY
SLLIFVYDEVRKLIIRRRPGGWVEKETYY

Tnfr16

>Paracentrotus_lividus_Tnfr16
MFCPPGFGAVVPCSPQQSSRCELCDNGTYSDLVSSTEGCKTCSVCKEGSIVLKRCTDISDTVCSDTYLPPV
TSQPANDDITTMVWIPRSGPTSSGFSVVPIFCTLLGLVIFGLLAYVIFKKWSFKKMKLRQTQKNMTRSSSC
HTDIEGNTISILSLKNGQSYYASRDSALDRSGAGSICRQPLMAVPSAMVYQQLPPDKRYEVERALSASRMD

GRDWRGLARELGFSDLDIVHIAQTCTGSTPPGRAMLITWHSRDPKRASVGTLVEALRRIRRNDVADLIPVF
TYSAYHFPTS

>Strongylocentrotus_purpuratus_Tnfr16
MHFKLAHLCGKIIESCKVSTRMDHFIRNRILVTLMCWAFWITSVSANNVTNTMPAETQEPSTSFTPTGIPM
NSTVTETAECKTGVYSTTGECCEECPDGFGVHRQCSDPNATNTICTICETGMTYSSVTSHLMPCQTCTRCS
HNEVMTSSCSIMQDTECDCAPNYFRTDPQSGVPATGTASMCSQCMFCPPGFGAAVPCSPRQSSRCELCENG
TYSDIVSSTEGCKKCTVCREGSIVLKRCTDISDTVCSDTYIPAVTDRPENDGITTTAWIPRSNRTSSGFSV
VPIFCTLLGLVIFGLLAYVIFKKWSFKKMKLRQTQKMTRSSSCHTDIEGNTISILSLKNGQSYASRDSALD
RSGVGSICRQPLMAVPSAMPYQQLPSDKRYEVERSLSVARMDGRDWRGLARELGFSDLDIVHIAQTCTGST
PPARAMLISLHSRDIKRATVGTLVEALRRIRRNDVADLIPIFTYSAYHFPGS

>Homo_sapiens_TNFRSF16_NP_002498
MGAGATGRAMDGPRLLLLLLLGVSLGGAKEACPTGLYTHSGECCKACNLGEGVAQPCGANQTVCEPCLDSV
TFSDVVSATEPCKPCTECVGLQSMSAPCVEADDAVCRCAYGYYQDETTGRCEACRVCEAGSGLVFSCQDKQ
NTVCEECPDGTYSDEANHVDPCLPCTVCEDTERQLRECTRWADAECEEIPGRWITRSTPPEGSDSTAPSTQ
EPEAPPEQDLIASTVAGVVTTVMGSSQPVVTRGTTDNLIPVYCSILAAVVVGLVAYIAFKRWNSCKQNKQG
ANSRPVNQTPPPEGEKLHSDSGISVDSQSLHDQQPHTQTASGQALKGDGGLYSSLPPAKREEVEKLLNGSA
GDTWRHLAGELGYQPEHIDSFTHEACPVRALLASWATQDSATLDALLAALRRIQRADLVESLCSESTATSP
V
>Danio_rerio_Tnfr16_XP_003198133.2
MDTSLWIVICAALGLVSSSKLELAVEEECESGSFTHSGECCVQCPPGEGVIKECGATQTECTQCLDSETFS
ATFSHTDKCQVCTECTGLMRMQTPCTDSNDAECVCNYGYFMNVLSSRCEPCTVCPLGQGVDMRCELNHDTV
CEECRDETYSDQENTMDPCMPCTICEEDTEILLRNCTPTEDALCHDPLSPTYPTSTGDSGSFDTDLDRLWS
PSPGDDATTPKPSSPHFIGRGLNENLIPIYCSILAAVVVGLLAYIIFKRWNSCKQNKQAANNRAATANQTP
SPEGEKLHSDSGISVDSQSLQEQQAQTQTQAQAQAHTQLHAAEQIVVRVDGATQPDSPPAQA

>Acanthaster_planci_Tnfr16_XP_022104865
MFLRKKAVLICFAVVNWLTYRYLAECTPLPSPECPSKETTSTGDCCLQCPPGWGVQSRCSAFNNTICMHCY
ADITFSPEPSHTAGCMPCKRCARNEFVRHSCNITHDTLCECRRNYYHTADGSCSQCNTCPPGFGATVKCAT
QHNTECESCANGTYSDLTSATKGCVPCSTCLEGTVVLEMCTRFSDTVCSDTVMSTTTAYQHSSETPDTVAH
VINSRPPSIVPIYCAILAFVVIGLLGYVIFKRWSFRKVKLRSHAKQTRSPLGSPSKADIDVSSAGAHSIKK
SADLPPSLAPSRQPTDQTPLMTVPSTTLYRDLSTTTRSEVESLLGISRLDRRDWRGLAHELGFSDKDIVHF
IKTSDDDLPVHRMLTVWSDREGAVVSVLVAALKSLNRQDVLQKLPVFA

>Apostichopus_japonicus_Tnfr16_PIK52601
MGLFRKNGGVCSQCSVCPPGFGTIQPCTFLEDIICEECVPLKTYSDVVTRATVCKPCTRCRRGTFIVENCT
KYSDTVCSPPPSSGVSLTPPSDPTGHPYHPGVSIVPLYCVLLAALVVGLLIYVLFKRWSFHQIKLRASQKM
VRSNTSSTDIEKNPENLNGSQVLTATTYTGPSSYVVATVSPSTDVSPTTLYKELHSDKKVELEEKLMISRK
DGRDWRALCTQLNFTNMDKEDFSKSKDGHVNPVHQMFHKWQSRDENHGNSERS

>Platynereis_dumerilii_Tnfr16_AMZ00104
MDYLTVAVSSVLLLLAHGPVSILTEVEMRPCEPGISDHLTSGCCSVCPAGTGVVEACESGRDTLCSPCDDG
STYSSSQSHEERCKNCRVCDENAKLISPCTITNDTVCECRQGYYFEKYEKICKRCDSCPEGFGMVKKCTPH
HNTKCKRCPEGLFSDRRSHKGCRYCSVCRPDQLTLHVCNYREDTVCMEMPVPVTSSDLQSSNLDSNNEGSK
TSHGDIIPVYCSLLGALVLGLIIYVIIAHRRRQAANKLNRTGADGASPTSKQGSDSGVYVEPDQKQALLAK
RFLDLAPSTIRVVEGMLMTAGNNASDRRHLSTKLGYNTSQTANIEIRSRQEGCSPSHCLLADWGATDGATC
GELVKALRTIARDDIARILQPGNSDARRNGDAIEPLVV

Tnfr19/27

>Paracentrotus_lividus_Tnfr19/27
MAEPKVSYDGLEDCSSASFLNTGLQRMVVAIVILSILFSVQDSVDALPTSNPSSNHKDVNLTRQSLMMIDK
SMPDDDIQDLSSSSDYDSLPLTVQVAEDCSSDQYLHQSGVCRNCTTCGPGTQHGIDKKCGYGNGFEQICEP
CPDGYYQGGTRSDLVRCNRCITCDELAETVQECSSVNNTECGPCPAGKYYYYPNVCFPCEAGGDDKEECMA
TKPTTPAPTSSTSPPPIRTTVNMTFFNQTESPGNTTEEPSPGGTNKIVTACVTVFVIAIPVSLVAICVYKF
LKKRHSRSDEESTGEKGGEENGSKSGDPGVISDQAQYNARTGAVDLVRYSPVPLQEPPENGTDLSLPQTLR
SDLPFDPPPCVCNGQSQVEPDQGLHALAPAVSIVGQDTPPSRR

>Homo_sapiens_TNFRSF19_NP_061117.2
MALKVLLEQEKTFFTLLVLLGYLSCKVTCESGDCRQQEFRDRSGNCVPCNQCGPGMELSKECGFGYGEDAQ
CVTCRLHRFKEDWGFQKCKPCLDCAVVNRFQKANCSATSDAICGDCLPGFYRKTKLVGFQDMECVPCGDPP
PPYEPHCASKVNLVKIASTASSPRDTALAAVICSALATVLLALLILCVIYCKRQFMEKKPSWSLRSQDIQY
NGSELSCFDRPQLHEYAHRACCQCRRDSVQTCGPVRLLPSMCCEEACSPNPATLGCGVHSAASLQARNAGP
AGEMVPTFFGSLTQSICGEFSDAWPLMQNPMGGDNISFCDSYPELTGEDIHSLNPELESSTSLDSNSSQDL
VGGAVPVQSHSENFTAATDLSRYNNTLVESASTQDALTMRSQLDQESGAVIHPATQTSLQVRQRLGSL

>Homo_sapiens_TNFRSF27_XP_011529302
MDCQENEYWDQWGRCVTCQRCGPGQELSKDCGYGEGGDAYCTACPPRRYKSSWGHHRCQSCITCAVINRVQ
KVNCTATSNAVCGDCLPRFYRKTRIGGLQDQECIPCTKQTPTSEVQCAFQLSLVEADTPTVPPQEATLVAL
VSSLLVVFTLAFLGLFFLYCKQFFNRHCQREKLIIFSDPVPASLNLIPEFAGGLLQFEADKTAKEESLFPV
PPSKETSAESQVSENIFQTQPLNPILEDDCSSTSGFPTQESFTMASCTSESHSHWVHSPIECTELDLQKFS
SSASYTGAETLGGNTVESTGDRLELNVPFEVPSP

>Strongylocentrotus_purpuratus_Tnfr19_XP_011663931
MLKYIRISVTFIVTAASLVFMVPAACSSPVPPRNISQVEQTAETKDPGDSCLWNQFVNPNLTCENCTFCPQ
GTEHSPNEQCGYGRGAKTTCTPCAEGFFQTGVGYTGLVCRRCVTCDLNVEYLQNCTTTQDASCGPCPPGEY
YLSPNICFECKHDPSHADCPQPPIIKPSTASPNQSAPTLPSSVPLPESTAPVIYTTPPPLSSQAHNTSNNF
DEFIDLQ

>Danio_rerio_Tnfr19_XP_017206452
MPQAQILSRFPLQRILILPLCVLFAVMAEERRDCREQEYKDKFGSCIPCRQCDAGQELSKECGFGYGEDAQ
CVPCRASRFKEDRSLQKCKPCLDCSLLNRFQKGNCSTTNNAVCGDCLPGFYRKTKLRGFQDMECIPCGDPP
PPYETHCMGRVNLVPLPSTVSSPRDMALAAVICSALATVLLALFILCVIYCKRQLLEKKPVSMRAHEGPFL
GSELSCLDRRVLELSQRPCCHCTHGSEQTCGGVQLVSSVCCEDVWSQNRRRDAPAFHSHCRISDNGLTNES
VGSQTDMACAPDEVWPLVQSRRTTDSLQTNQRCSSCEEDEEEVQISDQRTAEEPEDASHKPLIQQTTDTEG

>Danio_rerio_Tnfr27_XP_017211491
MDCLTDEYYHNGECHKCPQCPPGQELKEDCGYGEGVPAVCGVCDVRWFKEDWGSHPCGLCQNCRRLNRHQI
KRCTHTDNAVCGNCLPGFYSKMRLDGLEDLECLPCGPAPFRNIQCSRGEVTGEAKVQTSAAPFTNASSIVT
ACAATAILTTFLFAIVCVTYQTRASLRKKCTSCLSSVSDGHYDSNAASVPMSTLHTVTQDAEANRSNPRLP
LDDITTLLTSDLRLHGCEVLPLAAESASADPATGLIRQATETPDVSSSDSALSSQHVILTLLGEQSTVRPC
CAVEQRAAWGLHAPVECTELDLQHLSSSPDLQTYKPEISHNVWAPTPVHPQA

>Branchiostoma_belcheri_Tnfr19/27_XP_019618758
MWPDVVATNHLLQRNVAGRQREHTDIMPDVSKLLSVMSQWNITDKSSYRTFRGLLCFLVFTCAASVDDCPE
NEYYMEKWCIPCTICEHLNKEELQPCLPQSDAVCGDCFPGYYMDQRYCHSCEYAPADNRYCQLWLASQTTS
RPAAGTTISTSTHAQIQHSVVQTTPPSVTEEAPGKPTSRSLYPGEIAAIVVSSLVGLALFIGVLVLVKIYR
NNNEEATPIQATGEDQQGHNYT

>Mizuhopecten_yessoensis_Tnfr27_OWF43094
MDKTPISREHKVSFCKQNYTNDYCHRCPVGSVQSKAVDSATVNHLTEILKCVSTDQEPSCPPETVLVLSER
KRPTCECDTARGYAGDNFLICISCPECPPGTELKKDGQCSHCPPGTFKTSQGFGPCRRLTNCGGENRETLL
QGNTTADAVCGNFLTTATTESASSVPDMSTRESQPILAALSSDGLEGFSLTGDENNPIYSSHEMEMHDIYN

NQQRLMLVTMAIQVIFFIAILVIITVGVLKIRKMSRDLPTRRGKDVGKDEECLLVASPSDNDTETARIKSA
YALDKYESEQEFVSLYQPVTMETLYPDINSRPCTPTAPRPSILDFRNNC
