# Supplementary material for: Identification of Cell Death Genes in Sea Urchin Paracentrotus lividus and Their Expression Patterns during Embryonic Development
Source: Genome Biol Evol. 2019 Jan 29;11(2):586–96. doi: 10.1093/gbe/evz020 (PMC6394757; doi:10.1093/gbe/evz020)
Supplement: Supplementary Data [file evz020_supp.zip › Supplementary information III_27.02.19.pdf]

Supplementary information III

Primers used for the gene expression analysis during *Paracentrotus lividus* development and the relative amplicons validated by double-strand sequencing.

Aifm1

Forward primer 5'- TAGTGGCAGTGGGTCTGGAA

Reverse primer 5'- CGCCCTAGCTTGATGTCGTA

Amplicon (163 bp):

TAGTGGCAGTGGGTCTGGAACCCAACACTGACCTTGCAGCAAGTTCAGGGTTAGAGGT
CGATGATAAGTTCGGAGGTTACAGAGTCAATGCTGAGCTTGAAGCTCGTAGAGACGTT
TGGGTGGCTGGTGATGCATCATGTTTCTACGACATCAAGCTAGGGCG

Bax

Forward primer 5'- CGTATCGAGCAGACACGGTT

Reverse primer 5'- GCTGGAAACGCTCCACAATG

Amplicon (100 bp):

CGTATCGAGCAGACACGGTTTGAGCGTCAGGTGAGCCGCGATGATGTTGGGGAGCAGG
CCACTGTTCTCCTTCAACATTTCATTGTGGAGCGTTTCCAGC

Bcl2

Forward primer 5'- TAGGGGTATAGCGGCAGTCA

Reverse primer 5'- GGCATCCCATCCTCCTTGTT

Amplicon (91 bp):

TAGGGGTATAGCGGCAGTCACTGATTTTGCAGCACAGTACATTGAGGAAAACCTAGCC
CAATCTATTATAGAACAAGGAGGATGGGATGCC

Parp

Forward primer 5’- CCAAGAACCCAATCAAACGCC

Reverse primer 5’- CCTGCACGTTCTTTACTAG

Amplicon (97 bp):

CCAAGAACCCAATCAAACGCCCCATGAAGTTCTTTCCTCTGGATATCAGCTATGGAGA
GGTATCTAGTAAAGAACGTGCAGG

Pink

Forward primer 5’- GCAGTTGGTTACCTTGGC

Reverse primer 5’- CGCAATGAAATCGCACATCC

Amplicon (137 bp):

GCAGTTGGTTACCTTGGCAACCAGGGTGTGGTCCATCGTGACCTCAAGAGTAACAACA
TCCTCGTTGACTACGAGGAAGGCTCTGATGAGGTACCCCACGTGGTGGTCGCAGACTTT
GGATGTGCGATTTCATTGCG

Tnfr16

Forward primer 5'- TGGAACCTACTCGGATCTCGT

Reverse primer 5'- CATTGGCTGGTTGGGAAGTC

Amplicon (155 bp):

TGGAACCTACTCGGATCTCGTCAGTTCAACGGAAGGATGTAAAACATGTTCAGTCTGC
AAGGAGGGTAGCATTGTACTCAAGAGATGCACTGATATCTCAGATACAGTATGCTCAG
ATACCTACCTACCACCAGTGACTTCCCAACCAGCCAATG

Tnfr19/27

Pl_Tnfr19/27

Forward 5'- CAACTGAAGAGCCTTCTCC

Reverse 5'- GCGTTGTACTGAGCTTGATC

Amplicon (226 bp)

CAACTGAAGAGCCTTCTCCAGGTGGGACAAATAAGATTGTTACTGCATGTGTGACTGT
GTTTGTCATCGCTATTCCAGTTTCCCTTGTTGCAATTTGTGTATACAAGTTCCTCAAGAA
GCGTCATAGTAGAAGTGATGAGGAATCTACTGGAGAAAAGGGCGGTGAAGAGAATGG
ATCAAAGAGTGGAGACCCTGGTGTGATATCTGATCAAGCTCAGTACAACG

Ripk

Forward primer 5'- GGAGGCTCTTTTGGAGACG

Reverse primer 5'- CGATGAACTCAGACGTGAGG

Amplicon (151 bp):

GGAGGCTCTTTTGGAGACGTCTTCAGAGTCAATCATGCCATTCATGGGGTTGTAGCTGT
GAAGCGTGCTAAACCTGGCAAAGGAGATCATATCAAGATGTACAAGAAGGAGATTGA
GAAGCATGTAAAATCCCTCACGTCTGAGTTCATCG

Ulk1/2

Forward primer 5’- TTGAAGGCTAGGACACTGGA

Reverse primer 5’- ACTGGCATTGGGGAAGTTGAG

Amplicon (180 bp):

TTGAAGGCTAGGACACTGGACCCTAATATACCACCTGGTACATCAAGGGCCCTCAAAG
ACTTGCTCATAAGACTTCTCAAAAGAAACCAGAAAGACAGGATAGAATTTGATGAATT
TTTCAATCATGACTTTCTATCAAAGACCCTGAAATCTACCTCAACTCAACTTCCCCAAT
GCCAG

Ulk3

Forward primer 5’- GTAATGGAAGCTGTGAAGGC

Reverse primer 5’- CTCTCCTCATGTACTCTAGGC

Amplicon (160 bp):

GTAATGGAAGCTGTGAAGGCAGACCAGAAAGGAGAATGGAAAGAGGCTATCAGGCTG
TACCTCAAAGCAATGGAATATTTTATTCCTGCAATCCAGTATGAGCGGGATGCAAGCA
AGAAGGACGCTCTAAGAATGAGAAGCCTAGAGTACATGAGGAGAG
