# Supplementary material for: Identification of Cell Death Genes in Sea Urchin Paracentrotus lividus and Their Expression Patterns during Embryonic Development
Source: Genome Biol Evol. 2019 Jan 29;11(2):586–96. doi: 10.1093/gbe/evz020 (PMC6394757; doi:10.1093/gbe/evz020)
Supplement: Supplementary Data [file evz020_supp.zip › Supplementary information V.pdf]

NCBI **BLAST Search Results** BLAST Entrez ?

TBLASTN 2.2.26 [Sep-21-2011]

Reference:
Altschul, Stephen F., Thomas L. Madden, Alejandro A. Schäffer,
Jinghui Zhang, Zheng Zhang, Webb Miller, and David J. Lipman (1997),
"Gapped BLAST and PSI-BLAST: a new generation of protein database search
programs", Nucleic Acids Res. 25:3389-3402.

```
dbase=Pliv_genome_110914
           77,240 sequences; 1,213,013,516 total letters
```

Query= Hs_BID_NP_932070
(241 letters)

Distribution of 1 Blast Hits on the Query Sequence

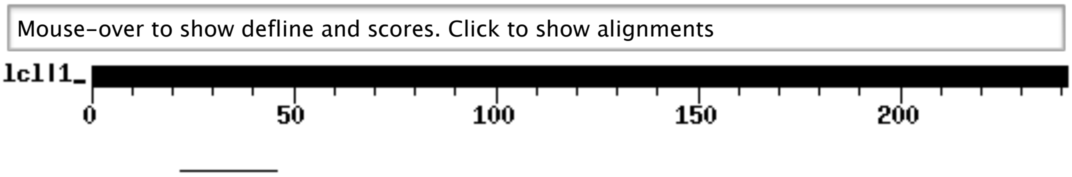

```
                                                                 Score    E
Sequences producing significant alignments:                      (bits) Value

scaffold09500  length=32846                                        32    9.6


>scaffold09500 length=32846
          Length = 32846

 Score = 32.3 bits (72), Expect = 9.6
 Identities = 13/25 (52%), Positives = 19/25 (76%)
 Frame = -2

Query: 23    TVRILSPLGHCEPGVSRSCRAAQAM 47
             TVR+ +PLGHC   VS+S R+ Q++
Sbjct: 31657 TVRVYNPLGHCSSYVSQSTRSLQSL 31583



  Database: Pliv_genome_110914
    Posted date:  Dec 5, 2014 10:59 AM
  Number of letters in database: 1,213,013,516
  Number of sequences in database:  77,240

Lambda     K      H
   0.317    0.132    0.383

Gapped
```

```
Lambda     K      H
   0.267   0.0410    0.140


Matrix: BLOSUM62
Gap Penalties: Existence: 11, Extension: 1
Number of Hits to DB: 255,417,588
Number of Sequences: 77240
Number of extensions: 3151132
Number of successful extensions: 12139
Number of sequences better than 10.0: 2
Number of HSP's better than 10.0 without gapping: 2250
Number of HSP's successfully gapped in prelim test: 389
Number of HSP's that attempted gapping in prelim test: 9470
Number of HSP's gapped (non-prelim): 3838
length of query: 241
length of database: 404,337,838
effective HSP length: 122
effective length of query: 119
effective length of database: 394,914,558
effective search space: 46994832402
effective search space used: 46994832402
frameshift window, decay const: 40,  0.1
T: 13
A: 40
X1: 16 ( 7.3 bits)
X2: 38 (14.6 bits)
X3: 64 (24.7 bits)
S1: 41 (21.7 bits)
S2: 72 (32.3 bits)
```

NCBI **BLAST Search Results** BLAST Entrez ?

TBLASTN 2.2.26 [Sep-21-2011]

Reference:
Altschul, Stephen F., Thomas L. Madden, Alejandro A. Schäffer,
Jinghui Zhang, Zheng Zhang, Webb Miller, and David J. Lipman (1997),
"Gapped BLAST and PSI-BLAST: a new generation of protein database search
programs", Nucleic Acids Res. 25:3389-3402.

dbase=Pliv_genome_110914
77,240 sequences; 1,213,013,516 total letters

Query= Hs_FAS_AKB11528
(335 letters)

Distribution of 26 Blast Hits on the Query Sequence

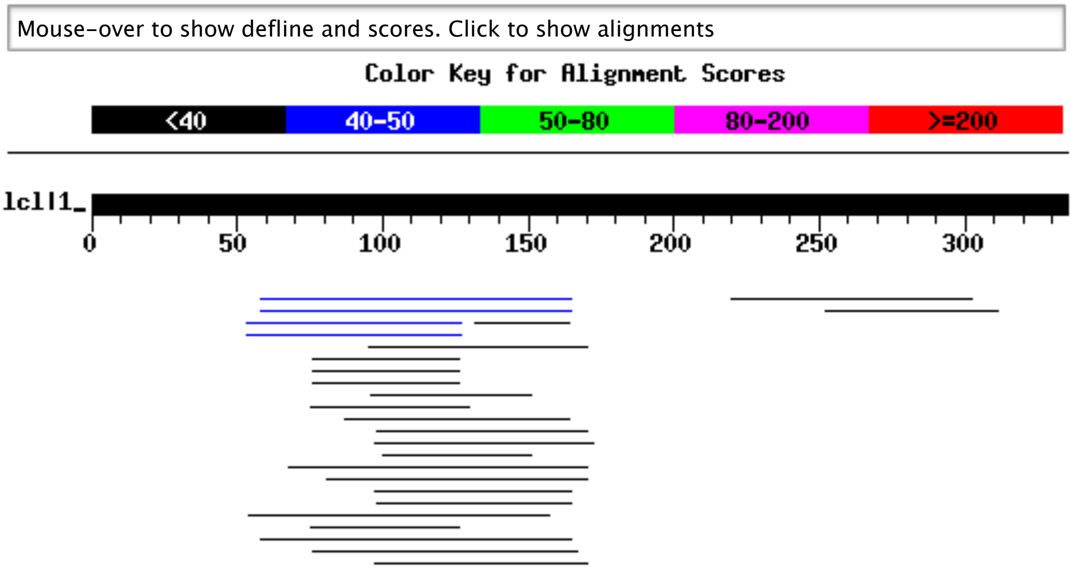

Sequences producing significant alignments:		Score (bits)	E Value
scaffold20496	length=5837	49	2e-04
scaffold08659	length=41819	49	2e-04
scaffold03892	length=85710	41	0.036
scaffold02052	length=107662	41	0.036
scaffold00322	length=218185	39	0.23
scaffold07317	length=49954	37	0.89
scaffold04074	length=83132	37	0.89
scaffold10507	length=29242	36	1.2
scaffold10268	length=24415	36	1.2
scaffold58435	length=2429	36	1.5
scaffold15527	length=11692	36	1.5
scaffold12257	length=24509	36	1.5
scaffold00596	length=176950	36	1.5
scaffold02627	length=124852	35	2.0
scaffold02238	length=99018	35	2.0
scaffold04742	length=51821	35	2.6

```
scaffold03852  length=78629                                        35    3.4
scaffold02123  length=107523                                       35    3.4
Scaff31                                                            35    3.4
scaffold54882  length=2536                                         34    4.4
scaffold45295  length=2872                                         34    5.8
scaffold13431  length=14941                                        33    7.6
scaffold14216  length=23502                                        33    9.9
scaffold07782  length=48146                                        33    9.9
scaffold04496  length=88722                                        33    9.9
scaffold01666  length=137803                                       33    9.9


>scaffold20496 length=5837
          Length = 5837

 Score = 48.9 bits (115), Expect = 2e-04
 Identities = 30/120 (25%), Positives = 46/120 (38%), Gaps = 12/120 (10%)
 Frame = -3

Query: 59   CHKPCPPGERKARDCT-VNGDEPDCVPCQEGKEYTDKAHFSSKCRRCRLCDEGHGLEVEI 117
            C + CP G    R C+   NG     C   C+ G  Y+       + C+ C   C    H    +
Sbjct: 4932 CCEECPVGFGVHRKCSDPNGTNTICTICEPGMTYSSITSHVASCQTCSRCS--HTEVMTS 4759

Query: 118  NCTRTQNTKCRCKPNFFCNST---------VCEHCDPCTKCE--HGIIKECTLTSNTKCK 166
             C+   Q+T C C PN+F                        C   C   C     G +   C+    +++C+
Sbjct: 4758 ACSIMQDTVCECAPNYFRTDAQPSGTSAVGTASTCSQCMFCPPGFGAVVPCSPQQSSRCE 4579



>scaffold08659 length=41819
          Length = 41819

 Score = 48.9 bits (115), Expect = 2e-04
 Identities = 30/120 (25%), Positives = 46/120 (38%), Gaps = 12/120 (10%)
 Frame = -2

Query: 59    CHKPCPPGERKARDCT-VNGDEPDCVPCQEGKEYTDKAHFSSKCRRCRLCDEGHGLEVEI 117
             C + CP G    R C+   NG     C   C+ G  Y+       + C+ C   C    H    +
Sbjct: 30601 CCEECPVGFGVHRKCSDPNGTNTICTICEPGMTYSSITSHVASCQTCSRCS--HTEVMTS 30428

Query: 118   NCTRTQNTKCRCKPNFFCNST---------VCEHCDPCTKCE--HGIIKECTLTSNTKCK 166
              C+   Q+T C C PN+F                        C   C   C     G +   C+    +++C+
Sbjct: 30427 ACSIMQDTVCECAPNYFRTDAQPSGASAVGTASTCSQCMFCPPGFGAVVPCSPQQSSRCE 30248



>scaffold03892 length=85710
          Length = 85710

 Score = 41.2 bits (95), Expect = 0.036
 Identities = 25/78 (32%), Positives = 32/78 (41%), Gaps = 3/78 (3%)
 Frame = +1

Query: 54   HDGQFCHKPCPPGERKARDCTVNGDEPDCVPC-QEGKEYTDKAHFSSKCRRCRLCDEGHG 112
            H G F H    P      A    +             + C Q            HFSS+C+ CR CD+
Sbjct: 6352 HGGVF*HAYEPASMSCAHISSGTPSRMKLIDCGQRNNALLSSPHFSSRCKSCRRCDQSTR 6531

Query: 113  LEVEINC--TRTQNTKCR 128
            L V + C   RT  T+CR
Sbjct: 6532 LAVRLGCRPPRT*RTRCR 6585
```

```
>scaffold02052 length=107662
          Length = 107662

 Score = 41.2 bits (95), Expect = 0.036
 Identities = 25/78 (32%), Positives = 32/78 (41%), Gaps = 3/78 (3%)
 Frame = +1

Query: 54    HDGQFCHKPCPPGERKARDCTVNGDEPDCVPC-QEGKEYTDKAHFSSKCRRCRLCDEGHG 112
             H G F H   P     A   +        + C Q         HFSS+C+ CR CD+
Sbjct: 33571 HGGVF*HAYEPASMSCAHISSGTPSRMKLIDCGQRNNALLSSPHFSSRCKSCRRCDQSTR 33750

Query: 113   LEVEINC--TRTQNTKCR 128
             L V + C   RT  T+CR
Sbjct: 33751 LAVRLGCRPPRT*RTRCR 33804




>scaffold00322 length=218185
          Length = 218185

 Score = 38.5 bits (88), Expect = 0.23
 Identities = 22/81 (27%), Positives = 35/81 (43%), Gaps = 5/81 (6%)
 Frame = -1

Query: 96     HFSSKCR-----RCRLCDEGHGLEVEINCTRTQNTKCRCKPNFFCNSTVCEHCDPCTKCE 150
              H SS+CR     RCR     H       C  + +++CR       +S+ C H    ++C
Sbjct: 170503 HSSSRCRYSSSSRCR-----HSSSSSSRCRHSSSSRCRHSSRCRHSSSRCRHSS--SRCR 170345

Query: 151    HGIIKECTLTSNTKCKEEGSR 171
              H     C  +S+ +C+   SR
Sbjct: 170344 HSSSSRCRHSSSIRCRHSSSR 170282




>scaffold07317 length=49954
          Length = 49954

 Score = 36.6 bits (83), Expect = 0.89
 Identities = 18/51 (35%), Positives = 25/51 (49%)
 Frame = -2

Query: 77    GDEPDCVPCQEGKEYTDKAHFSSKCRRCRLCDEGHGLEVEINCTRTQNTKC 127
             G E  C+PC++G   + +      CR C  CD      V+  CTR +NT C
Sbjct: 38544 GQEAHCIPCEDGLYQSQEGIAVLTCRLCLECDP--QAVVKSVCTRRKNTVC 38398




>scaffold04074 length=83132
          Length = 83132

 Score = 36.6 bits (83), Expect = 0.89
 Identities = 19/52 (36%), Positives = 25/52 (48%), Gaps = 1/52 (1%)
 Frame = +3

Query: 77    GDEPDCVPCQEGKEYTDKAHFSS-KCRRCRLCDEGHGLEVEINCTRTQNTKC 127
             G    C PC+E K+Y     +S  KC  C  CD      ++  NC+ TQN  C
Sbjct: 65601 GVNTTCDPCEEKKKYQPHEGYSQLKCTSCVTCD--LNADILQNCSATQNADC 65750
```

```
>scaffold10507 length=29242
          Length = 29242

 Score = 36.2 bits (82), Expect = 1.2
 Identities = 18/60 (30%), Positives = 26/60 (43%), Gaps = 4/60 (6%)
 Frame = -3

Query: 97    FSSKCRRCRLCDEGHGLEVEINCTRTQNTKCRCKPNFF----CNSTVCEHCDPCTKCEHG 152
             F   C+ C  C EG  +    NC    N +   +  +     C+ T+CE  D C+KC  G
Sbjct: 11222 FFQTCKWCYNCPEGACIPTAANCMEEHNCQVETQKEYHDRSDCSETICEASD-CSKCTTG 11046



>scaffold10268 length=24415
          Length = 24415

 Score = 36.2 bits (82), Expect = 1.2
 Identities = 17/51 (33%), Positives = 23/51 (45%)
 Frame = +1

Query: 77   GDEPDCVPCQEGKEYTDKAHFSSKCRRCRLCDEGHGLEVEINCTRTQNTKC 127
            G    C PC+E K    + +   KC  C  CD     ++  NC  TQN +C
Sbjct: 7177 GVNTTCDPCEEKKYQPHEGYSHLKCTSCVTCDP--NADILQNCNATQNAQC 7323



>scaffold58435 length=2429
          Length = 2429

 Score = 35.8 bits (81), Expect = 1.5
 Identities = 23/74 (31%), Positives = 32/74 (43%), Gaps = 1/74 (1%)
 Frame = +1

Query: 99   SKCR-RCRLCDEGHGLEVEINCTRTQNTKCRCKPNFFCNSTVCEHCDPCTKCEHGIIKEC 157
            SKCR RCR C        +  C+R    +CRC+    C  + C     C+KC       C
Sbjct: 1768 SKCRCRCR-CSRCRCRCSKCRCSRCSKCRCRCRCRCRCSRCRCRCRCSKC------RC 1926

Query: 158  TLTSNTKCKEEGSR 171
            +  S  +C+   SR
Sbjct: 1927 SRCSKCRCRCRCSR 1968



>scaffold15527 length=11692
          Length = 11692

 Score = 35.8 bits (81), Expect = 1.5
 Identities = 20/57 (35%), Positives = 25/57 (43%), Gaps = 1/57 (1%)
 Frame = -3

Query: 76   NGDEPDCVPCQEGKEYTDKAHFSSKCRRCRLCDEGHGLEVEINCTRTQNTKC-RCKP 131
            NG E  C PC EG     K+    +C RC  CD     E  + C+   N  C +C P
Sbjct: 8567 NGFEQICEPCDEGYYQDRKSSSLVRCYRCITCDP--LAETTLECSPLHNAGCGKCPP 8403



>scaffold12257 length=24509
          Length = 24509
```

```
 Score = 35.8 bits (81), Expect = 1.5
 Identities = 19/80 (23%), Positives = 33/80 (41%), Gaps = 2/80 (2%)
 Frame = -3

Query: 88    GKEYTDKAHFSSKCRRCRLCDEGHGLEVEINCTRTQNTKCRCKPNFFCNSTVCEHCDPCT 147
             G  ++D  + +SKC  C  C     ++ +  CT   +  C C    +      + C PC
Sbjct: 18207 GVNFSDTNNPTSKCVDCLSCHSTKEVQTK-ECTIESDAVCTCIEGSYYLDLGLDSCQPCR 18031

Query: 148   KCEHGIIK--ECTLTSNTKC 165
             +C    ++   C  T N +C
Sbjct: 18030 ECGASKVEIIPCNATQNREC 17971
```

```
>scaffold00596 length=176950
          Length = 176950

 Score = 35.8 bits (81), Expect = 1.5
 Identities = 21/83 (25%), Positives = 37/83 (44%)
 Frame = +3

Query: 221   AINLSDVDLSKYITTIAGVMTLSQVKGFVRKNGVNEAKIGEIKNDNVQDTAEQKVQLLRN 280
             A+ +SD  L +   ++ G         F+RK G+N  ++   +    Q  +   +++LR
Sbjct: 78927 AVRISDSQLEQLAKSLLGADP-QDTDAFLRKLGINSIEVDRYRGSGRQRESSGIMRMLRA 79103

Query: 281   WHQLHGKKEAYDTLIKDLKKANL 303
             W      K+    L+  LKKA L
Sbjct: 79104 WRDKTNDKKQKSELLAALKKAKL 79172
```

```
>scaffold02627 length=124852
          Length = 124852

 Score = 35.4 bits (80), Expect = 2.0
 Identities = 17/60 (28%), Positives = 31/60 (51%)
 Frame = +1

Query: 253   GVNEAKIGEIKNDNVQDTAEQKVQLLRNWHQLHGKKEAYDTLIKDLKKANLCTLAEKIQT 312
             G+ E ++   +   + +  E   Q+L  WHQ    ++    LI+ L+K  L +L EK++T
Sbjct: 95479 GLTEVRLQNWERQYMMNIEEAARQMLFTWHQNQSPRDERRLLIESLEKCKLRSLGEKVRT 95658
```

```
>scaffold02238 length=99018
          Length = 99018

 Score = 35.4 bits (80), Expect = 2.0
 Identities = 25/92 (27%), Positives = 44/92 (47%), Gaps = 16/92 (17%)
 Frame = -2

Query: 98    SSKCRRCRL----CDEGHGLEVEINC-----TRTQNTKCR---CKPNFFCNSTVCEHCDP 145
             SSKCRRC+     C     + +         +R+++++CR   CK N  C S+ C
Sbjct: 41654 SSKCRRCKCTSNRCKSSECINIRCRSSRC*KSRSRHSRCRSIKCKKNSRCRSSRCR---- 41487

Query: 146   CTKCEHGIIKECTLTSN----TKCKEEGSRSN 173
              +KC++   +     +N    ++C+  GSRS+
Sbjct: 41486 SSKCKNSRCRSSA*RNNRCRSSRCRSCGSRSS 41391
```

```
>scaffold04742 length=51821
          Length = 51821

 Score = 35.0 bits (79), Expect = 2.6
 Identities = 17/56 (30%), Positives = 25/56 (44%), Gaps = 4/56 (7%)
 Frame = -2

Query: 101   CRRCRLCDEGHGLEVEINCTRTQNTKCRCKPNFF----CNSTVCEHCDPCTKCEHG 152
             C+ C  C EG  +    NC    N +   +  +     C+ T+CE  D C+KC  G
Sbjct: 31705 CKWCYNCPEGACIPTAANCMEEHNCQVETQKEYHDRSDCSETICEASD-CSKCTTG 31541



>scaffold03852 length=78629
          Length = 78629

 Score = 34.7 bits (78), Expect = 3.4
 Identities = 20/69 (28%), Positives = 33/69 (47%)
 Frame = -1

Query: 98    SSKCRRCRLCDEGHGLEVEINCTRTQNTKCRCKPNFFCNSTVCEHCDPCTKCEHGIIKEC 157
             SS+C RCR    G        C+R +  +CRC     C+   C  C  C++C  G  + C
Sbjct: 51257 SSRCSRCR---RGRCRCSRCRCSRCRRGRCRC-TRCRCSRCRCSRC-RCSRCTSGRCR-C 51096

Query: 158   TLTSNTKCK 166
             ++   ++C+
Sbjct: 51095 SICRCSRCR 51069



>scaffold02123 length=107523
          Length = 107523

 Score = 34.7 bits (78), Expect = 3.4
 Identities = 23/103 (22%), Positives = 41/103 (39%)
 Frame = +3

Query: 69   KARDCTVNGDEPDCVPCQEGKEYTDKAHFSSKCRRCRLCDEGHGLEVEINCTRTQNTKCR 128
            + R C        C  C+  +    +   SS+CR C  C           C+R + ++CR
Sbjct: 3333 RCRCCRCRSSRYRCSRCRCSRCRCSRCR-SSRCR-CSRCRSSRYRSSRCRCSRCRRSRCR 3506

Query: 129  CKPNFFCNSTVCEHCDPCTKCEHGIIKECTLTSNTKCKEEGSR 171
            C     C S+ C     C++C     + C+   +++C+    R
Sbjct: 3507 CSR---CRSSRCR----CSRCRSSRCR-CSRCRSSRCRSRRCR 3611



>Scaff31
          Length = 732407

 Score = 34.7 bits (78), Expect = 3.4
 Identities = 28/105 (26%), Positives = 41/105 (39%), Gaps = 15/105 (14%)
 Frame = -1

Query: 82     CVPCQEGKEYTDKAHFS----SKCR--RCRLCDEGHGLEVEINCTRTQNTKCRC----KP 131
              C+ C+  +    +   S    S+CR  RCR C           C+R + T+CRC
Sbjct: 471218 CIRCRGSRCRCSRCRGSRCRCSRCRGSRCRCC--------RFRCSRCRCTRCRCIRCRGS 471063

Query: 132    NFFCNSTVCEHCD-PCTKCE----HGIIKECTLTSNTKCKEEGSR 171
                 C+    C  C   CT+C      G    C     ++C+  GSR
```

```
Sbjct: 471062 RCRCSRCRCSRCRCRCTRCRCIRCRGSRCRCIRCRCSRCRGRGSR 470928



>scaffold54882 length=2536
          Length = 2536

 Score = 34.3 bits (77), Expect = 4.4
 Identities = 19/74 (25%), Positives = 31/74 (41%), Gaps = 6/74 (8%)
 Frame = -2

Query: 99  SKCRRCRLCDEGHGLEVEINCTRTQNTKCRCKPNFFCNSTVCEHCDP------CTKCEHG 152
           S+C RCR C           C+R ++++CRC+    C+   C  C        C++C  G
Sbjct: 747 SRCSRCR-CSRCRCSRGRCRCSRCRSSRCRCR----CSRCRCSRCRSSRCRCRCSRCRCG 583

Query: 153 IIKECTLTSNTKCK 166
               C      +C+
Sbjct: 582 ---RCRCICRCRCR 550



>scaffold45295 length=2872
          Length = 2872

 Score = 33.9 bits (76), Expect = 5.8
 Identities = 34/127 (26%), Positives = 44/127 (34%), Gaps = 23/127 (18%)
 Frame = -1

Query: 55  DGQFCHKPCPPGERKARDCTVNGDEPDCVPCQEGKEYTDKAHFSSKCRRCRLCDEGHGLE 114
           DG  C+  C  G     +C    DE D  PCQ G   TD  +       C +  EG   E
Sbjct: 727 DGYTCN--CVSGYG-GENCEYEIDECDSGPCQNGANCTD--YLDGYVCSCPMGFEGDNCE 563

Query: 115 VEIN------CTRTQ-------NTKCRCKPNFF----------CNSTVCEHCDPCTKCEH 151
           V+I+      C           +  C C P F           C S  C++  PC    +
Sbjct: 562 VDIDYCIDNLCENGALCVDGLLDYTCECLPGFDGQYCRVNINECASDPCQYGSPCFDEVN 383

Query: 152 GIIKECT 158
           G    CT
Sbjct: 382 GYFCNCT 362



>scaffold13431 length=14941
          Length = 14941

 Score = 33.5 bits (75), Expect = 7.6
 Identities = 18/52 (34%), Positives = 22/52 (42%)
 Frame = +3

Query: 76   NGDEPDCVPCQEGKEYTDKAHFSSKCRRCRLCDEGHGLEVEINCTRTQNTKC 127
            NG E  C PC +G           +C RC  CDE    E    C+   NT+C
Sbjct: 4995 NGFEQICEPCPDGYYQGGTRSDLVRCNRCITCDE--LAETVQECSSVNNTEC 5144



>scaffold14216 length=23502
          Length = 23502

 Score = 33.1 bits (74), Expect = 9.9
 Identities = 20/78 (25%), Positives = 34/78 (43%), Gaps = 4/78 (5%)
```

```
 Frame = +2

Query: 98   SSKCR----RCRLCDEGHGLEVEINCTRTQNTKCRCKPNFFCNSTVCEHCDPCTKCEHGI 153
            SS+CR    RC  C           C+R ++++CRC   +  +   C  C  C++C
Sbjct: 3482 SSRCRCSRCRCSRCRCSRCRCSRCRCSRCRSSRCRCS-RYISSRCSCSRCS-CSRC---- 3643

Query: 154  IKECTLTSNTKCKEEGSR 171
               C+  S ++C+    R
Sbjct: 3644 --RCSRCSCSRCRSSRCR 3691



>scaffold07782 length=48146
          Length = 48146

 Score = 33.1 bits (74), Expect = 9.9
 Identities = 26/115 (22%), Positives = 46/115 (40%), Gaps = 23/115 (20%)
 Frame = +1

Query: 77    GDEP-DCVPCQEGKEYTDKA--------HFSSKCRRCRLCDEGHGLEVEINC-TRTQNTK 126
             G++P +C  C +G  ++D++        H   K  +C  CD+    +  +NC  RT   +
Sbjct: 39277 GEKPYECSYCDKG--FSDQSSLTIHLRIHTGEKPYQCSYCDKAFSCQSSLNCHVRTHTGE 39450

Query: 127   -----CRCKPNFFCNSTVCEHC--------DPCTKCEHGIIKECTLTSNTKCKEE 168
                  C C   F     +  H           C+ C  G  ++ +LTS+ +   E
Sbjct: 39451 KPYQCCYCDKGFSQQGDLTRHVRIHTGEKPHQCSYCNKGFSRQSSLTSHVRIHTE 39615



>scaffold04496 length=88722
          Length = 88722

 Score = 33.1 bits (74), Expect = 9.9
 Identities = 14/36 (38%), Positives = 19/36 (52%), Gaps = 3/36 (8%)
 Frame = +1

Query: 133   FFCNSTVCEHCDPCTKCEHGIIK---ECTLTSNTKC 165
             F C+ST+C     C+KC  G I+    C    +TKC
Sbjct: 19318 FSCSSTLCSSYSTCSKCSRGTIRTTCTCPTCPSTKC 19425



>scaffold01666 length=137803
          Length = 137803

 Score = 33.1 bits (74), Expect = 9.9
 Identities = 33/119 (27%), Positives = 41/119 (34%), Gaps = 11/119 (9%)
 Frame = +2

Query: 59    CHKPCPPGERKARDCTVNGDEPDCVPCQEGKEYTDKAHFSSKC-RRCRLCDEGHGLEVEI 117
             C +PC         C +    P C+ C++            KC RRCR     H
Sbjct: 34868 CGEPCQKRLICGHTCGLKCGVP-CLGCKQ------------KCLRRCRHSQCKHPCSEPC 35008

Query: 118   N-CTRTQNTKCRCKPNFFCNSTVCEHCD------PC---TKCEHGIIKECTLTSNTKCK 166
               C + N KC    +  C     CE CD      PC    KCEH  I  C      KC+
Sbjct: 35009 EPCMKRCNWKC---DHHECKKLCCEPCDRPPCNEPCPKKRKCEHSCIGLCGEICPNKCR 35176
```

```
  Database: Pliv_genome_110914
    Posted date:  Dec 5, 2014 10:59 AM
  Number of letters in database: 1,213,013,516
  Number of sequences in database:  77,240

Lambda     K      H
   0.315    0.131    0.395

Gapped
Lambda     K      H
   0.267   0.0410    0.140


Matrix: BLOSUM62
Gap Penalties: Existence: 11, Extension: 1
Number of Hits to DB: 531,215,305
Number of Sequences: 77240
Number of extensions: 9386839
Number of successful extensions: 41031
Number of sequences better than 10.0: 26
Number of HSP's better than 10.0 without gapping: 6586
Number of HSP's successfully gapped in prelim test: 1775
Number of HSP's that attempted gapping in prelim test: 30453
Number of HSP's gapped (non-prelim): 17959
length of query: 335
length of database: 404,337,838
effective HSP length: 126
effective length of query: 209
effective length of database: 394,605,598
effective search space: 82472569982
effective search space used: 82472569982
frameshift window, decay const: 40,  0.1
T: 13
A: 40
X1: 16 ( 7.3 bits)
X2: 38 (14.6 bits)
X3: 64 (24.7 bits)
S1: 41 (21.6 bits)
S2: 74 (33.1 bits)
```

NCBI **BLAST Search Results** BLAST Entrez ?

TBLASTN 2.2.26 [Sep-21-2011]

Reference:
Altschul, Stephen F., Thomas L. Madden, Alejandro A. Schäffer,
Jinghui Zhang, Zheng Zhang, Webb Miller, and David J. Lipman (1997),
"Gapped BLAST and PSI-BLAST: a new generation of protein database search
programs", Nucleic Acids Res. 25:3389-3402.

dbase=Pliv_genome_110914
77,240 sequences; 1,213,013,516 total letters

Query= Hs_TRADD_XP_016879304
(312 letters)

Distribution of 3 Blast Hits on the Query Sequence

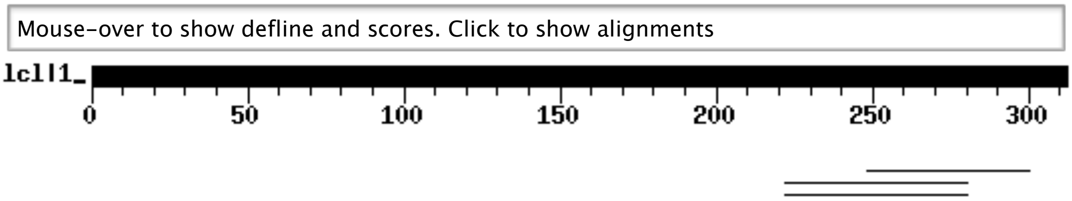

```
                                                                 Score    E
Sequences producing significant alignments:                      (bits) Value

scaffold01222  length=136942                                       34    4.0
scaffold02856  length=80876                                        33    8.8
scaffold01014  length=146114                                       33    8.8


>scaffold01222 length=136942
          Length = 136942

 Score = 34.3 bits (77), Expect = 4.0
 Identities = 18/53 (33%), Positives = 31/53 (58%)
 Frame = -2

Query: 249   LDSLAYEYEREGLYEQAFQLLRRFVQAEGRRATLQRLVEALEENELTSLAEDL 301
             +D++ YE+   E   EQA+Q+L   + Q   G++AT + L   AL  E +     A ++
Sbjct: 85416 VDNIVYEHRYERSSEQAYQVLLEWKQRRGQQATKKVLANALWEAKCYDAARNV 85258




>scaffold02856 length=80876
          Length = 80876

 Score = 33.1 bits (74), Expect = 8.8
 Identities = 21/59 (35%), Positives = 31/59 (52%)
 Frame = +1

Query: 223   ARSVGLKWRKVGRSLQRGCRALRDPALDSLAYEYEREGLYEQAFQLLRRFVQAEGRRAT 281
```

```
                 A+ VG +W+ +GR L    R L+       A E E +   EQAF +L  + + EG+ AT
Sbjct: 68356 AQQVGDQWQDLGRELGLSERELK-------AIERENDDRPEQAFVMLHNWSEQEGKDAT 68511




>scaffold01014 length=146114
          Length = 146114

 Score = 33.1 bits (74), Expect = 8.8
 Identities = 21/59 (35%), Positives = 31/59 (52%)
 Frame = +1

Query: 223   ARSVGLKWRKVGRSLQRGCRALRDPALDSLAYEYEREGLYEQAFQLLRRFVQAEGRRAT 281
             A+ VG +W+ +GR L    R L+       A E E +   EQAF +L  + + EG+ AT
Sbjct: 96415 AQQVGDQWQDLGRELGLSERELK-------AIERENDDRPEQAFVMLHNWSEQEGKDAT 96570




  Database: Pliv_genome_110914
    Posted date:  Dec 5, 2014 10:59 AM
  Number of letters in database: 1,213,013,516
  Number of sequences in database:  77,240

Lambda     K      H
   0.319    0.135    0.395

Gapped
Lambda     K      H
   0.267   0.0410    0.140


Matrix: BLOSUM62
Gap Penalties: Existence: 11, Extension: 1
Number of Hits to DB: 299,219,583
Number of Sequences: 77240
Number of extensions: 2896199
Number of successful extensions: 9648
Number of sequences better than 10.0: 3
Number of HSP's better than 10.0 without gapping: 1502
Number of HSP's successfully gapped in prelim test: 250
Number of HSP's that attempted gapping in prelim test: 7927
Number of HSP's gapped (non-prelim): 2442
length of query: 312
length of database: 404,337,838
effective HSP length: 125
effective length of query: 187
effective length of database: 394,682,838
effective search space: 73805690706
effective search space used: 73805690706
frameshift window, decay const: 40,  0.1
T: 13
A: 40
X1: 16 ( 7.4 bits)
X2: 38 (14.6 bits)
X3: 64 (24.7 bits)
S1: 41 (21.8 bits)
S2: 74 (33.1 bits)
```
