# Supplementary figures and images for: Identification of Cell Death Genes in Sea Urchin Paracentrotus lividus and Their Expression Patterns during Embryonic Development
Source: Genome Biol Evol. 2019 Jan 29;11(2):586–96. doi: 10.1093/gbe/evz020 (PMC6394757; doi:10.1093/gbe/evz020)

Aifm1

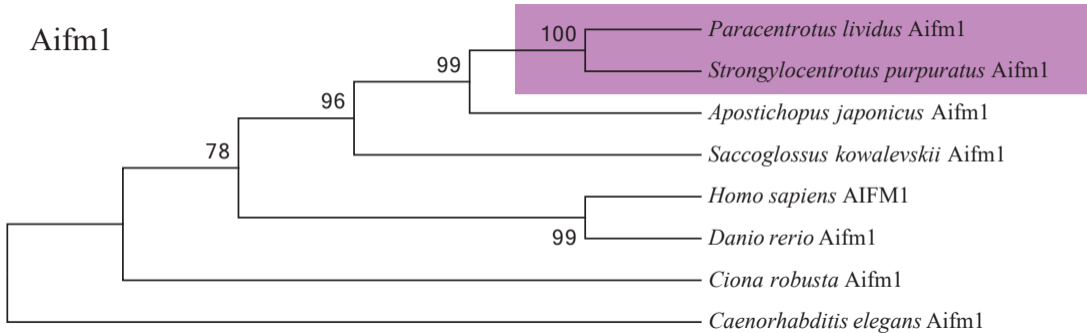

## Bax and Bcl2

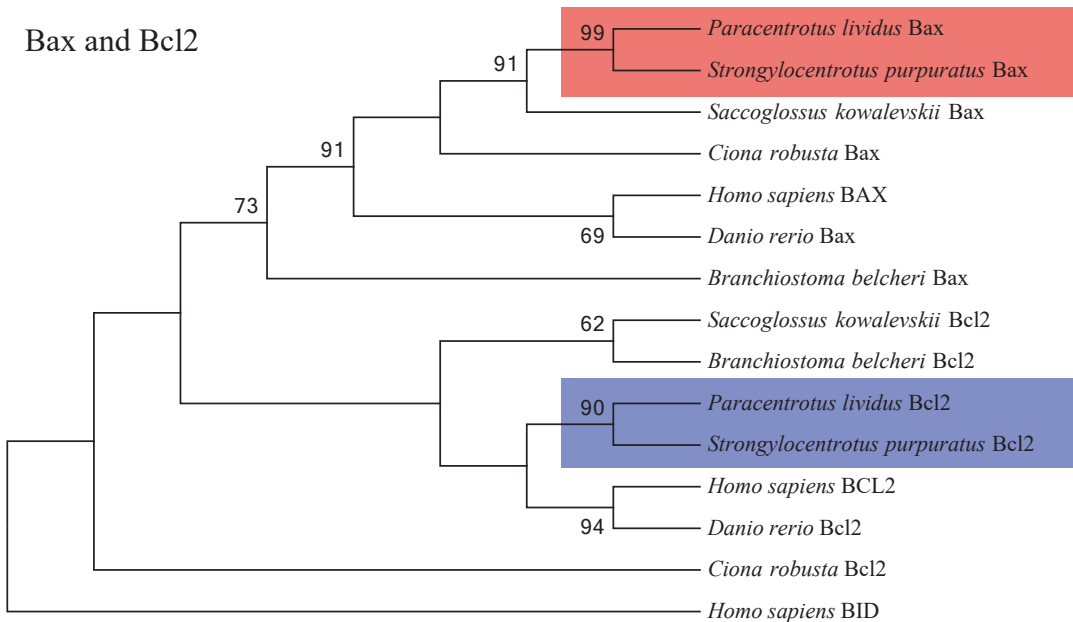

Parp

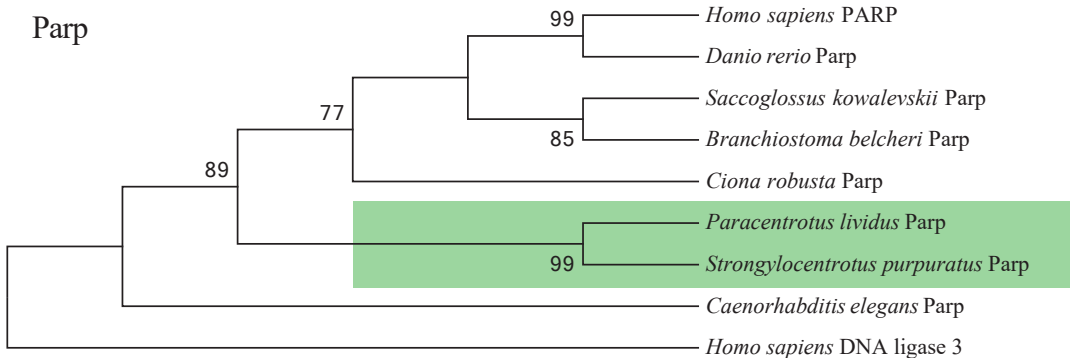

Pink

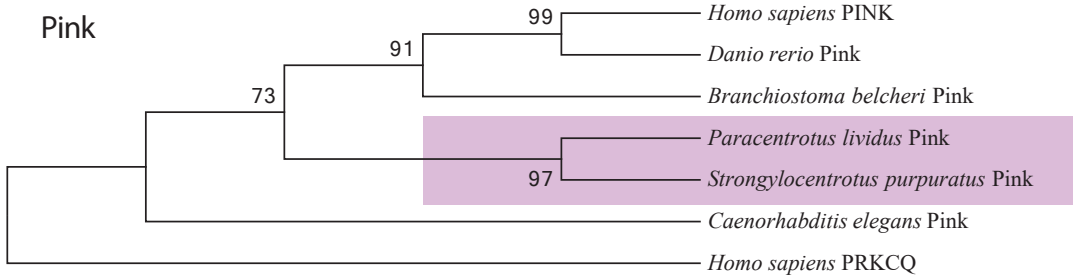

Supplement: Supplementary Data [file evz020_supp.zip › Supplementary information IV.pdf]
